# Supplementary material for: Experimental development of lightweight manipulators with improved design cycle time that leverages off-the-shelf robotic arm components
Source: PLoS One. 2024 Jul 18;19(7):e0305379. doi: 10.1371/journal.pone.0305379 (PMC11257280; doi:10.1371/journal.pone.0305379)
Supplement: Attachment — Submitted filename: PONE-D-23-31418_R1.pdf [file pone.0305379.s002.pdf]

PLOS ONE

Experimental Development of Lightweight Manipulators with Improved Design Cycle Time that leverages off-the-shelf Robotic Arm Components

--Manuscript Draft--

**Manuscript Number:**	PONE-D-23-31418R1
**Article Type:**	Research Article
**Full Title:**	Experimental Development of Lightweight Manipulators with Improved Design Cycle Time that leverages off-the-shelf Robotic Arm Components
**Short Title:**	Robotic Manipulator - Design and Experimentation
**Corresponding Author:**	Jamshed Iqbal, PhD<br>University of Hull<br>Hull, County (optional) UNITED KINGDOM
**Keywords:**	Lightweight robot; cobot; robot design process
**Abstract:**	The growing market for lightweight robots inspires new use-cases, such as collaborative manipulators for human-centered automation. However, widespread adoption faces obstacles due to high R&D costs and longer design cycles, although rapid advances in mechatronic engineering have effectively narrowed the design space to affordable robot components, turning the development of lightweight robots into a component selection and integration challenge. Recognizing this transformation, we demonstrate a practical framework for designing lightweight industrial manipulators using a case-study of indigenously developed 5 Degrees-of-Freedom (DOF) cobot prototype. Our framework incorporates off-the-shelf sensors, actuators, gears, and links for Design for Manufacturing and Assembly (DFMA), along with complete virtual prototyping. The design cycle time is reduced by approximately 40% at the cost of cobot real-time performance deviating within 2.5% of the target metric. Our physical prototype, having repeatability of 0.05mm calculated as per the procedure defined in ISO 9283:1998, validates the cost-effective nature of the framework for creating lightweight manipulators, benefiting robotic startups, R&D organizations, and educational institutes without access to expensive in-house fabrication setups.
**Order of Authors:**	Muhammad Rzi Abbas
	Muhammad Ahsan, PhD
	Jamshed Iqbal, PhD
**Response to Reviewers:**	Response to the reviewers file is attached for your kind review. Thanks a lot.
**Additional Information:**	
**Question**	**Response**
**Financial Disclosure**<br><br>Enter a financial disclosure statement that describes the sources of funding for the work included in this submission. Review the submission guidelines for detailed requirements. View published research articles from *PLOS ONE* for specific examples.<br><br>This statement is required for submission and **will appear in the published article** if the submission is accepted. Please make sure it is accurate.	The author(s) received no specific funding for this work.

Powered by Editorial Manager® and ProduXion Manager® from Aries Systems Corporation

**Funded studies**<br>Enter a statement with the following details:<br>• Initials of the authors who received each award<br>• Grant numbers awarded to each author<br>• The full name of each funder<br>• URL of each funder website<br>• Did the sponsors or funders play any role in the study design, data collection and analysis, decision to publish, or preparation of the manuscript?<br><br>Did you receive funding for this work?	
**Competing Interests**<br><br>Use the instructions below to enter a competing interest statement for this submission. On behalf of all authors, disclose any competing interests that could be perceived to bias this work—acknowledging all financial support and any other relevant financial or non-financial competing interests.<br><br>This statement is required for submission and **will appear in the published article** if the submission is accepted. Please make sure it is accurate and that any funding sources listed in your Funding Information later in the submission form are also declared in your Financial Disclosure statement.<br><br>View published research articles from *PLOS ONE* for specific examples.	The authors have declared that no competing interests exist.

Powered by Editorial Manager® and ProduXion Manager® from Aries Systems Corporation

**NO authors have competing interests**<br><br>Enter: *The authors have declared that no competing interests exist.*<br><br>**Authors with competing interests**<br><br>Enter competing interest details beginning with this statement:<br><br>*I have read the journal's policy and the authors of this manuscript have the following competing interests: [insert competing interests here]*<br><br>* typeset	
**Ethics Statement**<br><br>Enter an ethics statement for this submission. This statement is required if the study involved:<br><br>• Human participants<br>• Human specimens or tissue<br>• Vertebrate animals or cephalopods<br>• Vertebrate embryos or tissues<br>• Field research<br><br>Write "N/A" if the submission does not require an ethics statement.<br><br>General guidance is provided below. Consult the submission guidelines for detailed instructions. **Make sure that all information entered here is included in the Methods section of the manuscript.**	N/A

Powered by Editorial Manager® and ProduXion Manager® from Aries Systems Corporation

**Format for specific study types**<br><br>**Human Subject Research (involving human participants and/or tissue)**<br>• Give the name of the institutional review board or ethics committee that approved the study<br>• Include the approval number and/or a statement indicating approval of this research<br>• Indicate the form of consent obtained (written/oral) or the reason that consent was not obtained (e.g. the data were analyzed anonymously)<br><br>**Animal Research (involving vertebrate animals, embryos or tissues)**<br>• Provide the name of the Institutional Animal Care and Use Committee (IACUC) or other relevant ethics board that reviewed the study protocol, and indicate whether they approved this research or granted a formal waiver of ethical approval<br>• Include an approval number if one was obtained<br>• If the study involved *non-human primates*, add *additional details* about animal welfare and steps taken to ameliorate suffering<br>• If anesthesia, euthanasia, or any kind of animal sacrifice is part of the study, include briefly which substances and/or methods were applied<br><br>**Field Research**<br><br>Include the following details if this study involves the collection of plant, animal, or other materials from a natural setting:<br>• Field permit number<br>• Name of the institution or relevant body that granted permission	
**Data Availability**<br><br>Authors are required to make all data underlying the findings described fully available, without restriction, and from the time of publication. PLOS allows rare exceptions to address legal and ethical concerns. See the PLOS Data Policy and FAQ for detailed information.	Yes - all data are fully available without restriction

A Data Availability Statement describing where the data can be found is required at submission. Your answers to this question constitute the Data Availability Statement and **will be published in the article**, if accepted.<br><br>**Important:** Stating 'data available on request from the author' is not sufficient. If your data are only available upon request, select 'No' for the first question and explain your exceptional situation in the text box.<br><br>Do the authors confirm that all data underlying the findings described in their manuscript are fully available without restriction?	
**Describe where the data may be found in full sentences. If you are copying our sample text, replace any instances of XXX with the appropriate details.**<br><br>• If the data are **held or will be held in a public repository**, include URLs, accession numbers or DOIs. If this information will only be available after acceptance, indicate this by ticking the box below. For example: *All XXX files are available from the XXX database (accession number(s) XXX, XXX.)*.<br>• If the data are all contained **within the manuscript and/or Supporting Information files**, enter the following: *All relevant data are within the manuscript and its Supporting Information files.*<br>• If neither of these applies but you are able to provide **details of access elsewhere**, with or without limitations, please do so. For example:<br><br>*Data cannot be shared publicly because of [XXX]. Data are available from the XXX Institutional Data Access / Ethics Committee (contact via XXX) for researchers who meet the criteria for access to confidential data.*<br><br>*The data underlying the results presented in the study are available from (include the name of the third party*	All relevant data are within the manuscript.

and contact information or URL).<br>• This text is appropriate if the data are owned by a third party and authors do not have permission to share the data.<br><br>* typeset	
Additional data availability information:	

Manuscript

Click here to access/download;Manuscript;Clean_Manuscript.docx

Experimental Development of Lightweight Manipulators with Improved Design Cycle Time that leverages off-the-shelf Robotic Arm Components

Muhammad Rzi Abbas[1], Muhammad Ahsan[1], Jamshed Iqbal[2]
[1]Department of Mechatronics and Control Engineering, University of Engineering and Technology, Lahore, Pakistan
[2]School of Computer Science, Faculty of Science and Engineering, University of Hull, Hull, United Kingdom

Abstract

The growing market for lightweight robots inspires new use-cases, such as collaborative manipulators for human-centered automation. However, widespread adoption faces obstacles due to high R&D costs and longer design cycles, although rapid advances in mechatronic engineering have effectively narrowed the design space to affordable robot components, turning the development of lightweight robots into a component selection and integration challenge. Recognizing this transformation, we demonstrate a practical framework for designing lightweight industrial manipulators using a case-study of indigenously developed 5 Degrees-of-Freedom (DOF) cobot prototype. Our framework incorporates off-the-shelf sensors, actuators, gears, and links for Design for Manufacturing and Assembly (DFMA), along with complete virtual prototyping. The design cycle time is reduced by approximately 40% at the cost of cobot real-time performance deviating within 2.5% of the target metric. Our physical prototype, having repeatability of 0.05mm calculated as per the procedure defined in ISO 9283:1998, validates the cost-effective nature of the framework for creating lightweight manipulators, benefiting robotic startups, R&D organizations, and educational institutes without access to expensive in-house fabrication setups.

Keywords

Lightweight robot, Cobot, Robot Design Process

Section I

Introduction

The field of robotics has undergone a significant evolution, particularly with the emergence of lightweight robots, aligning seamlessly with the requisites of Industry 5.0 (1). This progression has paved the way for substantial advancements in collaborative robotics, where lightweight robots, often referred to as "cobots," are tailored for harmonious human-robot

interaction in applications ranging from industrial collaboration to medical-related scenarios (2-6). These versatile entities boast characteristics that position them as adaptable collaborators within cooperative work environments. Their intrinsic attributes, characterized by reduced speeds and inertias, make them particularly conducive for seamless interaction with human counterparts, leading to a broadening spectrum of applications across diverse domains. From assembly line installations (7) to medical setups (8), from education sector (9) to space endeavors (10) , these robots have found their utility across varied industries. The escalating demand for automation positions lightweight robots as pivotal players across industrial sectors (1). In this evolving landscape, our research takes on heightened significance, strategically addressing the imperative for streamlined design and development of lightweight robots, a cornerstone in facilitating the transition toward Industry 5.0-aligned industrial and R&D ecosystems.

Conventional engineering product design cycle (11) is illustrated in Fig 1 which is used to argue about robotic product development cycle. The whole process may be divided into seven major stages as labeled by circled digits in the figure.

Fig 1: Typical Robot Design and Development Cycle. The table in the figure shows typically what percentage of total design cycle time (12-14) is spent on each stage. The portions enclosed in Red Boxes are either eliminated or simplified through the proposed scheme.

- Stage 1 is the initial stage where the concept of the robot is formulated, and its basic design is outlined. It involves defining the robot's purpose, capabilities, and basic architecture. Typically, 10%[1] (12-14) of the total design cycle time is spent in this stage.
- Stage 2 relates to the detailed design of the robot's components, such as mechanical, electrical, and software aspects. Planning for the manufacturing and assembly processes also occurs in this stage, and it takes around 20% of the total design cycle time.
- Stage 3 implements the initial design, usually in simulated environments, which is a quicker method, or sometimes a physical prototype is also developed, that normally takes longer. Physical prototype development consumes around 15% of the design cycle time.
- Stage 4 tests and iterates the prototype based on several performance parameters. Typically, 15% of the design cycle time is spent on this activity.
- Stage 5 starts once the design is optimized and tested. The final version of the robot design is prepared for manufacturing. This includes sourcing components, manufacturing, and assembly. Normally 20% of the total design cycle time is utilized in this stage.
- Stage 6 tests a validates the manufactured robot to make it deployment ready. This stage typically consumes 15% of the total design cycle time.

---

[1] These values are based on general industry knowledge and practices. The breakdown of time spent on different stages of robot development can vary widely depending on the specific project, organization, and goals. These percentages are meant to give a rough idea of how time may classically be allocated in a typical robot development process.

- Stage 7 is related to production of documentation, user manuals, training material and deployment of the robot in the intended environment. This stage lasts around 5% of the total design cycle time.

Considering the extensive range of commercially available components, it would be both economical and timesaving for developers to simulate robotic manipulators exclusively composed of such components. The design approach presented in this paper modifies the robot development process along these lines, hence reducing the complexity and required time. First, stage 1 is reduced to only gathering of the robot specifications. The time needed to develop initial conceptual design has been reduced because of the developed library of components. Second, the modeling of the subcomponents allows for virtual prototyping, eliminating the need for physical prototype development, saving around 20% of the total design cycle time. Moreover, the developed library eliminates the requirement of subcomponents' performance analysis, further saving valuable development time. Lastly, the testing and validation of the manufactured robot is eliminated because the virtual prototype is made of all commercially available or produced components. The Pearson's Correlation Coefficient, presented in the experimentation section of this paper, between the performance of the virtual prototype and the developed robot confirms this claim.

This research work focuses on the simulation of hollow shaft Integrated Drive Joints (IDJs) integrated into lightweight robotic manipulators designed to operate as cobots. The developed library encompasses models of several commercially available subcomponents. Within the simulation environment, the IDJ models and the manipulator's structure are utilized to identify the optimal components for the integrated drive joints. Moreover, the simulation of the complete structure enables the consideration of coupled dynamics throughout the entire system, which is significant to control system engineers. While a single IDJ exhibits distinct performance characteristics, coupling multiple joints introduces coupled dynamic effects across all joints (15, 16). These couplings can be mitigated at the structural level through the appropriate selection of gearing systems or addressed at the software level by fine-tuning the control system accordingly.

The simulation tool was developed using the MATLAB/Simulink R2021b environment, while the robot structure was modeled using SolidWorks 2021. Subsequently, based on the findings obtained from the simulation tool, a 5 degrees-of-freedom (DOF) lightweight robot was developed, as depicted in Fig2. Though the authors have also developed a 6-DOF lightweight robot by following the same footsteps, but for the sake of compactness of the article, the detailed analysis of 5-DOF robot is presented.

Fig 2: 5 DOF Robot's FBD (Left) and 5 DOF robot's hardware (Right).

The organization of the remaining manuscript is as follows: Section II describes the challenges involved in the in-house development of light-weight robots and then propose a suitable methodology for generating an IDJ model that includes a synergetic combination of

frameless brushless DC (BLDC) motor, strain wave gear and other subcomponents. Section III showcases the simulation results of the developed model, whereas Section IV details the performance results of the physically developed 5 DOF manipulator based on the in-horse manufactured integrated drive joint using the same hardware subcomponents as those used in the simulation. Lastly, Section V concludes the manuscript and steers the reader to possible avenues of future work.

Section II

Addressing challenges involved in the in-house development of Light-Weight Robots

In the current landscape of robot design methodologies, an extensive exploration of both bottom-up and top-down approaches is available in the literature. However, a notable gap persists as many of these analyses overlook the integration of commercially available, off-the-shelf components into the design process. While bottom-up strategies are apt for the development of generic multi-purpose robots, top-down approaches excel in crafting application-specific robotic solutions (17). The top-down paradigm initiates the design process with a clear focus on the final application, resulting in a more streamlined design space. Addressing this, recent studies (18) highlight the efficacy of top-down approaches in mitigating cyclic dependencies, particularly beneficial for the efficient design of mechatronic systems characterized by intricate interdependencies. Another pertinent contribution in this domain comes from a study on the optimization of reconfigurable collaborative robots, emphasizing modularity and structural considerations (19). This innovative top-down approach draws inspiration from the V-model employed in the automotive and aerospace industries, as introduced in (20). The optimization strategy revolves around enhancing the cost-effectiveness of development by optimizing the physical structure, consequently yielding savings in manufacturing materials. Additionally, a significant reduction in development time is achieved by capitalizing on established modular interfaces instead of customizing interfaces for each module, as detailed in (19). These contemporary studies collectively underscore the evolving landscape of design processes, shedding light on the nuanced benefits of incorporating off-the-shelf components within a top-down framework.

Like any robotic system, lightweight manipulators adhere to basic design principles and employ specific components to achieve their lightweight characteristics (21). The IDJ or Direct Drive Joint (DDJ) incorporates frameless BLDC Motors and Strain Wave Gears, distinct from conventional manipulators. Additionally, lightweight manipulators utilize differently designed links made from lightweight materials. Over the past two decades, researchers have proposed various drive unit designs suitable for lightweight robots (21-24). The design constraints imposed by the hollow shafts of drive units and of links connecting the drive units to form the kinematic chain, require specific component choices. For example, the flexspline of the strain wave gear and the lightweight structure of the robot introduce mechanical flexibility, necessitating the consideration of dynamic coupling among multiple drive units through lightweight links. Consequently, optimizing the subcomponents of drive units and links becomes a primary objective in the design and manufacturing of lightweight robots. Moreover, considering the

extensive experience in the development of robotic manipulator components (25, 26), it is prudent to leverage this existing knowledge to narrow down the design space.

To evaluate the performance of interconnected systems that incorporate multiple drive units and links, simulation tools are essential. However, the lack of simulation tools capable of simulating complete robotic systems with integrated drive joints forces developers to physically test and validate certain components. The discrepancy arises because designers often choose to simulate idealized systems or systems based on various assumptions in existing robot simulators (27, 28). There is a need to model complex systems using available hardware. For instance, when determining suitable gearing systems for a specific speed reduction task, a design engineer typically suggests gear specifications through calculations and simulations. However, these specific gearing systems might not be procurable or manufacturable. Conversely, it would be advantageous to explore the available gearing systems suitable for lightweight robotic manipulators, model them, and adapt other parts of the system to achieve the desired speed reduction. This concept extends beyond gearing systems to encompass motors, sensors, bearings, motor drives, and the robot structure as well.

In the realm of industrial robotics, decades of design and manufacturing experience have allowed for the identification of off-the-shelf components that can be utilized to construct high-performance functional manipulators. Adopting a counter approach, where the available components shape the design space, would be highly desirable if comprehensive simulated models of all commercially available components were readily accessible. Regrettably, the current landscape does not provide an extensive repertoire of simulated models, and even when a few such models do exist, there is a notable absence of a *unified and scalable platform* capable of testing the combinations of different components employed in the fabrication of critical elements, such as a single joint within a robotic manipulator or the manipulator as a complete system.

Fig 3 illustrates the comprehensive workflow of the proposed scheme. The end-user provides the system requirements, which serve as the basis for component selection, leading to the creation of a Computer Aided Design (CAD) model of the robot. This CAD model is subsequently imported into the Simulink environment, where it is subjected to predefined trajectories. Based on the evaluation of position, velocity, and torque tracking performance, frameless BLDC motors and/or strain wave gears may be substituted, if necessary. Once satisfactory performance is achieved in the simulation, the robot is fabricated and assembled utilizing the commercially available components identified during the simulation phase. To complete the entire process, a comparative analysis is conducted, contrasting the performance of the manufactured robot with that of the simulated robot.

Fig 3: Comprehensive workflow of the proposed scheme.

Methodology for Implementation of the Proposed Scheme

Developing a comprehensive simulation model that encompasses all components of a real-life IDJ, presents a complex undertaking. However, it becomes evident that the frameless BLDC motor and the gearing system are the two major components. Consequently, detailed models were constructed for these components, while simplified approximations were employed for smaller subcomponents like position encoders and bearings. This approach is logical as the inertia of the IDJ is primarily attributed to the BLDC motor and the gearing system, with the BLDC motor also consuming most of the electrical power. Thus, both the electrical and mechanical characteristics of the IDJ are primarily dictated by these two key components.

The development of models for the involved subcomponents necessitates consideration of several aspects. For instance, a motor requires modeling of both its electrical and mechanical attributes, whereas a gearing system pertains solely to mechanical aspects. However, different types of gearing systems exhibit distinct behaviors. For example, a planetary gear system lacks flexibility within its internal gears but possesses measurable backlash. On the other hand, a strain wave gear introduces flexibility due to its flex spline but does not exhibit backlash. Backlash and rigidity are just a few of the differing characteristics between these two types of gearing systems. Therefore, a comprehensive modeling of a gearing system requires the consideration and optimization of various parameters based on the specific requirements. However, to streamline the process, it is practical to focus solely on modeling the parameters of commercially available hardware components, bypassing the optimization step entirely. Consequently, developers can expedite the process by leveraging existing hardware components and utilizing their models within the simulation environment, effectively bridging the gap between simulation and actual hardware.

The subsequent discussion elaborates on the potential components that an IDJ may encompass. However, it is worth noting that certain components may be omitted in practice without compromising performance, albeit at the expense of non-performance-related features. Moreover, the simulated models utilize parameters sourced from component datasheets, which are typically freely available on the manufacturer's website.

Frameless BLDC Motor Model

Extensive research has been conducted on developing simulation models for various types of motors, including BLDC motors. Given the objective of this study to establish an effective combination of motor models, strain wave gears, and approximate other components and parameters, there is no need to reinvent the wheel. Hence, standard BLDC motor models have been utilized and adapted to create models for commercially available frameless BLDC motors.

In addition to the BLDC motor, the drive system used to operate it plays a crucial role. The choice of drive system determines the required feedback and the level of control it can exert over the motor's motion. Typically, commercially available BLDC motor drives implement speed

control through stator current control, employing a position sensor such as a rotary encoder or a hall sensor. The generalized mechanical system dynamics are represented by:

$$T_e = J\frac{d}{dt}\omega_r + F\omega_r + T_m$$

Where T_e represents the electromagnetic torque, J the inertia, ω_r the rotor speed, F the viscous friction, and T_m the load torque. These kinds of drives have an inner current loop that will take in the commanded torque and utilize the torque constant of the motor to generate suitable stator phase currents. Whereas the outer speed control loop takes in the load or applied torque and reference speed to figure out the electromagnetic torque as per the mechanical system's dynamic equation. Furthermore, commercial motor drives implement Proportional-Integral (PI), Proportional-Derivative (PD) or Proportional-Integral-Derivative (PID) controller for maintaining the desired speed-torque characteristics. These controllers are pre-tuned or may easily be autotuned using the damping factor and the desired response time. Fig 4 shows the block diagram of the overall control scheme for commercially available motor drives.

Fig 4: General control scheme implementing closed loop control at all three stages, that is, torque (current), velocity, and position.

In this study, BLDC motor controllers incorporating PI controllers at all three levels were employed: the innermost current loop, velocity loop, and outermost position loop. The inclusion of an integrator in servomechanisms ensures zero steady-state error and helps to mitigate noisy signals commonly encountered in lightweight robotic manipulators, where weak sensor signals are in close proximity to high-powered BLDC motors. Moreover, the choice of using PI controllers only is further supported by the fact that all different types of hardware motor controllers used in this study employ only PI controllers to control the BLDC motor. The PI controllers of the hardware motor controllers were tuned according to the manufacturer's specifications, while the simulation-based motor controllers were autotuned using the damping factor and desired response time, as outlined in (29). The damping factor is defined as follows:

$$\zeta = \frac{\omega_n K_p}{2K_i}$$

If $\zeta < 0.69$, then natural frequency is defined as:

$$\omega_n = \frac{-1}{\zeta \times T_{nl}}\log(0.05 \times \sqrt{1-\zeta^2})$$

Where if $\zeta \geq 0.69$, then natural frequency is defined as:

$$\omega_n = \frac{0.9257}{T_{nl}}e^{1.6341\times\zeta}$$

Strain Wave Gear Model

In this research, the modeling of strain wave gears encompasses several crucial parameters that can be sourced from either the manufacturer's datasheet or the CAD model. These include weight, moment and product of inertia, stiffness, damping constant, nominal torque, and nominal speed. These parameters form the foundational framework for the accurate representation of strain wave gears within the simulation environment, ensuring a comprehensive and precise analysis of their performance characteristics.

While most parameters can be directly extracted, the moment and product of inertia are obtained from the manufacturer-provided CAD model. The damping constant is influenced not only by the grease used in the gearing system but also by the friction in the bearings and overall component alignment. Therefore, an empirical determination of the damping constant was performed for one of the manufactured joints to account for manufacturing imperfections and friction. Standardizing the manufacturing process, materials, assembly processes, strain wave gear grease, and bearings will improve the approximation of the damping constant. The determined damping constant was incorporated into the simulated model to capture its effects.

Robot Structure

For modeling the structure of the robotic manipulator, SolidWorks 2021 was employed in this research. All necessary parts were modeled, and analysis was performed to establish the validity. IDJ was modeled using the supplier-provided CAD models of the included components, which were the frameless BLDC motor, strain wave gear, optical encoder, bearings, and joint housing that will encase all the components. Moreover, the links and the connection flanges were modeled and passed through stress-strain analysis for establishing the validity of the model subjected to the worst-case loading. For links and joint housings, we used Aluminum 6061-T6 material because of its strength and lightweight, while for connection flanges we used Alloy Steel having yield strength of $6.2\text{x}10^8$ N/m^2. The factor of safety for Aluminum links is 2.4, while for flanges its 4.3. Fig 5 shows stress analysis figure of one of the links being subjected to a maximum of $1.158\text{x}10^8$ N/m^2 stress, while Aluminum 6061-T6 has yield strength of $2.75\text{x}10^8$ N/m^2, hence achieving factor of safety of 2.4. Some covers which were not load bearing were later 3D printed, hence doesn't need any analysis.

Fig 5: Stress Analysis of one of the links showing maximum stress being applied on it in worst case.

The CAD model of the robot's structure generated in SolidWorks was then imported into MATLAB's environment for simulation. The CAD model considered the following parameters:

- Robot configuration
- Link weights
- Link moment and product of inertias
- Link lengths

Fig 6 illustrates one of the designed links and its integration with the joints to form the complete CAD model of the 5 DOF robot.

Fig 6: Exploded view of the 5 DOF robot showing Links, Joints, Connection flanges, and 3D printed covers.

The robotic manipulator consists of additional subcomponents within the integrated drive joint that have smaller weight and moment of inertia. These components, listed below, can be grouped together, and represented as a single model by combining their net weights and inertias, as their individual effects on the dynamics are relatively minor.

Other components

In addition to the previously mentioned components, there are various other components that may or may not be included in an integrated drive joint (30). These components, such as the miniaturized circular circuit board, position sensor, bearings, torque sensor, brake, and housing, have significantly lower weights and moment of inertias compared to the BLDC motor, strain wave gear, and robot's structure. For analysis, these components are combined into a single unit for simplicity. The forthcoming section elaborates on the aspects considered for modeling and subsequent manufacturing of the integrated drive joint.

Simulation Model Flow

The direct drive joint model was developed by individually modeling and combining commercially available subcomponents. The model includes a frameless BLDC motor, strain wave gear, incremental position encoder, hollow shaft, bearings, joint housing, and miscellaneous small parts. The electrical aspects of the frameless BLDC motor were modeled in Simulink MATLAB using the Brushless DC Motor Drive (AC7) block, which represents a standard current-controlled drive for BLDC motors with closed-loop speed control. The mechanical aspects of the motor and strain wave gears were modeled in SolidWorks. Table 1 presents the parameters used to model the frameless BLDC motor, extracted from commercially available datasheets. The developed simulator includes eight different frameless BLDC motors from T-Motor and Maxon, allowing for modeling of various motors without manufacturer dependence.

Table 1: List of BLDC Motor Parameters that were used for modeling the commercially available motors. Sample of three different BLDC Motors are shown.

Sr. #	Parameter	Unit	TMotor RI50	TMotor RI80	Maxon 588849
1	Resistance	Ohm	0.673	0.196	0.109
2	Inductance	mH	0.474	0.364	0.0665
3	BEMF Flat Area	rad	1.197	1.197	0.761
4	Torque Constant	mNm/A	99	127.3	80.7
5	Speed Constant	rad/s/V	10.11	7.85	12.36
6	Robot Inertia	g.cm^2	3500	6000	5300

7	Poles	-	7	7	11
8	Nominal Torque	mNm	500	1450	1010

Similarly, several parameters of strain wave gear were used to model its effect in the direct drive joint, out of which the gear ratio is defined in the motor block. Apart from gear ratio, the weight, moment and product of inertias, stiffness, nominal torque, and nominal speed parameters were defined and used in different blocks. Furthermore, the simulated direct drive joint model includes the weights and inertias of position encoder, bearings, joint housing, and the hollow shaft through which the power and communication wires are routed.

Fig 7 shows the section view of the CAD model of the simulated direct drive joint.

Fig 7: Section view of the CAD model of the assembled Integrated Drive Joint

Later, the simulated integrated drive joint was installed in the simulated robot structure, and the manipulator was driven on a defined trajectory. The position, velocity, and torque requirement graphs were plotted to establish the suitability of the simulated direct drive motor joint.

Manipulator Model

To evaluate the simulated integrated drive joint, it was integrated into a 5-DOF robotic manipulator model. This manipulator model was meticulously constructed in SolidWorks and seamlessly imported into MATLAB's Multibody environment. The development of the manipulator's model considered critical parameters, including robot configuration, link weights, link moment, product of inertias, and link lengths. These considerations were pivotal in ensuring the accuracy and effectiveness of the simulated manipulator's performance under various conditions.

Table 2 presents the parameters of the robot links and joints used to generate the simulation model. These parameters were obtained from the CAD model, which considers the commercially available material, its properties, and the machining processes involved. While importing the model from SolidWorks 2021 to the Simulink environment, the dimensions and shape of the links were preserved. However, the shape and size of the joints were approximated by a point mass while maintaining all other parameters.

Table 2: List of Parameters used for modeling the structure of the robot in Simulink Environment.

Sr. #	Manipulator Link		Manipulator Joint	
	**Parameter**	**Unit**	**Parameter**	**Unit**
1	Size and Shape	mm	Point Mass	-
1	Mass	kg	Mass	kg
2	Center of Mass	mm	Center of Mass	mm
3	Moments of Inertia	$g.cm^2$	Combined Moments of Inertia	$g.cm^2$

4	Products of Inertia	$g.cm^2$	Combined Products of Inertia	$g.cm^2$

Individual links of the robotic arm were imported into the Multibody environment and were assembled in the defined configuration to build the complete structure. Fig 8 shows the Simulink model for building the robotic arm in MATLAB.

Fig 8: Simulink SimScape Multibody model for building 5 DOF Robotic Arm

Section III

Simulation Experimentation

In the experimentation phase, direct drive joints featuring various commercially available frameless BLDC motors were integrated with the 5-DOF manipulator model. Trapezoidal velocity trajectories were generated as reference trajectories for different scenarios, and the simulated model was driven on these trajectories to evaluate the performance of the direct drive joints. Fig 9 provides a snapshot of the simulation running on a specific trajectory.

Fig 9: Snapshot of simulation of 5 DOF manipulator on a given trajectory.

To account for potential manufacturing inaccuracies, up to 10% random variations were introduced in the weight, inertia, and stiffness parameters of the simulated direct drive joint and robot's structure. These variations compensate for possible deviations that may arise during the actual manufacturing process, such as material and machining differences and assembly imperfections. Additionally, an empirically determined damping constant was incorporated to model frictional forces. A test joint was manufactured to evaluate manufacturing imperfections and determine frictional losses, which were then included in the simulated model. All joints of the 5-DOF manipulator were configured with a damping constant of 2 Nm/deg/s. Fig 10 illustrates a snapshot of a test joint.

Fig 10: IDJ Testing Setup

The simulation was conducted using the finalized parameters and SimScape Multibody model. The results were analyzed to determine if the simulated joints successfully followed the reference trajectories. Since a robotic manipulator consists of multiple joints and links, the focus was on analyzing the coupled dynamics of the entire structure rather than individual joint performance. Assessing the performance of each joint when interconnected through the robot's structure was deemed more meaningful. Moreover, For industrial robotic manipulators, primary sources of disturbance include environmental interaction, unmodeled dynamics, and

gravitational effects (31). All these sources of disturbance are encountered and treated at the system level. Fig 11 depicts the system-level Simulink model used to simulate the complete system.

Fig 11: System level Simulink Model for simulating the robotic manipulator and its joints.

The system model shown in Fig 11 outlines the simulation flow. A reference trajectory was generated in joint space and applied to the Multibody model of the robot. The model followed the trajectory, while position, velocity, and torque sensors captured corresponding signals. The sensed positions and torques were then used to control the BLDC motors through a PI-based control system. Joints that were capable of accurately following the desired position and torque profile exhibited minimal deviation between the output and reference signals. To quantitatively assess the similarity between the reference and simulated data, Pearson's Correlation Coefficient was calculated using the provided equation. Additionally, visual comparisons were also conducted.

$$r = \frac{\sum(x_i - \bar{x})(y_i - \bar{y})}{\sqrt{\sum(x_i - \bar{x})^2 \sum(y_i - \bar{y})^2}}$$

Where r = Pearson's Correlation Coefficient, x = value of the first variable, $\bar{x}$ = mean value of the first variable, y = value of the second variable, and $\bar{y}$ = mean value of the second variable.

The Pearson's Correlation Coefficient is a critical metric used to assess the degree of similarity between the reference and simulated torque profiles. It serves as an indicator of the suitability of the direct drive joint at its installed position. A low correlation coefficient value suggests an unsuitable joint, while a value closer to one indicates that the joint is well-suited for its installed position within the robot. The correlation coefficient is specifically calculated for the torque profile, as the correlation coefficients of the position and velocity profiles are dependent on the coefficient of the torque profile. Fig 12 demonstrates the response of an underpowered joint, highlighting the significance of adequate power for joint performance.

Fig 12: (a) Position, (b) Velocity, (c) Torque Reference vs Simulation Data of Joint 2 when an underpowered joint was installed at this location.

The underpowered joint resulted in significant deviations in position and velocity responses from the reference trajectory due to its inability to provide the required torque. The generated torque fell short, reaching around 50 Nm compared to the required 70 Nm, leading to a failure in following the trajectory. The correlation coefficient of 0.2593 quantitatively indicated a mismatch between the required and generated torque. To address this issue, we iterated the simulation using different integrated drive joints, gradually moving from low to high power joints. The selection process began with the last joint and moved backward, considering the impact of later joints on the earlier ones. This selection methodology aimed to identify optimal joints for

each position. Intelligent selection methods will be implemented as the library of simulated integrated drive joints expands, currently including frameless BLDC motors and strain wave gears from Maxon, TMotor, and Laifual Drive. Furthermore, we successfully resolved the underpowered joint issue by selecting suitable direct drive joints and tested the robot on a pick-and-place trajectory. Trajectories for position, velocity, and torque were obtained for all five joints of the 5-DOF robot. Through visual inspection, point-by-point comparison, and Pearson's correlation coefficient analysis, we confirmed that the joint trajectories of the simulated model closely followed the reference trajectories, surpassing the defined acceptable level of correlation coefficients greater than 0.95.

For the pick and place application, the reference versus simulated data for all the joints is shown in Figs 13 to 17.

Fig 13: Reference vs Simulation Data of Joint 1's (a) Position, (b) Velocity, and (c) Torque.

Fig 14: Reference vs Simulation Data of Joint 2's (a) Position, (b) Velocity, and (c) Torque.

Fig 15: Reference vs Simulation Data of Joint 3's (a) Position, (b) Velocity, and (c) Torque.

Fig 16: Reference vs Simulation Data of Joint 4's (a) Position, (b) Velocity, and (c) Torque.

Fig 17: Reference vs Simulation Data of Joint 5's (a) Position, (b) Velocity, and (c) Torque.

The simulation results demonstrate that the simulated integrated drive joint successfully produces the reference trajectory in all cases. The torque difference graph reveals a disparity between the required and simulated torque, but this difference does not result in noticeable deviations in the position or velocity trajectories due to the robustness of the PI controller. Additionally, Table 3 provides the Pearson's Correlation Coefficients for all joints, quantifying the degree of match between the reference and simulated trajectories.

Table 3: Pearson's Correlation Coefficient between Reference and Simulated Torque Profiles of all joints for Pick and Place Trajectory.

Parameter	Joint 1	Joint 2	Joint 3	Joint 4	Joint 5
Correlation Coefficient	0.9969	0.9984	0.9982	0.9992	0.9963

The high correlation coefficients obtained in the simulation experiments highlight the effectiveness of the simulation tool and the chosen motor and harmonic drive combinations in achieving accurate and reliable robot motion control. These results demonstrate that the

simulated robot performance closely aligns with the intended reference trajectories, which is crucial for ensuring precise and consistent robot motion.

Several sources of noise and disturbances including EMI, sensor (encoder) noise, input and output disturbances can cause the manipulator to deviate from an ideal trajectory in the real-life. We analyzed how adequately our PI controller would reject sources of noise disturbances in real-life by lumping these at the output of the speed controller, in the form of a single noise source. Specifically, this is an additive white gaussian noise with unit variance, resulting in a signal-to-noise ratio (SNR) of 15 dB, which is in close agreement with (Fig.2) noise levels observed in real experiments. Fig 18 depicts the responses of position, velocity, and torque tracking for one of the joints, joint 3, subject to noise/disturbance.

Fig 18: Reference vs Simulation data of Joint 3 after introducing disturbance, (a) Position, (b) Velocity, and (c) Torque.

In Fig 18, although there is distortion in the speed and, consequently, in the torque, the PI controller implemented in the position loop adeptly mitigates the effects of the introduced disturbance. This reinforces the robustness of the controller.

This finding carries significant implications for practical applications, as it suggests that physically manufactured robots using the same integrated drive joints and control system can potentially achieve similar performance. The subsequent section presents the results of fabricating the robot based on the integrated drive joints recommended by the simulation studies.

Section IV

Hardware Experimentation

As per the results obtained from the simulation experimentation, integrated drive joints were manufactured using the suggested commercially available components. Moreover, to meet the latest industrial standards, BLDC motor controllers from Nanotec® having EtherCAT communication capability were used to drive the BLDC motors. Five such joints were manufactured along with the manipulator's structure. Assembling all the subsystems produced a 5 DOF manipulator, shown in Fig 19, being controlled via EtherCAT enabled BLDC motor controllers.

Fig 19: Manufactured 5DOF Robot with Integrated Drive Joints performing a pick and place task.

We used Plug & Drive software, provided by Nanotec®, to tune the PI control loops of the motor controllers. The motor controller, just like our simulated motor controller, implements the PI control loops on three levels, that is the inner most on torque, then on velocity, and the outer

most on position. The position and velocity following response of one of the motor controllers before and after tuning is shown in Figs 20 and 21.

Figs 20 and 21: Before and After tuning response of position (left) and Velocity (right) of one of the joints. The x-axis shows time in milliseconds, whereas y-axis shows the position and velocity in motor controller units.

The physically developed robot was executed on the same trajectory used in the simulation, and the corresponding torque, velocity, and position data from the integrated drive joints were collected. The collected data was then compared with the simulated data for the pick and place trajectory, as shown in Figs 22 to 26. It is important to note that the time constant of a geared joint prevents any noticeable movement of the output shaft due to current spikes. However, for experimentation purposes, the torque data was obtained from the current sensor in the motor controller and passed through a moving average filter with a window size of 15 samples, approximately equal to 0.15 seconds in time. This moving average filter emulates the damping effect of the geared joint.

Fig 22: Simulated versus Experimental data of Joint 1's (a) Position, (b) Velocity, and (c) Torque.

Fig 23: Simulated versus Experimental data of Joint 2's (a) Position, (b) Velocity, and (c) Torque.

Fig 24: Simulated versus Experimental data of Joint 3's (a) Position, (b) Velocity, and (c) Torque.

Fig 25: Simulated versus Experimental data of Joint 4's (a) Position, (b) Velocity, and (c) Torque.

Fig 26: Simulated versus Experimental data of Joint 5's (a) Position, (b) Velocity, and (c) Torque.

Once again, visual and Pearson's correlation coefficient were used to establish the similarity of the trajectories coming from the simulated model and the actual robot. Table 4 lists the Pearson's Correlation Coefficient for torque profiles of all joints.

Table 4: Pearson's Correlation Coefficient between Simulated and Actual Torque Profiles of all joints for Pick and Place Trajectory.

Parameter	Joint 1	Joint 2	Joint 3	Joint 4	Joint 5
Correlation Coefficient	0.9966	0.9872	0.9882	0.9888	0.9895

Although the correlation coefficients have slightly decreased, they remain within an acceptable range as the position and velocity trajectories are still being accurately followed.

However, the mismatches in the torque trajectories require further investigation. The torque impulses observed in the physically manufactured robot are natural and result from current readings taken from the motor controller. These impulses are within the current limits of the BLDC motors and do not cause any damage or deviation in the required position trajectory. Additionally, the average torque deviation from the simulated model remains below 5 Nm for most of the time, which is a small percentage of the nominal torque capability of the integrated drive joints. This additional torque compensates for unmodeled dynamics, friction, and manufacturing variations. Simulation tools are useful for predicting robot behavior but cannot fully account for real-world complexities such as friction and manufacturing variations. However, the position and velocity following performance of the robot is still under acceptable level due to the disturbance rejection and noise cancellation properties of the PI controllers used. These results emphasize the importance of considering real-world complexities and the need for ongoing calibration and optimization. Further investigation will be conducted to minimize the impact of these mismatches in future work.

Furthermore, the repeatability of the developed 5-DOF robot prototype was calculated as per the procedure described in ISO 9283:1998 (32), defined specifically for industrial robots. Dial gauge having positional resolution of 0.01mm was used to measure the position of the end-effector of the robot. Five different positions were commanded to the robot and 100 iterations were carried out for each position to generate the position data. To calculate the positioning repeatability the following calculations were carried out.

$$l_i = \sqrt{(x_i - \bar{x})^2}$$

$$\bar{l} = \frac{1}{n}\sum_{i=1}^{n} l_i$$

Where ‘x_i’ is the measured value in i^th iteration, ‘$\bar{x}$’ is the mean position of all iterations, ‘n’ is the number of iterations, and ‘$\bar{l}$’ is then the mean positional repeatability according to ANSI/RIA R15.05 standard (33). Moreover, the standard deviation ‘S’ is then calculated as:

$$S = \sqrt{\sum_{i=1}^{n} \frac{(l_i - \bar{l})^2}{n-1}}$$

Lastly, the positional repeatability, ‘Rp’, is defined and calculated as

$$Rp = \bar{l} + 3S$$

Table 5 shows the Standard Deviation and the Positional Repeatability for each position. The worst value of the repeatability achieved in this experimentation is claimed as the repeatability of the developed robot prototype. Comparison with commercially available lightweight robots establishes that this value of repeatability is acceptable for robotic

manipulators. The worst case value of repeatability, 0.0503 mm, of our developed robotic manipulator is equivalent to commercially available lightweight robot Techman Robot TM5 (34).

Table 5: Standard Deviation and Repeatability of 5-DOF robot measured and calculated as suggested in ISO 9283:1998 for five different positions. All values are in mm.

Robot	Position 1		Position 2		Position 3		Position 4		Position 5	
	S	Rp	S	Rp	S	Rp	S	Rp	S	Rp
5-DOF	0.0018	0.0080	0.0039	0.0163	0.0062	0.0260	0.0107	0.0503	0.0030	0.0138

Overall, the developed simulation tool demonstrates its potential for robot design and optimization, particularly in the selection of integrated drive joint subcomponents. Using the proposed scheme, the authors along with their research team have also developed a 6-DOF robot manipulator, shown in Fig 27. However, it’s still in the testing phase. Fig 27 shows the configuration and hardware of this newly developed manipulator.

Fig 27: Newly developed 6 DOF Robot’s FBD (Left) and its hardware (Right).

Section V

Conclusions

In conclusion, this research emphasizes the advantages of adopting a component-driven approach in the design and fabrication of lightweight robotic manipulators. By developing models of commercially available components and integrating them into the design process, the gap between simulation and physical systems can be significantly reduced, leading to elimination or reduction of various steps involved in the robotic manipulator development process.

The presented framework demonstrates the effectiveness of this approach through the modeling and analysis of integrated drive joints in a 5-DOF lightweight robotic manipulator. Notably, the experimental evaluation of the developed robotic system yielded impressive results. The positional repeatability, assessed in accordance with ISO 9283:1998, demonstrated a repeatability of 0.05 mm, a performance level comparable to the Techman Robot TM5, a commercially available lightweight robot.

Subsequent hardware experimentation, where the same integrated drive joints and robot structure were manufactured and driven on predetermined trajectories, revealed that while position and velocity trajectories closely matched the simulated model, a discernible difference in torque requirements was observed. This disparity can be attributed to unmodeled dynamics, such as friction arising from strain wave gears and misalignments in the assembly process.

Nevertheless, the magnitude of the torque difference remained small compared to the nominal torque capability of the joints.

The proposed simulation tool and its framework hold significant potential in revolutionizing the robot manufacturing industry. By enabling developers working in educational research institutes, robotics startups, and other similar positions, to simulate robotic manipulators using models of actual subcomponents, it offers substantial time, effort, and investment savings in today's fast-paced and resource-intensive environment.

Future research endeavors will focus on further refining the simulation tool, expanding the library of simulated models to incorporate components from various manufacturers, and addressing the challenges posed by unmodeled dynamics. This ongoing development will continue to enhance the accuracy, reliability, and practicality of the proposed framework, further solidifying its potential as a game-changer in the field of robot manufacturing.

In conclusion, the component-driven approach presented in this research has demonstrated its effectiveness in facilitating the rapid development of lightweight robotic manipulators. By leveraging commercially available components and optimizing their integration through simulation and hardware experimentation, this framework offers valuable insights and practical solutions for the advancement of robotic systems.

Acknowledgements

This research is supported by the Higher Education Commission (HEC) Pakistan under the project ‘The establishment of National Center for Robotics and Automation (NCRA) with grant number NRPU-16242. We would also like to thank Dr. Ali Raza, Principal Investigator (PI) of Human Centered Robotics Lab UET Lahore for his unwavering support and guidance.

References

1. Akundi A, Euresti D, Luna S, Ankobiah W, Lopes A, Edinbarough I. State of Industry 5.0—Analysis and identification of current research trends. Applied System Innovation. 2022;5(1):27.
2. Hirzinger G, Albu-Schaffer A, Hahnle M, Schaefer I, Sporer N, editors. On a new generation of torque controlled light-weight robots. Proceedings 2001 ICRA IEEE International Conference on Robotics and Automation (Cat No 01CH37164); 2001: IEEE.
3. Popić S, Miloradović B. Light weight robot arms-an overview. INFOTEH-JAHORINA. 2015;14.
4. Balding S, Gning A, Cheng Y, Iqbal J. Information Rich Voxel Grid for Use in Heterogeneous Multi-Agent Robotics. Applied Sciences. 2023;13(8):5065.
5. Bilal M, Rizwan M, Maqbool HF, Ahsan M, Raza A. Design optimization of powered ankle prosthesis to reduce peak power requirement. Science Progress. 2022;105(3):00368504221117895.
6. Naeem A, Rizwan M, Maqbool HF, Ahsan M, Raza A, Abouhossein A, et al. Virtual constraint control of Knee-Ankle prosthesis using an improved estimate of the thigh phase-variable. Biomedical Signal Processing and Control. 2022;73:103366.
7. Calvo R, Gil P. Evaluation of collaborative robot sustainable integration in manufacturing assembly by using process time savings. Materials. 2022;15(2):611.

8. Gheorghe G, editor Concepts and mechatronics and cyber-mixmechatronics constructions, integrated in COBOT type technology platform for intelligent industry (4.0). Proceedings of the International Conference of Mechatronics and Cyber-MixMechatronics–2019 3; 2020: Springer.
9. Timms MJ. Letting artificial intelligence in education out of the box: educational cobots and smart classrooms. International Journal of Artificial Intelligence in Education. 2016;26:701-12.
10. Djuric AM, Urbanic R, Rickli J. A framework for collaborative robot (CoBot) integration in advanced manufacturing systems. SAE International Journal of Materials and Manufacturing. 2016;9(2):457-64.
11. Chung JC, Hwang T-S, Wu C-T, Jiang Y, Wang J-Y, Bai Y, et al. Framework for integrated mechanical design automation. Computer-aided design. 2000;32(5-6):355-65.
12. SANCHEZ S. Career Karma2022. Available from: https://careerkarma.com/blog/how-to-build-your-own-robot/#:~:text=How%20Long%20Does%20It%20Take,mechanical%20and%20electrical%20assembly%20expertise.
13. Ruishu Z, Chang Z, Weigang Z, editors. The status and development of industrial robots. IOP Conference Series: Materials Science and Engineering; 2018: IOP Publishing.
14. BRACJUNOVÁ VL. Robots take the robot out of the human: Soitron Group; 2019 [Available from: https://www.soitron.com/robots-take-the-robot-out-of-the-human/#:~:text=Creating%20a%20new%20robot%20from,part%20of%20the%20development%20process.
15. Hirose S, Arikawa K. Coupled and decoupled actuation of robotic mechanisms. Advanced Robotics. 2001;15(2):125-38.
16. Tarkian M, O¨ lvander J, Feng X, Petterson M, editors. Design automation of modular industrial robots. International Design Engineering Technical Conferences and Computers and Information in Engineering Conference; 2009.
17. Covell DM. Top-Down & Bottom-Up Approaches to Robot Design: West Virginia University; 2022.
18. Zimmermann M, Königs S, Niemeyer C, Fender J, Zeherbauer C, Vitale R, et al. On the design of large systems subject to uncertainty. Journal of Engineering Design. 2017;28(4):233-54.
19. Sathuluri A, Sureshbabu AV, Frank J, Amm M, Zimmermann M. Computational Systems Design of Low-Cost Lightweight Robots. Robotics. 2023;12(4):91.
20. Walden DD. Systems engineering handbook: A guide for system life cycle processes and activities. (No Title). 2015.
21. Albu‐Schäffer A, Haddadin S, Ott C, Stemmer A, Wimböck T, Hirzinger G. The DLR lightweight robot: design and control concepts for robots in human environments. Industrial Robot: an international journal. 2007.
22. Bolignari M, Fontana M. Design and experimental characterization of a high performance hydrostatic transmission for robot actuation. Meccanica. 2020;55(5):1169-79.
23. Mo S, Li X, Yang Z-n, Zhou C-p, Gao H-j, Cen G-j, et al. Integrated design of brushless motor drive and control system for robot joints. Journal of Central South University. 2021;28(12):3818-28.
24. Bischoff R, Kurth J, Schreiber G, Koeppe R, Albu-Schäffer A, Beyer A, et al., editors. The KUKA-DLR Lightweight Robot arm-a new reference platform for robotics research and manufacturing. ISR 2010 (41st international symposium on robotics) and ROBOTIK 2010 (6th German conference on robotics); 2010: VDE.
25. Mohanraj D, Aruldavid R, Verma R, Sathyasekar K, Barnawi AB, Chokkalingam B, et al. A Review of BLDC Motor: State of Art, Advanced Control Techniques, and Applications. IEEE Access. 2022.
26. Routh B, editor Design aspects of harmonic drive gear and performance improvement of its by problems identification: A review. AIP conference proceedings; 2018: AIP Publishing LLC.
27. Machado TDN, Sousa M, Monteiro S, Bicho E. CoopDynSim: a 3D robotics simulator. 2012.

28. Baizid K, Ćuković S, Iqbal J, Yousnadj A, Chellali R, Meddahi A, et al. IRoSim: Industrial Robotics Simulation Design Planning and Optimization platform based on CAD and knowledgeware technologies. Robotics and Computer-Integrated Manufacturing. 2016;42:121-34.
29. Bose BK. Modern power electronics and AC drives: Prentice hall Upper Saddle River, NJ; 2002.
30. Gutiérrez S, Zotovic R, Navarro M, Meseguer M. Design and manufacturing of a prototype of a lightweight robot arm. Procedia Manufacturing. 2017;13:283-90.
31. San-Miguel A, Puig V, Alenyà G. Disturbance observer-based LPV feedback control of a N-DoF robotic manipulator including compliance through gain shifting. Control Engineering Practice. 2021;115:104887.
32. Standardization TIOf. ISO 9283:1998(en) Manipulating industrial robots — Performance criteria and related test methods. 1998.
33. Institute ANS. American National Standard for Industrial Robots and Robot Systems - Point-to-Point and Static Performance Characteristics – Evaluation. ANSI/RIA R1505-1-1990 (R1999)1999.
34. TECHMANROBOTINC. TM5 - 900 2019 [Available from: https://www.tm-robot.com/en/tm5-900/.

Click here to access/download;Figure;Fig18.eps

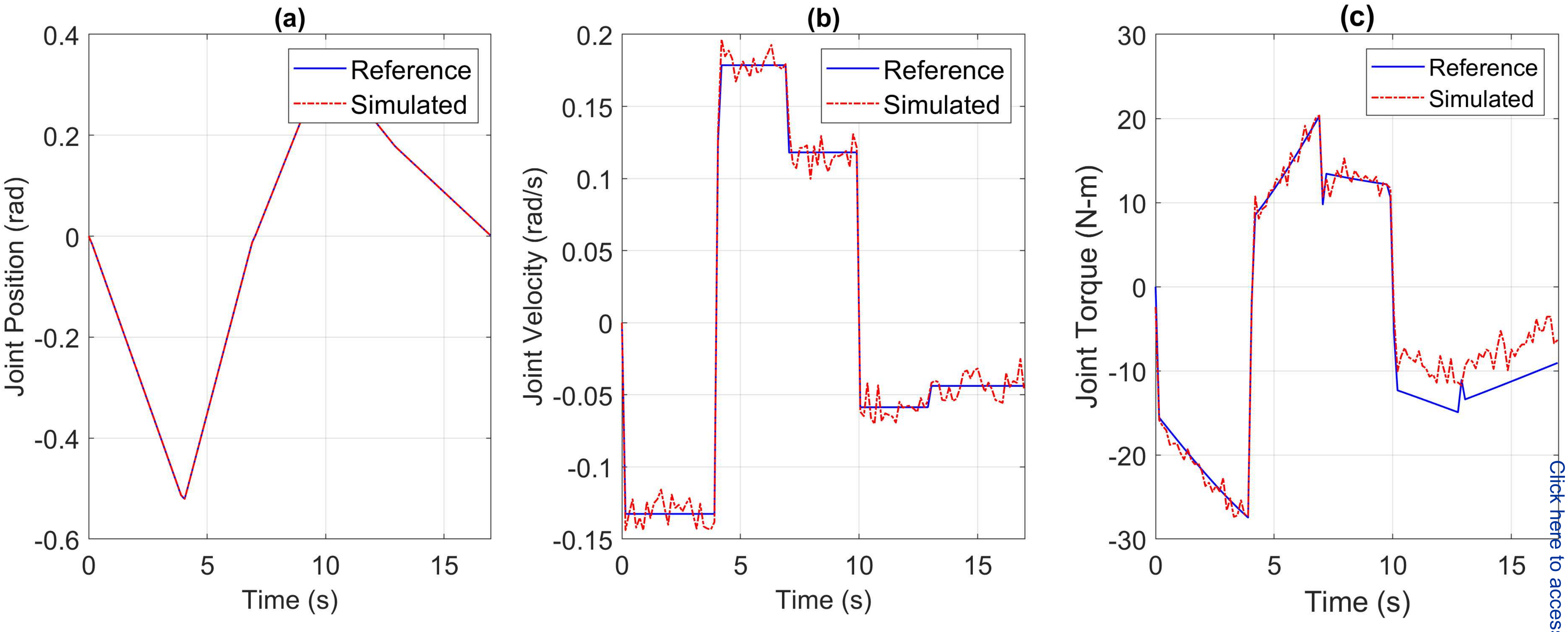

Click here to access/download;Figure;Fig19.eps

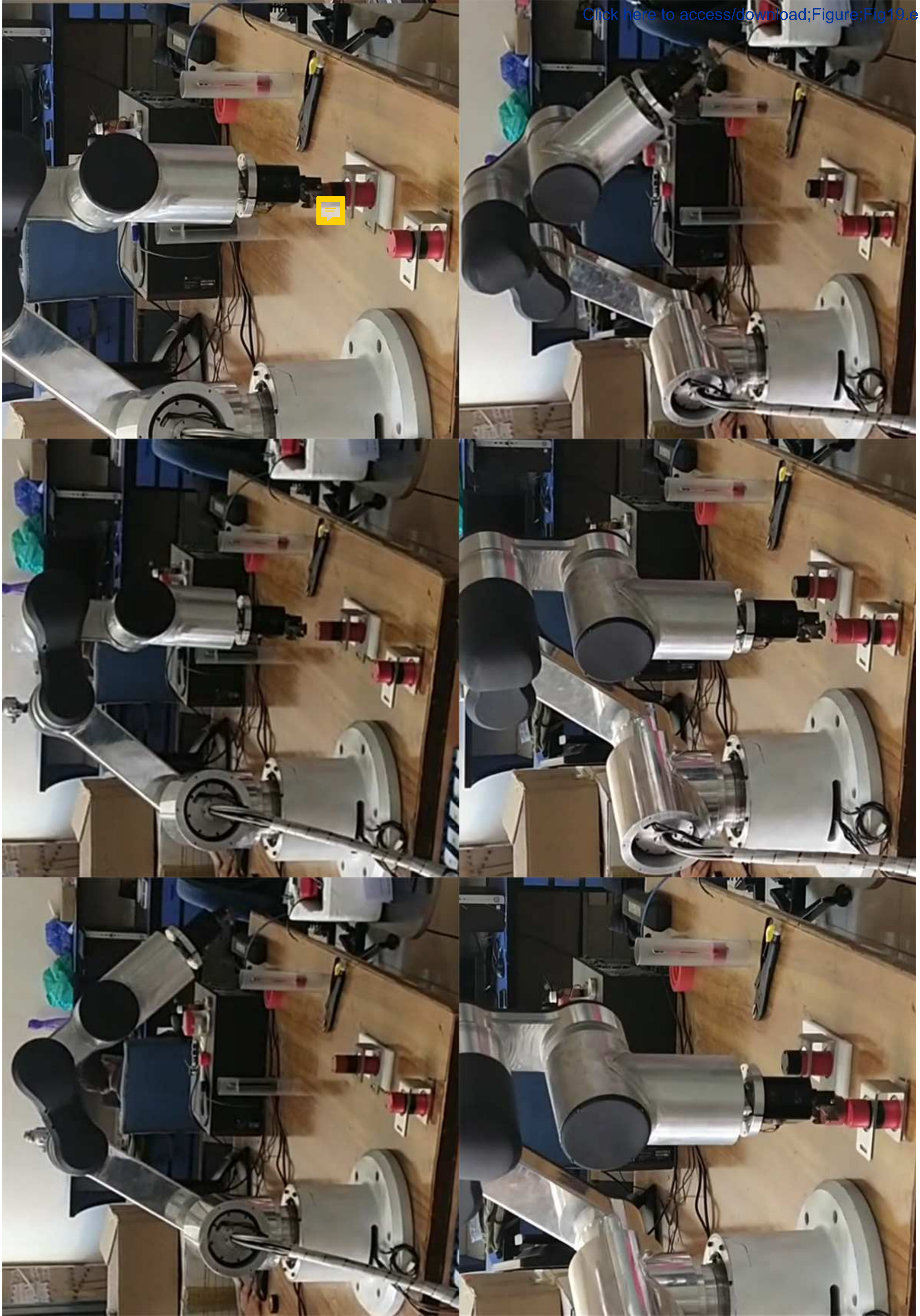

Click here to access/download;Figure;Fig20.eps

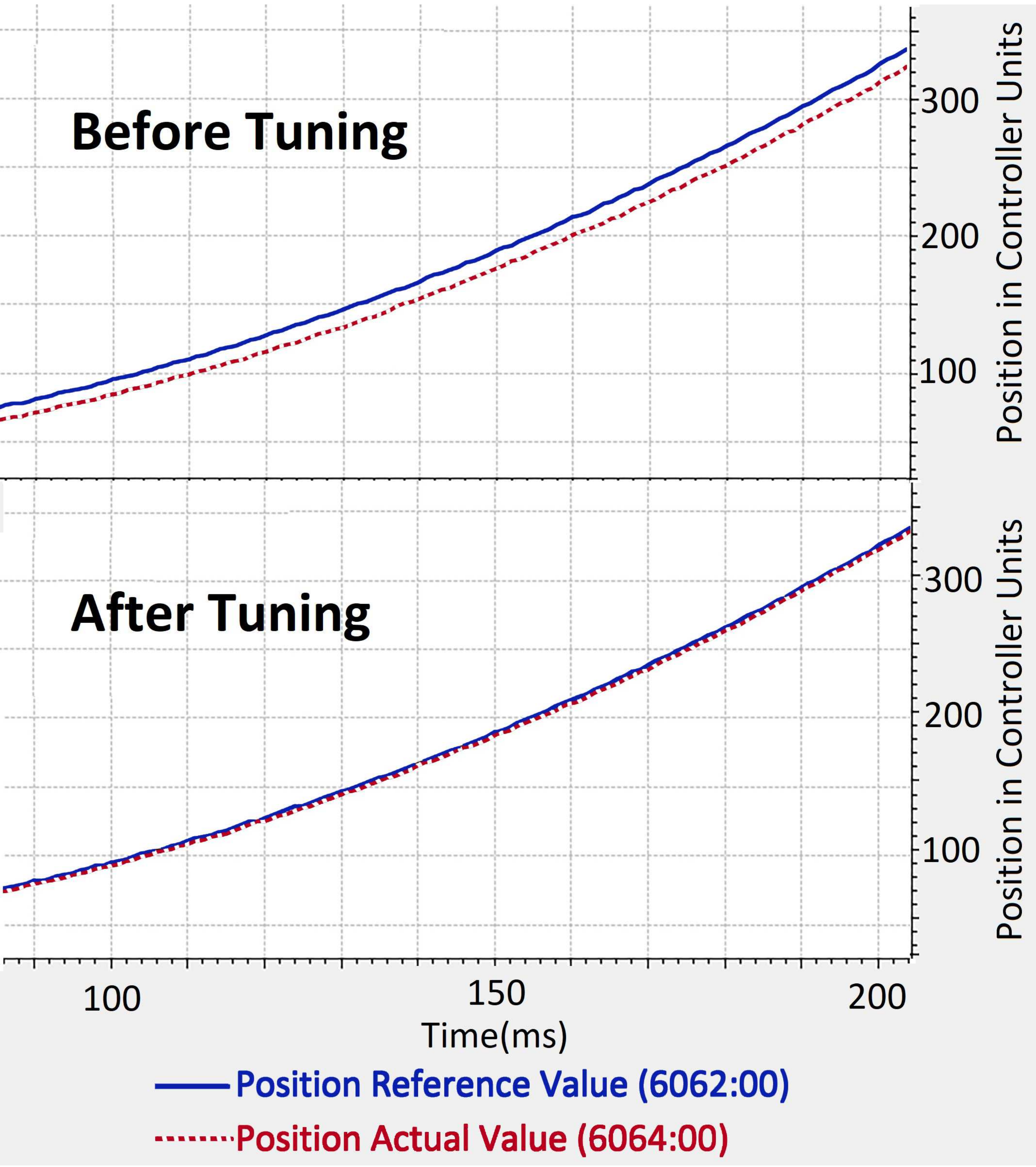

Click here to access/download;Figure;Fig21.eps

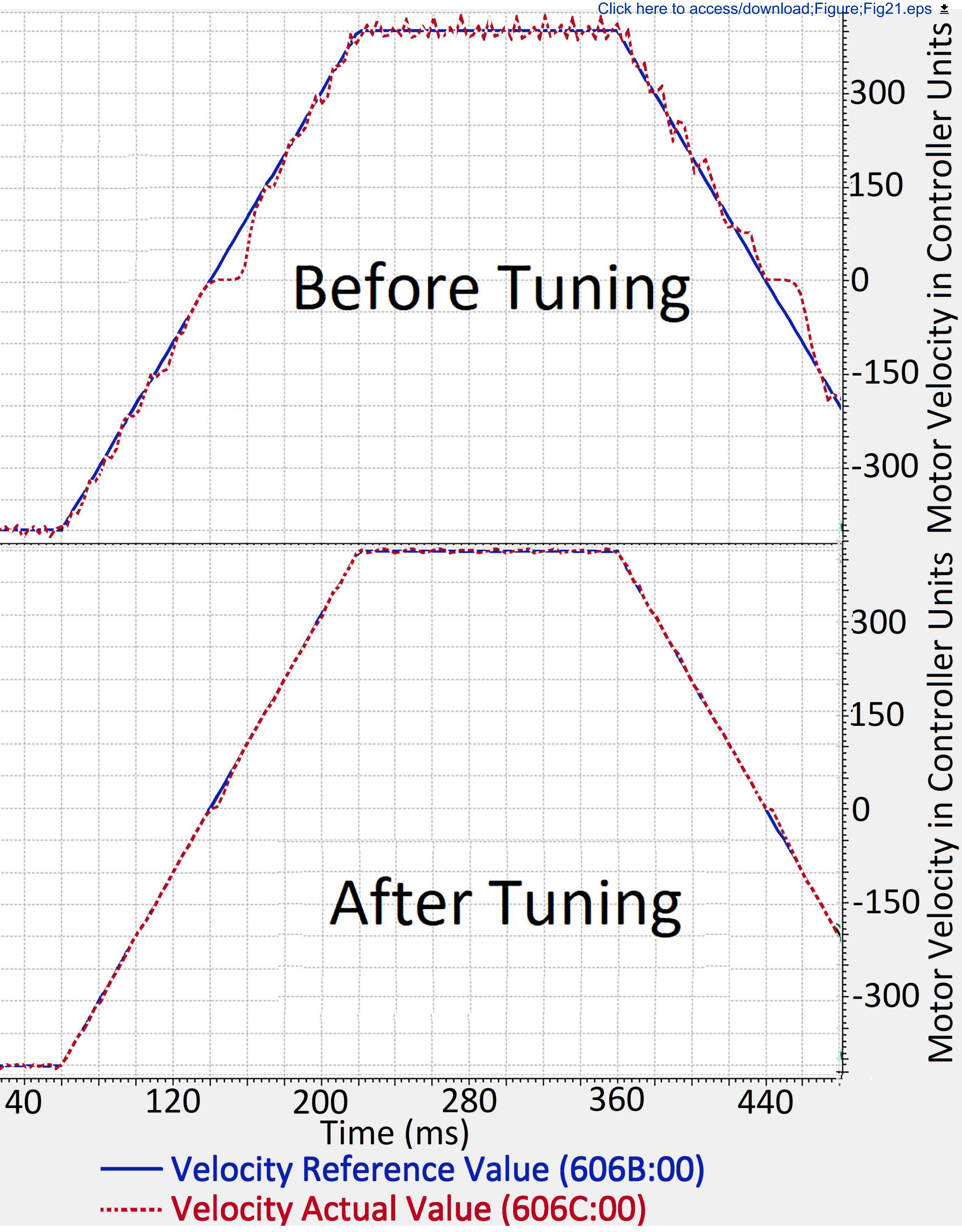

Click here to access/download;Figure;Fig22.eps

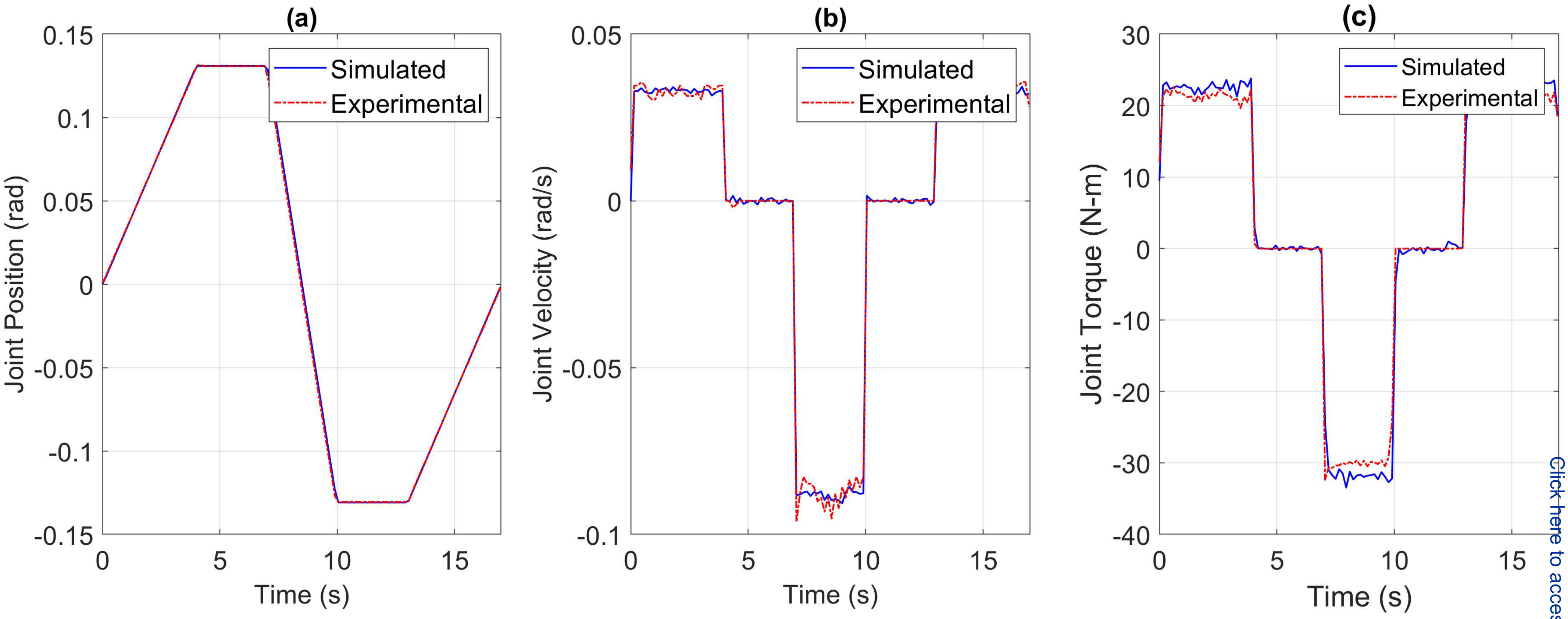

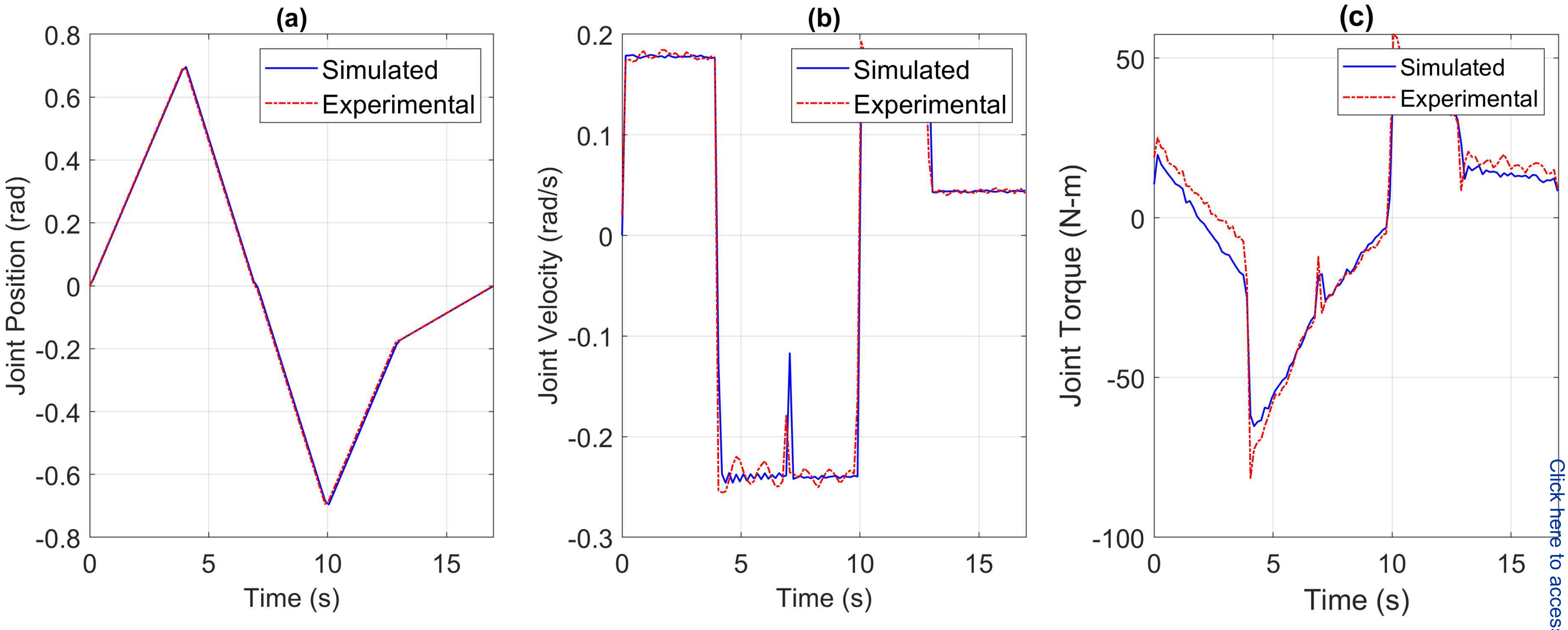

Click here to access/download;Figure;Fig23.eps

Click here to access/download;Figure;Fig24.eps

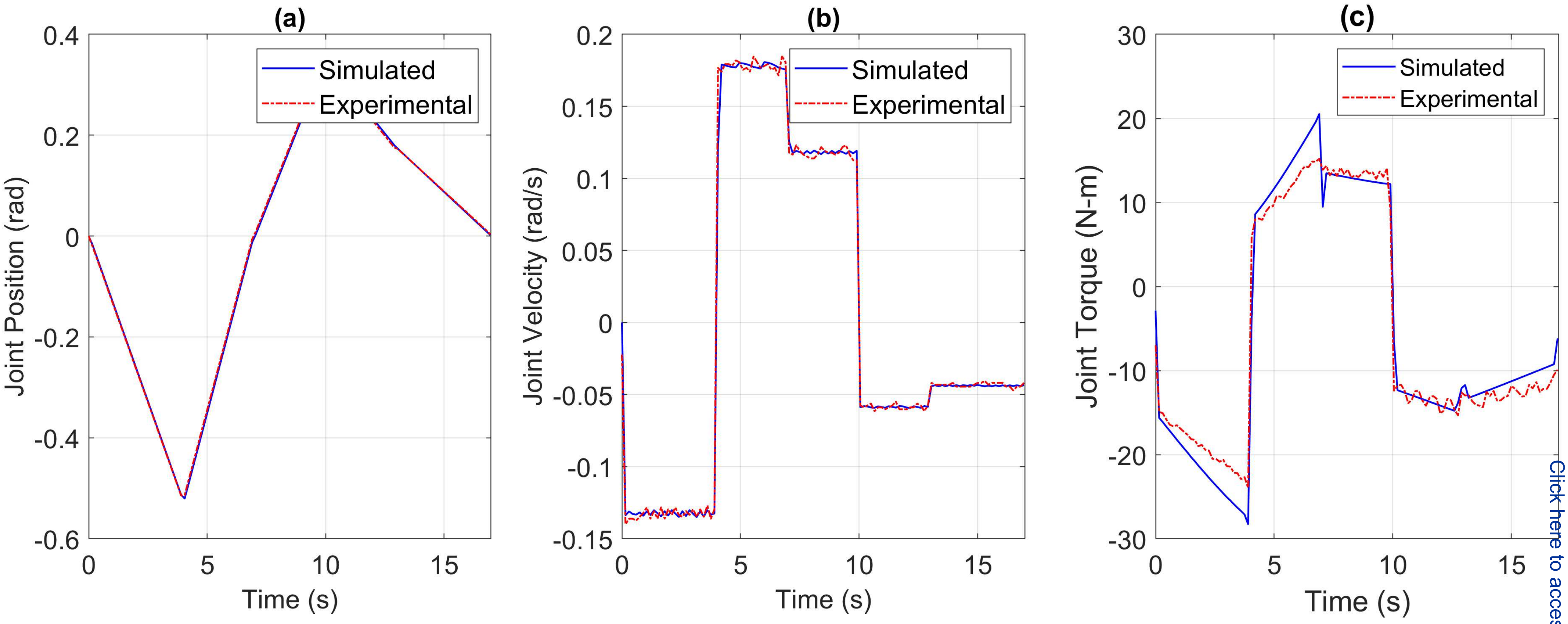

Click here to access/download;Figure;Fig25.eps

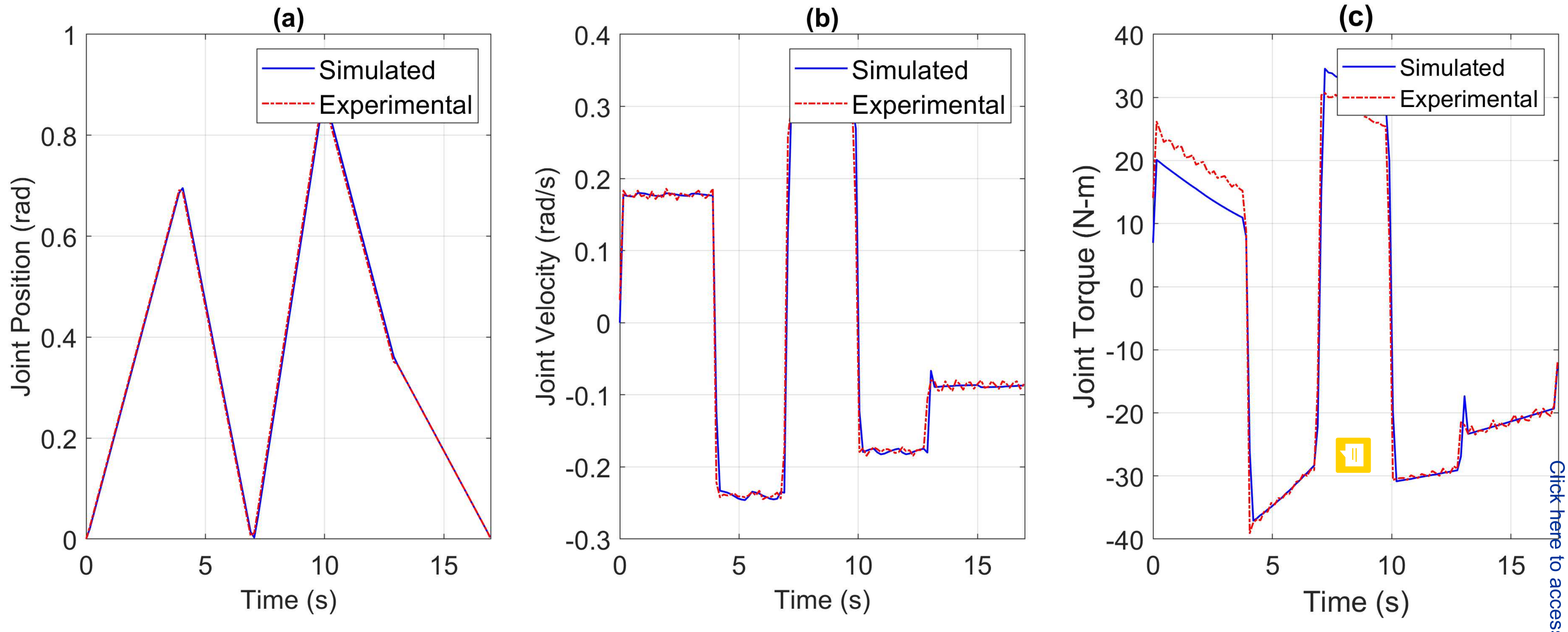

Click here to access/download;Figure;Fig26.eps

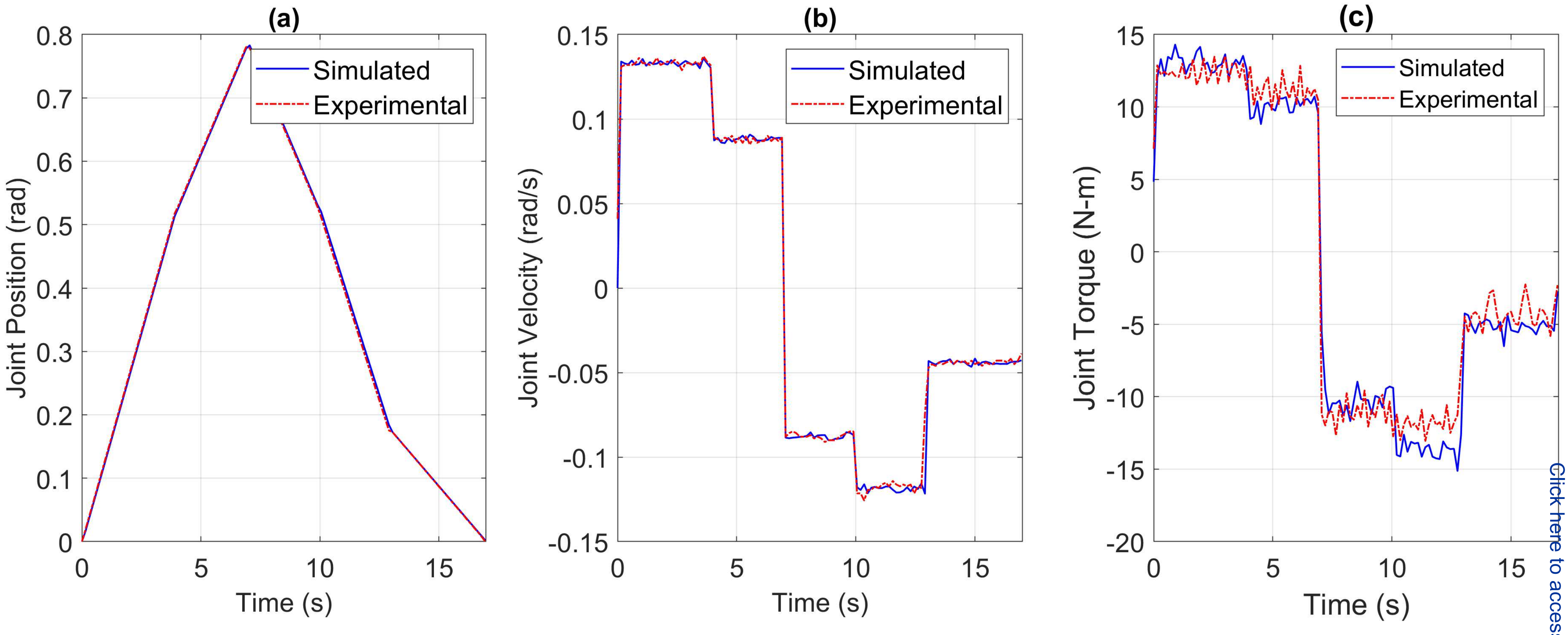

Click here to access/download;Figure;Fig27.eps

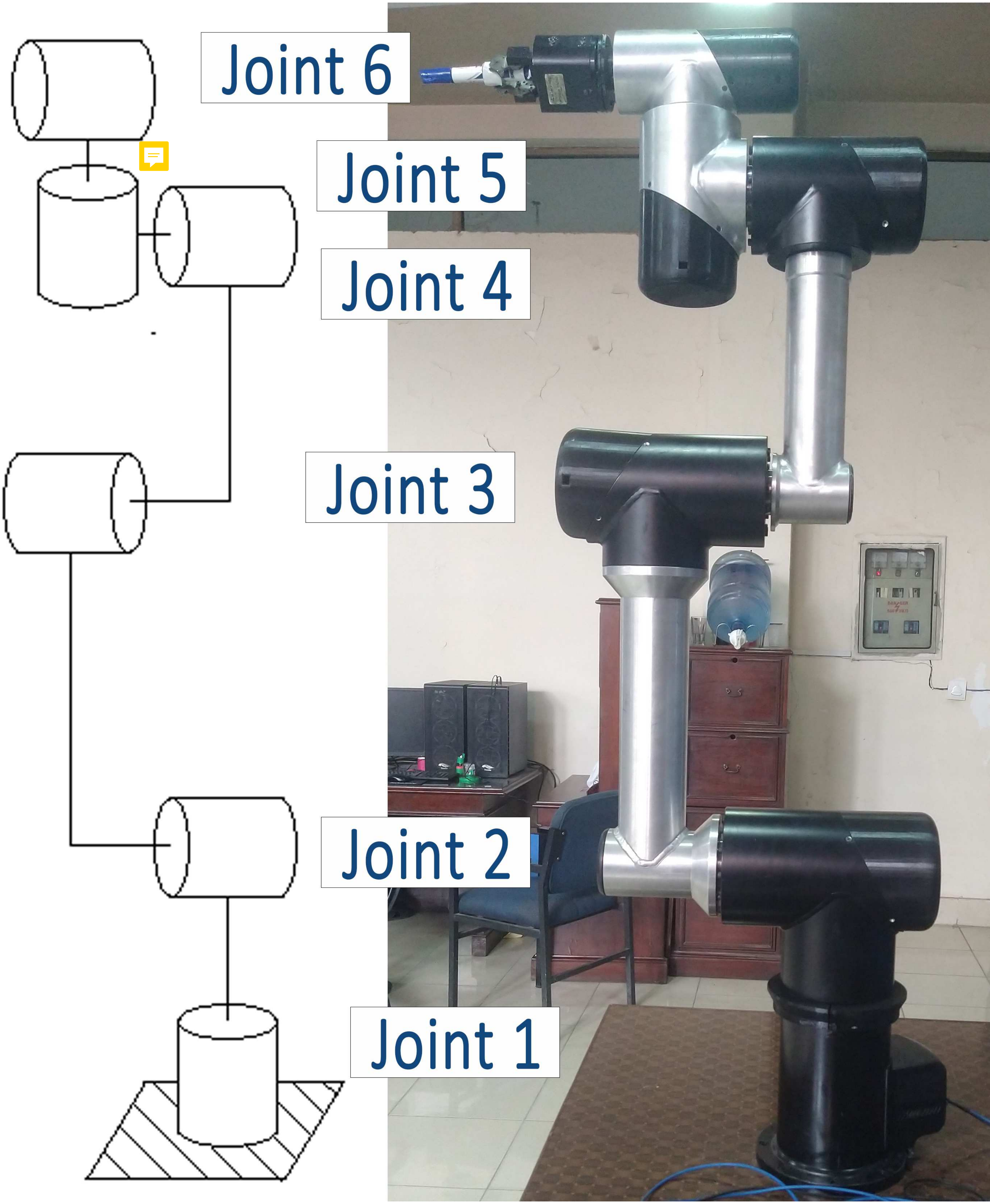

Experimental Development of Lightweight Manipulators with Improved Design Cycle Time that leverages off-the-shelf Robotic Arm Components

Formatted: Font: 18 pt

Muhammad Rzi Abbas[1], Muhammad Ahsan[1], Jamshed Iqbal[2]

[1]Department of Mechatronics and Control Engineering, University of Engineering ~~&~~and Technology, Lahore, ~~54890,~~ Pakistan

[2] School of Computer Science, Faculty of Science and Engineering, University of Hull, Hull, United Kingdom

Abstract

Formatted: Font: 18 pt

The growing market for lightweight robots inspires new use-cases, such as collaborative manipulators for human-centered automation. However, widespread adoption faces obstacles due to high R&D costs and longer design cycles, although rapid advances in mechatronic engineering have effectively narrowed the design space to affordable robot components, turning the development of lightweight robots into a component selection and integration challenge. Recognizing this transformation, we demonstrate a practical framework for designing lightweight industrial manipulators using a case-study of indigenously developed 5 Degrees-of-Freedom (DOF) cobot prototype. Our framework incorporates off-the-shelf sensors, actuators, gears, and links for Design for Manufacturing and Assembly (DFMA), along with complete virtual prototyping. The design cycle time is reduced by approximately 40% at the cost of cobot real-time performance deviating within 2.5% of the target metric. Our physical prototype, having repeatability of 0.05mm calculated as per the procedure defined in ISO 9283:1998, validates the cost-effective nature of the framework for creating lightweight manipulators, benefiting robotic startups, R&D organizations, and educational institutes without access to expensive in-house fabrication setups.

Formatted: Indent: First line: 0.5"

Keywords

Lightweight robot, Cobot, Robot Design Process

Section I

Formatted: Font: 18 pt

Introduction

The field of robotics has undergone a significant evolution, particularly with the emergence of lightweight robots, aligning seamlessly with the requisites of Industry 5.0 (1). This progression has paved the way for substantial advancements in collaborative robotics, where lightweight robots, often referred to as "cobots," are tailored for harmonious human-robot

Formatted: Indent: First line: 0.5"

interaction in applications ranging from industrial collaboration to medical-related scenarios (2-6). These versatile entities boast characteristics that position them as adaptable collaborators within cooperative work environments. Their intrinsic attributes, characterized by reduced speeds and inertias, make them particularly conducive for seamless interaction with human counterparts, leading to a broadening spectrum of applications across diverse domains. From assembly line installations (7) to medical setups (8), from education sector (9) to space endeavors (10)~~-~~, these robots have found their utility across varied industries. The escalating demand for automation positions lightweight robots as pivotal players across industrial sectors (1). In this evolving landscape, our research takes on heightened significance, strategically addressing the imperative for streamlined design and development of lightweight robots, a cornerstone in facilitating the transition toward Industry 5.0-aligned industrial and R&D ecosystems.

Conventional engineering product design cycle (11) is illustrated in Fig~~ure~~ 1 which is used to argue about robotic product development cycle. The whole process may be divided into seven major stages as labeled by circled digits in the figure.

Fig~~ure~~ 1: Typical Robot Design and Development Cycle. The table in the figure shows typically what percentage of total design cycle time (12-14) is spent on each stage. The portions enclosed in Red Boxes are either eliminated or simplified through the proposed scheme.

- Stage 1 is the initial stage where the concept of the robot is formulated, and its basic design is outlined. It involves defining the robot's purpose, capabilities, and basic architecture. Typically, 10%[1] (12-14) of the total design cycle time is spent in this stage.
- Stage 2 relates to the detailed design of the robot's components, such as mechanical, electrical, and software aspects. Planning for the manufacturing and assembly processes also occurs in this stage, and it takes around 20% of the total design cycle time.
- Stage 3 implements the initial design, usually in simulated environments, which is a quicker method, or sometimes a physical prototype is also developed, that normally takes longer. Physical prototype development consumes around 15% of the design cycle time.
- Stage 4 tests and iterates the prototype based on several performance parameters. Typically, 15% of the design cycle time is spent on this activity.
- Stage 5 starts once the design is optimized and tested. The final version of the robot design is prepared for manufacturing. This includes sourcing components, manufacturing, and assembly. Normally 20% of the total design cycle time is utilized in this stage.
- Stage 6 tests a validates the manufactured robot to make it deployment ready. This stage typically consumes 15% of the total design cycle time.

[1] These values are based on general industry knowledge and practices. The breakdown of time spent on different stages of robot development can vary widely depending on the specific project, organization, and goals. These percentages are meant to give a rough idea of how time may classically be allocated in a typical robot development process.

- Stage 7 is related to production of documentation, user manuals, training material and deployment of the robot in the intended environment. This stage lasts around 5% of the total design cycle time.

Considering the extensive range of commercially available components, it would be both economical and timesaving for developers to simulate robotic manipulators exclusively composed of such components. The design approach presented in this paper modifies the robot development process along these lines, hence reducing the complexity and required time. First, stage 1 is reduced to only gathering of the robot specifications. The time needed to develop initial conceptual design has been reduced because of the developed library of components. Second, the modeling of the subcomponents allows for virtual prototyping, eliminating the need for physical prototype development, saving around 20% of the total design cycle time. Moreover, the developed library eliminates the requirement of subcomponents' performance analysis, further saving valuable development time. Lastly, the testing and validation of the manufactured robot is eliminated because the virtual prototype is made of all commercially available or produced components. The Pearson's Correlation Coefficient, presented in the experimentation section of this paper, between the performance of the virtual prototype and the developed robot confirms this claim.

This research work focuses on the simulation of hollow shaft Integrated Drive Joints (IDJs) integrated into lightweight robotic manipulators designed to operate as cobots. The developed library encompasses models of several commercially available subcomponents. Within the simulation environment, the IDJ models and the manipulator's structure are utilized to identify the optimal components for the integrated drive joints. Moreover, the simulation of the complete structure enables the consideration of coupled dynamics throughout the entire system, which is significant to control system engineers. While a single IDJ exhibits distinct performance characteristics, coupling multiple joints introduces coupled dynamic effects across all joints (15, 16). These couplings can be mitigated at the structural level through the appropriate selection of gearing systems or addressed at the software level by fine-tuning the control system accordingly.

The simulation tool was developed using the MATLAB/Simulink R2021b environment, while the robot structure was modeled using SolidWorks 2021. Subsequently, based on the findings obtained from the simulation tool, a 5 degrees-of-freedom (DOF) lightweight robot was developed, as depicted in Figure 2. Though the authors have also developed a 6-DOF lightweight robot by following the same footsteps, but for the sake of compactness of the article, the detailed analysis of 5-DOF robot is presented.

Figure 2: 5 DOF Robot's FBD (Left) and 5 DOF robot's hardware (Right).

The organization of the remaining manuscript is as follows: Section II describes the challenges involved in the in-house development of light-weight robots and then propose a suitable methodology for generating an IDJ model that includes a synergetic combination of

Formatted: Indent: First line: 0.5"

Formatted: Indent: First line: 0.5"

frameless brushless DC (BLDC) motor, strain wave gear and other subcomponents. Section III showcases the simulation results of the developed model, whereas Section IV details the performance results of the physically developed 5 DOF manipulator based on the in-horse manufactured integrated drive joint using the same hardware subcomponents as those used in the simulation. Lastly, Section V concludes the manuscript and steers the reader to possible avenues of future work.

Section II

Addressing challenges involved in the in-house development of Light-Weight Robots

In the current landscape of robot design methodologies, an extensive exploration of both bottom-up and top-down approaches is available in the literature. However, a notable gap persists as many of these analyses overlook the integration of commercially available, off-the-shelf components into the design process. While bottom-up strategies are apt for the development of generic multi-purpose robots, top-down approaches excel in crafting application-specific robotic solutions (17). The top-down paradigm initiates the design process with a clear focus on the final application, resulting in a more streamlined design space. Addressing this, recent studies (18) highlight the efficacy of top-down approaches in mitigating cyclic dependencies, particularly beneficial for the efficient design of mechatronic systems characterized by intricate interdependencies. Another pertinent contribution in this domain comes from a study on the optimization of reconfigurable collaborative robots, emphasizing modularity and structural considerations (19). This innovative top-down approach draws inspiration from the V-model employed in the automotive and aerospace industries, as introduced in (20). The optimization strategy revolves around enhancing the cost-effectiveness of development by optimizing the physical structure, consequently yielding savings in manufacturing materials. Additionally, a significant reduction in development time is achieved by capitalizing on established modular interfaces instead of customizing interfaces for each module, as detailed in (19). These contemporary studies collectively underscore the evolving landscape of design processes, shedding light on the nuanced benefits of incorporating off-the-shelf components within a top-down framework.

Like any robotic system, lightweight manipulators adhere to basic design principles and employ specific components to achieve their lightweight characteristics (21). The IDJ or Direct Drive Joint (DDJ) incorporates frameless BLDC Motors and Strain Wave Gears, distinct from conventional manipulators. Additionally, lightweight manipulators utilize differently designed links made from lightweight materials. Over the past two decades, researchers have proposed various drive unit designs suitable for lightweight robots (21-24). The design constraints imposed by the hollow shafts of drive units and of links connecting the drive units to form the kinematic chain, require specific component choices. For example, the flexspline of the strain wave gear and the lightweight structure of the robot introduce mechanical flexibility, necessitating the consideration of dynamic coupling among multiple drive units through lightweight links. Consequently, optimizing the subcomponents of drive units and links becomes a primary objective in the design and manufacturing of lightweight robots. Moreover, considering the

Formatted: Font: 18 pt

Formatted: Font: 11 pt

Formatted: Indent: First line: 0.5"

extensive experience in the development of robotic manipulator components (25, 26), it is prudent to leverage this existing knowledge to narrow down the design space.

To evaluate the performance of interconnected systems that incorporate multiple drive units and links, simulation tools are essential. However, the lack of simulation tools capable of simulating complete robotic systems with integrated drive joints forces developers to physically test and validate certain components. The discrepancy arises because designers often choose to simulate idealized systems or systems based on various assumptions in existing robot simulators (27, 28). There is a need to model complex systems using available hardware. For instance, when determining suitable gearing systems for a specific speed reduction task, a design engineer typically suggests gear specifications through calculations and simulations. However, these specific gearing systems might not be procurable or manufacturable. Conversely, it would be advantageous to explore the available gearing systems suitable for lightweight robotic manipulators, model them, and adapt other parts of the system to achieve the desired speed reduction. This concept extends beyond gearing systems to encompass motors, sensors, bearings, motor drives, and the robot structure as well.

In the realm of industrial robotics, decades of design and manufacturing experience have allowed for the identification of off-the-shelf components that can be utilized to construct high-performance functional manipulators. Adopting a counter approach, where the available components shape the design space, would be highly desirable if comprehensive simulated models of all commercially available components were readily accessible. Regrettably, the current landscape does not provide an extensive repertoire of simulated models, and even when a few such models do exist, there is a notable absence of a *unified and scalable platform* capable of testing the combinations of different components employed in the fabrication of critical elements, such as a single joint within a robotic manipulator or the manipulator as a complete system.

Figure 3 illustrates the comprehensive workflow of the proposed scheme. The end-user provides the system requirements, which serve as the basis for component selection, leading to the creation of a Computer Aided Design (CAD) model of the robot. This CAD model is subsequently imported into the Simulink environment, where it is subjected to predefined trajectories. Based on the evaluation of position, velocity, and torque tracking performance, frameless BLDC motors and/or strain wave gears may be substituted, if necessary. Once satisfactory performance is achieved in the simulation, the robot is fabricated and assembled utilizing the commercially available components identified during the simulation phase. To complete the entire process, a comparative analysis is conducted, contrasting the performance of the manufactured robot with that of the simulated robot.

Figure 3: Comprehensive workflow of the proposed scheme.

Methodology for Implementation of the Proposed Scheme

Formatted: Font: 18 pt

Formatted: Indent: First line: 0.5"

Developing a comprehensive simulation model that encompassesing all components of a real-life IDJ, presents a complex undertaking. However, it becomes evident that the frameless BLDC motor and the gearing system are the two major components. Consequently, detailed models were constructed for these components, while simplified approximations were employed for smaller subcomponents like position encoders and bearings. This approach is logical as the inertia of the IDJ is primarily attributed to the BLDC motor and the gearing system, with the BLDC motor also consuming most of the electrical power. Thus, both the electrical and mechanical characteristics of the IDJ are primarily dictated by these two key components.

The development of models for the involved subcomponents necessitates consideration of several aspects. For instance, a motor requires modeling of both its electrical and mechanical attributes, whereas a gearing system pertains solely to mechanical aspects. However, different types of gearing systems exhibit distinct behaviors. For example, a planetary gear system lacks flexibility within its internal gears but possesses measurable backlash. On the other hand, a strain wave gear introduces flexibility due to its flex spline but does not exhibit backlash. Backlash and rigidity are just a few of the differing characteristics between these two types of gearing systems. Therefore, a comprehensive modeling of a gearing system requires the consideration and optimization of various parameters based on the specific requirements. However, to streamline the process, it is practical to focus solely on modeling the parameters of commercially available hardware components, bypassing the optimization step entirely. Consequently, developers can expedite the process by leveraging existing hardware components and utilizing their models within the simulation environment, effectively bridging the gap between simulation and actual hardware.

The subsequent discussion elaborates on the potential components that an IDJ may encompass. However, it is worth noting that certain components may be omitted in practice without compromising performance, albeit at the expense of non-performance-related features. Moreover, the simulated models utilize parameters sourced from component datasheets, which are typically freely available on the manufacturer's website.

Frameless BLDC Motor Model

Formatted: Font: 16 pt

Formatted: Indent: First line: 0.5"

Extensive research has been conducted on developing simulation models for various types of motors, including BLDC motors. Given the objective of this study to establish an effective combination of motor models, strain wave gears, and approximate other components and parameters, there is no need to reinvent the wheel. Hence, standard BLDC motor models have been utilized and adapted to create models for commercially available frameless BLDC motors.

In addition to the BLDC motor, the drive system used to operate it plays a crucial role. The choice of drive system determines the required feedback and the level of control it can exert over the motor's motion. Typically, commercially available BLDC motor drives implement speed

control through stator current control, employing a position sensor such as a rotary encoder or a hall sensor. The generalized mechanical system dynamics are represented by:

$$T_e = J\frac{d}{dt}\omega_r + F\omega_r + T_m$$

Where T_e represents the electromagnetic torque, J the inertia, ω_r the rotor speed, F the viscous friction, and T_m the load torque. These kinds of drives have an inner current loop that will take in the commanded torque and utilize the torque constant of the motor to generate suitable stator phase currents. Whereas the outer speed control loop takes in the load or applied torque and reference speed to figure out the electromagnetic torque as per the mechanical system's dynamic equation. Furthermore, commercial motor drives implement Proportional-Integral (PI), Proportional-Derivative (PD) or Proportional-Integral-Derivative (PID) controller for maintaining the desired speed-torque characteristics. These controllers are pre-tuned or may easily be autotuned using the damping factor and the desired response time. Fig~~ure~~ 4 shows the block diagram of the overall control scheme for commercially available motor drives.

Fig~~ure~~ 4: General control scheme implementing closed loop control at all three stages, that is, torque (current), velocity, and position.

In this study, BLDC motor controllers incorporating PI controllers at all three levels were employed~~ at all three levels~~: the innermost current loop, velocity loop, and outermost position loop. The inclusion of an integrator in servomechanisms ensures zero steady-state error and helps to mitigate noisy signals commonly encountered in lightweight robotic manipulators, where weak sensor signals are in close proximity to high-powered BLDC motors. Moreover, the choice of using PI controllers only is further supported by the fact that all different types of hardware motor controllers used in this study employ only PI controllers to control the BLDC motor. The PI controllers of the hardware motor controllers were tuned according to the manufacturer's specifications, while the simulation-based motor controllers were autotuned using the damping factor and desired response time, as outlined in (29). The damping factor is defined as follows:

Formatted: Indent: First line: 0.5"

$$\zeta = \frac{\omega_n K_p}{2K_i}$$

If $\zeta < 0.69$, then natural frequency is defined as:

$$\omega_n = \frac{-1}{\zeta \times T_{nl}}\log(0.05 \times \sqrt{1-\zeta^2})$$

Where if $\zeta \geq 0.69$, then natural frequency is defined as:

$$\omega_n = \frac{0.9257}{T_{nl}}e^{1.6341\times\zeta}$$

Strain Wave Gear Model

In this research, the modeling of strain wave gears encompasses several crucial parameters that can be sourced from either the manufacturer's datasheet or the CAD model. These include weight, moment and product of inertia, stiffness, damping constant, nominal torque, and nominal speed. These parameters form the foundational framework for the accurate representation of strain wave gears within the simulation environment, ensuring a comprehensive and precise analysis of their performance characteristics.

While most parameters can be directly extracted, the moment and product of inertia are obtained from the manufacturer-provided CAD model. The damping constant is influenced not only by the grease used in the gearing system but also by the friction in the bearings and overall component alignment. Therefore, an empirical determination of the damping constant was performed for one of the manufactured joints to account for manufacturing imperfections and friction. Standardizing the manufacturing process, materials, assembly processes, strain wave gear grease, and bearings will improve the approximation of the damping constant. The determined damping constant was incorporated into the simulated model to capture its effects.

Robot Structure

For modeling the structure of the robotic manipulator, SolidWorks 2021 was employed in this research. All necessary parts were modeled, and analysis was performed to establish the validity. IDJ was modeled using the supplier-provided CAD models of the included components, which were the frameless BLDC motor, strain wave gear, optical encoder, bearings, and joint housing that will encase all the components. Moreover, the links and the connection flanges were modeled and passed through stress-strain analysis for establishing the validity of the model subjected to the worst-case loading. For links and joint housings, we used Aluminum 6061-T6 material because of its strength and lightweight, while for connection flanges we used Alloy Steel having yield strength of $6.2\text{x}10^8$ N/m^2. The factor of safety for Aluminum links is 2.4, while for flanges its 4.3. Figure 5 shows stress analysis figure of one of the links being subjected to a maximum of $1.158\text{x}10^8$ N/m^2 stress, while Aluminum 6061-T6 has yield strength of $2.75\text{x}10^8$ N/m^2, hence achieving factor of safety of 2.4. Some covers which were not load bearing were later 3D printed, hence doesn't need any analysis.

Figure 5: Stress Analysis of one of the links showing maximum stress being applied on it in worst case.

The CAD model of the robot's structure generated in SolidWorks was then imported into MATLAB's environment for simulation. The CAD model considered the following parameters:

- Robot configuration
- Link weights
- Link moment and product of inertias

Formatted: Font: 16 pt

Formatted: Indent: First line: 0.5"

Formatted: Font: 16 pt

Formatted: Indent: First line: 0.5"

Formatted: Indent: First line: 0.5"

- Link lengths

Fig~~ure~~ 6 illustrates one of the designed links and its integration with the joints to form the complete CAD model of the 5 DOF robot.

Fig~~ure~~ 6: Exploded view of the 5 DOF robot showing Links, Joints, Connection flanges, and 3D printed covers.

The robotic manipulator consists of additional subcomponents within the integrated drive joint that have smaller weight and moment of inertia. These components, listed below, can be grouped together, and represented as a single model by combining their net weights and inertias, as their individual effects on the dynamics are relatively minor.

Other components

In addition to the previously mentioned components, there are various other components that may or may not be included in an integrated drive joint (30). These components, such as the miniaturized circular circuit board, position sensor, bearings, torque sensor, brake, and housing, have significantly lower weights and moment of inertias compared to the BLDC motor, strain wave gear, and robot's structure. For analysis, these components are combined into a single unit for simplicity. The forthcoming section elaborates on the aspects considered for modeling and subsequent manufacturing of the integrated drive joint.

Simulation Model Flow

The direct drive joint model was developed by individually modeling and combining commercially available subcomponents. The model includes a frameless BLDC motor, strain wave gear, incremental position encoder, hollow shaft, bearings, joint housing, and miscellaneous small parts. The electrical aspects of the frameless BLDC motor were modeled in Simulink MATLAB using the Brushless DC Motor Drive (AC7) block, which represents a standard current-controlled drive for BLDC motors with closed-loop speed control. The mechanical aspects of the motor and strain wave gears were modeled in SolidWorks. Table 1 presents the parameters used to model the frameless BLDC motor, extracted from commercially available datasheets. The developed simulator includes eight different frameless BLDC motors from T-Motor and Maxon, allowing for modeling of various motors without manufacturer dependence.

Table 1: List of BLDC Motor Parameters that were used for modeling the commercially available motors. Sample of three different BLDC Motors are shown.

Sr. #	Parameter	Unit	TMotor RI50	TMotor RI80	Maxon 588849
1	Resistance	Ohm	0.673	0.196	0.109
2	Inductance	mH	0.474	0.364	0.0665
3	BEMF Flat Area	~~Deg.~~rad	~~68.6~~1.197	~~68.6~~1.197	~~43.6~~0.761
4	Torque Constant	mNm/A	99	127.3	80.7
5	Speed Constant	~~Rpm~~rad/s/V	~~96.5~~10.11	~~757~~.85	~~118~~12.36

Formatted: Indent: First line: 0.5"

Formatted: Font: 16 pt

Formatted: Indent: First line: 0.5"

Formatted: Font: 16 pt

Formatted: Indent: First line: 0.5"

6	Robot Inertia	$g.cm^2$	3500	6000	5300
7	Poles	-	7	7	11
8	Nominal Torque	mNm	500	1450	1010

Similarly, several parameters of strain wave gear were used to model its effect in the direct drive joint, out of which the gear ratio is defined in the motor block. Apart from gear ratio, the weight, moment and product of inertias, stiffness, nominal torque, and nominal speed parameters were defined and used in different blocks. Furthermore, the simulated direct drive joint model includes the weights and inertias of position encoder, bearings, joint housing, and the hollow shaft through which the power and communication wires are routed.

Formatted: Indent: First line: 0.5"

Figure 7 shows the section view of the CAD model of the simulated direct drive joint.

Figure 7: Section view of the CAD model of the assembled Integrated Drive Joint

Later, the simulated integrated drive joint was installed in the simulated robot structure, and the manipulator was driven on a defined trajectory. The position, velocity, and torque requirement graphs were plotted to establish the suitability of the simulated direct drive motor joint.

Formatted: Indent: First line: 0.5"

Manipulator Model

Formatted: Font: 16 pt

To evaluate the simulated integrated drive joint, it was integrated into a 5-DOF robotic manipulator model. This manipulator model was meticulously constructed in SolidWorks and seamlessly imported into MATLAB's Multibody environment. The development of the manipulator's model considered critical parameters, including robot configuration, link weights, link moment, product of inertias, and link lengths. These considerations were pivotal in ensuring the accuracy and effectiveness of the simulated manipulator's performance under various conditions.

Formatted: Indent: First line: 0.5"

Table 2 presents the parameters of the robot links and joints used to generate the simulation model. These parameters were obtained from the CAD model, which considers the commercially available material, its properties, and the machining processes involved. While importing the model from SolidWorks 2021 to the Simulink environment, the dimensions and shape of the links were preserved. However, the shape and size of the joints were approximated by a point mass while maintaining all other parameters.

Table 2: List of Parameters used for modeling the structure of the robot in Simulink Environment.

Sr. #	Manipulator Link		Manipulator Joint	
	**Parameter**	**Unit**	**Parameter**	**Unit**
1	Size and Shape	mm	Point Mass	-
1	Mass	kg	Mass	kg
2	Center of Mass	mm	Center of Mass	mm

3	Moments of Inertia	$g.cm^2$	Combined Moments of Inertia	$g.cm^2$
4	Products of Inertia	$g.cm^2$	Combined Products of Inertia	$g.cm^2$

Individual links of the robotic arm were imported into the Multibody environment and were assembled in the defined configuration to build the complete structure. Fig~~ure~~ 8 shows the Simulink model for building the robotic arm in MATLAB.

Fig~~ure~~ 8: Simulink SimScape Multibody model for building 5 DOF Robotic Arm

Section III

Formatted: Font: 18 pt

Simulation Experimentation

In the experimentation phase, direct drive joints featuring various commercially available frameless BLDC motors were integrated with the 5-DOF manipulator model. Trapezoidal velocity trajectories were generated as reference trajectories for different scenarios, and the simulated model was driven on these trajectories to evaluate the performance of the direct drive joints. Fig~~ure~~ 9 provides a snapshot of the simulation running on a specific trajectory.

Formatted: Indent: First line: 0.5"

Fig~~ure~~ 9: Snapshot of simulation of 5 DOF manipulator on a given trajectory.

To account for potential manufacturing inaccuracies, up to 10% random variations were introduced in the weight, inertia, and stiffness parameters of the simulated direct drive joint and robot's structure. These variations compensate for possible deviations that may arise during the actual manufacturing process, such as material and machining differences and assembly imperfections. Additionally, an empirically determined damping constant was incorporated to model frictional forces. A test joint was manufactured to evaluate manufacturing imperfections and determine frictional losses, which were then included in the simulated model. All joints of the 5-DOF manipulator were configured with a damping constant of 2 Nm/deg/s. Fig~~ure~~ 10 illustrates a snapshot of a test joint.

Formatted: Indent: First line: 0.5"

Fig~~ure~~ 10: IDJ Testing Setup

The simulation was conducted using the finalized parameters and SimScape Multibody model. The results were analyzed to determine if the simulated joints successfully followed the reference trajectories. Since a robotic manipulator consists of multiple joints and links, the focus was on analyzing the coupled dynamics of the entire structure rather than individual joint performance. Assessing the performance of each joint when interconnected through the robot's structure was deemed more meaningful. Moreover, For industrial robotic manipulators, primary

Formatted: Indent: First line: 0.5"

sources of disturbance include environmental interaction, unmodeled dynamics, and gravitational effects (31). All these sources of disturbance are encountered and treated at the system level. Fig~~ure~~ 11 depicts the system-level Simulink model used to simulate the complete system.

Fig~~ure~~ 11: System level Simulink Model for simulating the robotic manipulator and its joints.

The system model shown in Fig~~ure~~ ~~10~~ 11 outlines the simulation flow. A reference trajectory was generated in joint space and applied to the Multibody model of the robot. The model followed the trajectory, while position, velocity, and torque sensors captured corresponding signals. The sensed positions and torques were then used to control the BLDC motors through a PI-based control system. Joints that were capable of accurately following the desired position and torque profile exhibited minimal deviation between the output and reference signals. To quantitatively assess the similarity between the reference and simulated data, Pearson's Correlation Coefficient was calculated using the provided equation. Additionally, visual comparisons were also conducted.

Formatted: Indent: First line: 0.5"

$$r = \frac{\sum(x_i - \bar{x})(y_i - \bar{y})}{\sqrt{\sum(x_i - \bar{x})^2 \sum(y_i - \bar{y})^2}}$$

Where r = Pearson’s Correlation Coefficient, x = value of the first variable, $\bar{x}$ = mean value of the first variable, y = value of the second variable, and $\bar{y}$ = mean value of the second variable.

The Pearson's Correlation Coefficient is a critical metric used to assess the degree of similarity between the reference and simulated torque profiles. It serves as an indicator of the suitability of the direct drive joint at its installed position. A low correlation coefficient value suggests an unsuitable joint, while a value closer to one indicates that the joint is well-suited for its installed position within the robot. The correlation coefficient is specifically calculated for the torque profile, as the correlation coefficients of the position and velocity profiles are dependent on the coefficient of the torque profile. Fig~~ure~~ 12 demonstrates the response of an underpowered joint, highlighting the significance of adequate power for joint performance.

Formatted: Indent: First line: 0.5"

Fig~~ure~~ 12: (a) Position, (b) Velocity, (c) Torque Reference vs Simulation Data of Joint 2 when an underpowered joint was installed at this location.

The underpowered joint resulted in significant deviations in position and velocity responses from the reference trajectory due to its inability to provide the required torque. The generated torque fell short, reaching around 50 Nm compared to the required 70 Nm, leading to a failure in following the trajectory. The correlation coefficient of 0.2593 quantitatively indicated a mismatch between the required and generated torque. To address this issue, we iterated the simulation using different integrated drive joints, gradually moving from low to high power joints. The selection process began with the last joint and moved backward, considering the impact of

Formatted: Indent: First line: 0.5"

later joints on the earlier ones. This selection methodology aimed to identify optimal joints for each position. Intelligent selection methods will be implemented as the library of simulated integrated drive joints expands, currently including frameless BLDC motors and strain wave gears from Maxon, TMotor, and Laifual Drive. Furthermore, we successfully resolved the underpowered joint issue by selecting suitable direct drive joints and tested the robot on a pick-and-place trajectory. Trajectories for position, velocity, and torque were obtained for all five joints of the 5-DOF robot. Through visual inspection, point-by-point comparison, and Pearson's correlation coefficient analysis, we confirmed that the joint trajectories of the simulated model closely followed the reference trajectories, surpassing the defined acceptable level of correlation coefficients greater than 0.95.

For the pick and place application, the reference versus simulated data for all the joints is shown in the ~~figures from figure~~Figs 13 to 17.

Fig~~ure~~ 13: Reference vs Simulation Data of Joint 1's (a) Position, (b) Velocity, and (c) Torque.

Fig~~ure~~ 14: Reference vs Simulation Data of Joint 2's (a) Position, (b) Velocity, and (c) Torque.

Fig~~ure~~ 15: Reference vs Simulation Data of Joint 3's (a) Position, (b) Velocity, and (c) Torque.

Fig~~ure~~ 16: Reference vs Simulation Data of Joint 4's (a) Position, (b) Velocity, and (c) Torque.

Fig~~ure~~ 17: Reference vs Simulation Data of Joint 5's (a) Position, (b) Velocity, and (c) Torque.

The simulation results demonstrate that the simulated integrated drive joint successfully produces the reference trajectory in all cases. The torque difference graph reveals a disparity between the required and simulated torque, but this difference does not result in noticeable deviations in the position or velocity trajectories due to the robustness of the PI controller. Additionally, Table 3 provides the Pearson's Correlation Coefficients for all joints, quantifying the degree of match between the reference and simulated trajectories.

Formatted: Indent: First line: 0.5"

Table 3: Pearson's Correlation Coefficient between Reference and Simulated Torque Profiles of all joints for Pick and Place Trajectory.

Parameter	Joint 1	Joint 2	Joint 3	Joint 4	Joint 5
Correlation Coefficient	0.9969	0.9984	0.9982	0.9992	0.9963

The high correlation coefficients obtained in the simulation experiments highlight the effectiveness of the simulation tool and the chosen motor and harmonic drive combinations in

achieving accurate and reliable robot motion control. These results demonstrate that the simulated robot performance closely aligns with the intended reference trajectories, which is crucial for ensuring precise and consistent robot motion.

Several sources of noise and disturbances including EMI, sensor (encoder) noise, input and output disturbances can cause the manipulator to deviate from an ideal trajectory in the real-life. We analyzed how adequately our~~t~~ PI controller would reject sources of noise disturbances in real-life by lumping these at the output of the speed controller, in the form of a single noise source~~. T.~~ Specifically, this is an ~~-~~additive white g~~G~~aussian noise with unit variance, resulting in a signal-to-noise ratio (SNR) of 15 dB, which is in close agreement with (Fig.2) noise levels observed in real experiments. The Fig 18 depicts the responses of position, velocity, and torque tracking for one of the joints, the joint 3, subject to ~~after the introduction of the~~ noise/disturbance.

Fig 18: Reference vs Simulation data of Joint 3 after introducing disturbance, (a) Position, (b) Velocity, and (c) Torque.

In Fig 18, although there is distortion in the speed and, consequently, in the torque, the PI controller implemented in the position loop adeptly mitigates the effects of the introduced disturbance. This reinforces~~ observation underscores~~ the robustness of the controller. ~~in maintaining the desired trajectory under noisy conditions.~~

This finding carries significant implications for practical applications, as it suggests that physically manufactured robots using the same integrated drive joints and control system can potentially achieve similar performance. The subsequent section presents the results of fabricating the robot based on the integrated drive joints recommended by the simulation studies.

Formatted: Indent: First line: 0.5"

Section IV

Formatted: Font: 18 pt

Hardware Experimentation

As per the results obtained from the simulation experimentation, integrated drive joints were manufactured using the suggested commercially available components. Moreover, to meet the latest industrial standards, BLDC motor controllers from Nanotec® having EtherCAT communication capability were used to drive the BLDC motors. Five such joints were manufactured along with the manipulator's structure. Assembling all the subsystems produced a 5 DOF manipulator, shown in ~~figure~~ Fig 19~~8~~, being controlled via EtherCAT enabled BLDC motor controllers.

Formatted: Indent: First line: 0.5"

Fig~~ure~~ 19~~8~~: Manufactured 5DOF Robot with Integrated Drive Joints performing a pick and place task.

We used Plug & Drive software, provided by Nanotec®, to tune the PI control loops of the motor controllers. The motor controller, just like our simulated motor controller, implements the

Formatted: Indent: First line: 0.5"

PI control loops on three levels, that is the inner most on torque, then on velocity, and the outer most on position. The position and velocity following response of one of the motor controllers before and after tuning is shown in ~~figure~~ Figs 20~~19~~ and 21~~0~~.

Figs~~ure~~ 20~~19~~ and ~~Figure~~ 21~~0~~: Before and After tuning response of position (left) and Velocity (right) of one of the joints. The x-axis shows time in milliseconds, whereas y-axis shows the position and velocity in motor controller units.

The physically developed robot was executed on the same trajectory used in the simulation, and the corresponding torque, velocity, and position data from the integrated drive joints were collected. The collected data was then compared with the simulated data for the pick and place trajectory, as shown in ~~figures~~ Figs 22~~1~~ to 26~~5~~. It is important to note that the time constant of a geared joint prevents any noticeable movement of the output shaft due to current spikes. However, for experimentation purposes, the torque data was obtained from the current sensor in the motor controller and passed through a moving average filter with a window size of 15 samples, approximately equal to 0.15 seconds in time. This moving average filter emulates the damping effect of the geared joint.

Fig~~ure~~ 22~~1~~: Simulated versus Experimental data of Joint 1's (a) Position, (b) Velocity, and (c) Torque.

Fig~~ure~~ 23~~2~~: Simulated versus Experimental data of Joint 2's (a) Position, (b) Velocity, and (c) Torque.

Fig~~ure~~ 24~~3~~: Simulated versus Experimental data of Joint 3's (a) Position, (b) Velocity, and (c) Torque.

Fig~~ure~~ 25~~4~~: Simulated versus Experimental data of Joint 4's (a) Position, (b) Velocity, and (c) Torque.

Fig~~ure~~ 26~~5~~: Simulated versus Experimental data of Joint 5's (a) Position, (b) Velocity, and (c) Torque.

Once again, visual and Pearson's correlation coefficient were used to establish the similarity of the trajectories coming from the simulated model and the actual robot. Table 4 lists the Pearson's Correlation Coefficient for torque profiles of all joints.

Table 4: Pearson's Correlation Coefficient between Simulated and Actual Torque Profiles of all joints for Pick and Place Trajectory.

Parameter	Joint 1	Joint 2	Joint 3	Joint 4	Joint 5
Correlation Coefficient	0.9966	0.9872	0.9882	0.9888	0.9895

Formatted: Indent: First line: 0.5"

Formatted: Indent: First line: 0.5"

Although the correlation coefficients have slightly decreased, they remain within an acceptable range as the position and velocity trajectories are still being accurately followed. However, the mismatches in the torque trajectories require further investigation. The torque impulses observed in the physically manufactured robot are natural and result from current readings taken from the motor controller. These impulses are within the current limits of the BLDC motors and do not cause any damage or deviation in the required position trajectory. Additionally, the average torque deviation from the simulated model remains below 5 Nm for most of the time, which is a small percentage of the nominal torque capability of the integrated drive joints. This additional torque compensates for unmodeled dynamics, friction, and manufacturing variations. Simulation tools are useful for predicting robot behavior but cannot fully account for real-world complexities such as friction and manufacturing variations. However, the position and velocity following performance of the robot is still under acceptable level due to the disturbance rejection and noise cancellation properties of the PI controllers used. These results emphasize the importance of considering real-world complexities and the need for ongoing calibration and optimization. Further investigation will be conducted to minimize the impact of these mismatches in future work.

Furthermore, the repeatability of the developed 5-DOF robot prototype was calculated as per the procedure described in ISO 9283:1998 (32), defined specifically for industrial robots. Dial gauge having positional resolution of 0.01mm was used to measure the position of the end-effector of the robot. Five different positions were commanded to the robot and 100 iterations were carried out for each position to generate the position data. To calculate the positioning repeatability the following calculations were carried out.

$$l_i = \sqrt{(x_i - \bar{x})^2}$$

$$\bar{l} = \frac{1}{n}\sum_{i=1}^{n} l_i$$

Where 'x_i' is the measured value in i^{th} iteration, '$\bar{x}$' is the mean position of all iterations, 'n' is the number of iterations, and '$\bar{l}$' is then the mean positional repeatability according to ANSI/RIA R15.05 standard (33). Moreover, the standard deviation 'S' is then calculated as:

$$S = \sqrt{\sum_{i=1}^{n} \frac{(l_i - \bar{l})^2}{n-1}}$$

Lastly, the positional repeatability, 'Rp', is defined and calculated as

$$Rp = \bar{l} + 3S$$

Table 5 shows the Standard Deviation and the Positional Repeatability for each position. The worst value of the repeatability achieved in this experimentation is claimed as the

Formatted: Indent: First line: 0.5"

Formatted: Indent: First line: 0.5"

repeatability of the developed robot prototype. Comparison with commercially available lightweight robots establishes that this value of repeatability is acceptable for robotic manipulators. The worst case value of repeatability, 0.0503 mm, of our developed robotic manipulator is equivalent to commercially available lightweight robot Techman Robot TM5 (34).

Table 5: Standard Deviation and Repeatability of 5-DOF robot measured and calculated as suggested in ISO 9283:1998 for five different positions. All values are in mm.

Robot	Position 1		Position 2		Position 3		Position 4		Position 5	
	S	Rp	S	Rp	S	Rp	S	Rp	S	Rp
5-DOF	0.0018	0.0080	0.0039	0.0163	0.0062	0.0260	0.0107	0.0503	0.0030	0.0138

Overall, the developed simulation tool demonstrates its potential for robot design and optimization, particularly in the selection of integrated drive joint subcomponents. Using the proposed scheme, the authors along with their research team have also developed a 6-DOF robot manipulator, shown in Figure 276. However, it's still in the testing phase. Figure 276 shows the configuration and hardware of this newly developed manipulator.

Figure 276: Newly developed 6 DOF Robot's FBD (Left) and its hardware (Right).

Section V

Conclusions

In conclusion, this research emphasizes the advantages of adopting a component-driven approach in the design and fabrication of lightweight robotic manipulators. By developing models of commercially available components and integrating them into the design process, the gap between simulation and physical systems can be significantly reduced, leading to elimination or reduction of various steps involved in the robotic manipulator development process.

The presented framework demonstrates the effectiveness of this approach through the modeling and analysis of integrated drive joints in a 5-DOF lightweight robotic manipulator. Notably, the experimental evaluation of the developed robotic system yielded impressive results. The positional repeatability, assessed in accordance with ISO 9283:1998, demonstrated a repeatability of 0.05 mm, a performance level comparable to the Techman Robot TM5, a commercially available lightweight robot.

Subsequent hardware experimentation, where the same integrated drive joints and robot structure were manufactured and driven on predetermined trajectories, revealed that while position and velocity trajectories closely matched the simulated model, a discernible difference in torque requirements was observed. This disparity can be attributed to unmodeled dynamics,

Formatted: Indent: First line: 0.5"

Formatted: Font: 18 pt

Formatted: Indent: First line: 0.5"

such as friction arising from strain wave gears and misalignments in the assembly process. Nevertheless, the magnitude of the torque difference remained small compared to the nominal torque capability of the joints.

The proposed simulation tool and its framework hold significant potential in revolutionizing the robot manufacturing industry. By enabling developers working in educational research institutes, robotics startups, and other similar positions, to simulate robotic manipulators using models of actual subcomponents, it offers substantial time, effort, and investment savings in today's fast-paced and resource-intensive environment.

Future research endeavors will focus on further refining the simulation tool, expanding the library of simulated models to incorporate components from various manufacturers, and addressing the challenges posed by unmodeled dynamics. This ongoing development will continue to enhance the accuracy, reliability, and practicality of the proposed framework, further solidifying its potential as a game-changer in the field of robot manufacturing.

In conclusion, the component-driven approach presented in this research has demonstrated its effectiveness in facilitating the rapid development of lightweight robotic manipulators. By leveraging commercially available components and optimizing their integration through simulation and hardware experimentation, this framework offers valuable insights and practical solutions for the advancement of robotic systems.

Acknowledgements

This research is supported by ~~was made possible by the funding provided by~~ the Higher Education Commission (HEC) Pakistan ~~for~~under the project ‘T~~t~~he establishment of~~ of Human-Centered Robotics Lab (HCRL) under the ambit of~~ National Center for Robotics and Automation (NCRA) with grant number NRPU-16242. We would also like to thank Dr. Ali Raza, Principal Investigator (PI) of Human Centered Robotics Lab UET Lahore~~HCRL,~~ for his unwavering support and guidance.

References

1. Akundi A, Euresti D, Luna S, Ankobiah W, Lopes A, Edinbarough I. State of Industry 5.0—Analysis and identification of current research trends. Applied System Innovation. 2022;5(1):27.
2. Hirzinger G, Albu-Schaffer A, Hahnle M, Schaefer I, Sporer N, editors. On a new generation of torque controlled light-weight robots. Proceedings 2001 ICRA IEEE International Conference on Robotics and Automation (Cat No 01CH37164); 2001: IEEE.
3. Popić S, Miloradović B. Light weight robot arms-an overview. INFOTEH-JAHORINA. 2015;14.
4. Balding S, Gning A, Cheng Y, Iqbal J. Information Rich Voxel Grid for Use in Heterogeneous Multi-Agent Robotics. Applied Sciences. 2023;13(8):5065.
5. Bilal M, Rizwan M, Maqbool HF, Ahsan M, Raza A. Design optimization of powered ankle prosthesis to reduce peak power requirement. Science Progress. 2022;105(3):00368504221117895.

Formatted: Font: 18 pt

Formatted: Justified

6. Naeem A, Rizwan M, Maqbool HF, Ahsan M, Raza A, Abouhossein A, et al. Virtual constraint control of Knee-Ankle prosthesis using an improved estimate of the thigh phase-variable. Biomedical Signal Processing and Control. 2022;73:103366.
7. Calvo R, Gil P. Evaluation of collaborative robot sustainable integration in manufacturing assembly by using process time savings. Materials. 2022;15(2):611.
8. Gheorghe G, editor Concepts and mechatronics and cyber-mixmechatronics constructions, integrated in COBOT type technology platform for intelligent industry (4.0). Proceedings of the International Conference of Mechatronics and Cyber-MixMechatronics–2019 3; 2020: Springer.
9. Timms MJ. Letting artificial intelligence in education out of the box: educational cobots and smart classrooms. International Journal of Artificial Intelligence in Education. 2016;26:701-12.
10. Djuric AM, Urbanic R, Rickli J. A framework for collaborative robot (CoBot) integration in advanced manufacturing systems. SAE International Journal of Materials and Manufacturing. 2016;9(2):457-64.
11. Chung JC, Hwang T-S, Wu C-T, Jiang Y, Wang J-Y, Bai Y, et al. Framework for integrated mechanical design automation. Computer-aided design. 2000;32(5-6):355-65.
12. SANCHEZ S. Career Karma2022. Available from: https://careerkarma.com/blog/how-to-build-your-own-robot/#:~:text=How%20Long%20Does%20It%20Take,mechanical%20and%20electrical%20assembly%20expertise.
13. Ruishu Z, Chang Z, Weigang Z, editors. The status and development of industrial robots. IOP Conference Series: Materials Science and Engineering; 2018: IOP Publishing.
14. BRACJUNOVÁ VL. Robots take the robot out of the human: Soitron Group; 2019 [Available from: https://www.soitron.com/robots-take-the-robot-out-of-the-human/#:~:text=Creating%20a%20new%20robot%20from,part%20of%20the%20development%20process.
15. Hirose S, Arikawa K. Coupled and decoupled actuation of robotic mechanisms. Advanced Robotics. 2001;15(2):125-38.
16. Tarkian M, O¨ lvander J, Feng X, Petterson M, editors. Design automation of modular industrial robots. International Design Engineering Technical Conferences and Computers and Information in Engineering Conference; 2009.
17. Covell DM. Top-Down & Bottom-Up Approaches to Robot Design: West Virginia University; 2022.
18. Zimmermann M, Königs S, Niemeyer C, Fender J, Zeherbauer C, Vitale R, et al. On the design of large systems subject to uncertainty. Journal of Engineering Design. 2017;28(4):233-54.
19. Sathuluri A, Sureshbabu AV, Frank J, Amm M, Zimmermann M. Computational Systems Design of Low-Cost Lightweight Robots. Robotics. 2023;12(4):91.
20. Walden DD. Systems engineering handbook: A guide for system life cycle processes and activities. (No Title). 2015.
21. Albu ‐ Schäffer A, Haddadin S, Ott C, Stemmer A, Wimböck T, Hirzinger G. The DLR lightweight robot: design and control concepts for robots in human environments. Industrial Robot: an international journal. 2007.
22. Bolignari M, Fontana M. Design and experimental characterization of a high performance hydrostatic transmission for robot actuation. Meccanica. 2020;55(5):1169-79.
23. Mo S, Li X, Yang Z-n, Zhou C-p, Gao H-j, Cen G-j, et al. Integrated design of brushless motor drive and control system for robot joints. Journal of Central South University. 2021;28(12):3818-28.
24. Bischoff R, Kurth J, Schreiber G, Koeppe R, Albu-Schäffer A, Beyer A, et al., editors. The KUKA-DLR Lightweight Robot arm-a new reference platform for robotics research and manufacturing. ISR 2010 (41st international symposium on robotics) and ROBOTIK 2010 (6th German conference on robotics); 2010: VDE.

25. Mohanraj D, Aruldavid R, Verma R, Sathyasekar K, Barnawi AB, Chokkalingam B, et al. A Review of BLDC Motor: State of Art, Advanced Control Techniques, and Applications. IEEE Access. 2022.
26. Routh B, editor Design aspects of harmonic drive gear and performance improvement of its by problems identification: A review. AIP conference proceedings; 2018: AIP Publishing LLC.
27. Machado TDN, Sousa M, Monteiro S, Bicho E. CoopDynSim: a 3D robotics simulator. 2012.
28. Baizid K, Ćuković S, Iqbal J, Yousnadj A, Chellali R, Meddahi A, et al. IRoSim: Industrial Robotics Simulation Design Planning and Optimization platform based on CAD and knowledgeware technologies. Robotics and Computer-Integrated Manufacturing. 2016;42:121-34.
29. Bose BK. Modern power electronics and AC drives: Prentice hall Upper Saddle River, NJ; 2002.
30. Gutiérrez S, Zotovic R, Navarro M, Meseguer M. Design and manufacturing of a prototype of a lightweight robot arm. Procedia Manufacturing. 2017;13:283-90.
31. San-Miguel A, Puig V, Alenyà G. Disturbance observer-based LPV feedback control of a N-DoF robotic manipulator including compliance through gain shifting. Control Engineering Practice. 2021;115:104887.
32. Standardization TIOf. ISO 9283:1998(en) Manipulating industrial robots — Performance criteria and related test methods. 1998.
33. Institute ANS. American National Standard for Industrial Robots and Robot Systems - Point-to-Point and Static Performance Characteristics – Evaluation. ANSI/RIA R1505-1-1990 (R1999)1999.
34. TECHMANROBOTINC. TM5 - 900 2019 [Available from: https://www.tm-robot.com/en/tm5-900/.

Dear Editor,

We appreciate insightful comments and suggestions on our submitted manuscript. We take this opportunity to address the comments/concerns raised by you or by the reviewers in the revised version of our manuscript. Point-by-point responses are given below.

Comments of the Academic Editor and Responses

Comment # 1. When submitting your revision, we need you to address these additional requirements.

Please ensure that your manuscript meets PLOS ONE's style requirements, including those for file naming. The PLOS ONE style templates can be found at https://journals.plos.org/plosone/s/file?id=wjVg/PLOSOne_formatting_sample_main_body.pdf and https://journals.plos.org/plosone/s/file?id=ba62/PLOSOne_formatting_sample_title_authors_affiliations.pdf.

Response: We have updated our manuscript as per the formatting guidelines provided and hope that now the manuscript meets the formatting requirements of the PLOS ONE journal.

Comment # 2. Please note that PLOS ONE has specific guidelines on code sharing for submissions in which author-generated code underpins the findings in the manuscript. In these cases, all author-generated code must be made available without restrictions upon publication of the work. Please review our guidelines at https://journals.plos.org/plosone/s/materials-and-software-sharing#loc-sharing-code and ensure that your code is shared in a way that follows best practice and facilitates reproducibility and reuse.

Response: We fully understand the guidelines on code sharing and the code files shall be made available when required.

Comment # 3. We note that Figure(s) 2, 5, 6, 7, 8, 9, 10, 18 and 26 in your submission contain copyrighted images. All PLOS content is published under the Creative Commons Attribution License (CC BY 4.0), which means that the manuscript, images, and Supporting Information files will be freely available online, and any third party is permitted to access, download, copy, distribute, and use these materials in any way, even commercially, with proper attribution. For more information, see our copyright guidelines: http://journals.plos.org/plosone/s/licenses-and-copyright.

We require you to either (1) present written permission from the copyright holder to publish these figures specifically under the CC BY 4.0 license, or (2) remove the figures from your submission:

a. You may seek permission from the original copyright holder of Figure(s) 2, 5, 6, 7, 8, 9, 10, 18 and 26 to publish the content specifically under the CC BY 4.0 license.

We recommend that you contact the original copyright holder with the Content Permission Form (http://journals.plos.org/plosone/s/file?id=7c09/content-permission-form.pdf) and the following text: "I request permission for the open-access journal PLOS ONE to publish XXX under the Creative Commons Attribution License (CCAL) CC BY 4.0 (http://creativecommons.org/licenses/by/4.0/). Please be aware that this license allows unrestricted use and distribution, even commercially, by third parties. Please reply and provide explicit written permission to publish XXX under a CC BY license and complete the attached form."

Please upload the completed Content Permission Form or other proof of granted permissions as an ""Other"" file with your submission.

In the figure caption of the copyrighted figure, please include the following text: “Reprinted from [ref] under a CC BY license, with permission from [name of publisher], original copyright [original copyright year].”

b. If you are unable to obtain permission from the original copyright holder to publish these figures under the CC BY 4.0 license or if the copyright holder’s requirements are incompatible with the CC BY 4.0 license, please either i) remove the figure or ii) supply a replacement figure that complies with the CC BY 4.0 license. Please check copyright information on all replacement figures and update the figure caption with source information. If applicable, please specify in the figure caption text when a figure is similar but not identical to the original image and is therefore for illustrative purposes only.

Response: We have obtained the permission of the copyright holder, who is the Principal Investigator (PI) of the Human-Centered Robotics Lab, under which the whole research is being carried out. Moreover, Muhammad Ahsan (2nd author) is also the Co-PI of the same lab. The completed Content Permission Form is attached in the relevant section. Furthermore, it is stated that figures created using MATLAB software does not contain any MATLAB code files supplied from MATLAB’s system.

Comment # 4. Please amend the manuscript submission data (via Edit Submission) to include author Muhammad Ahsan.

Response: We have updated the author data.

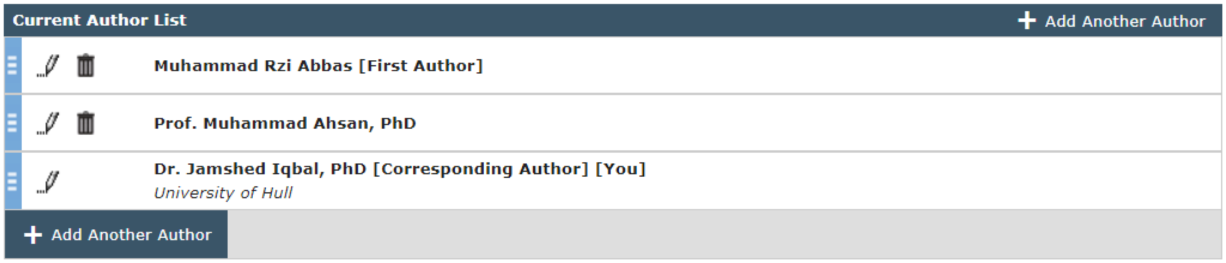

Comment # 5. Please amend your authorship list in your manuscript file to include author Ahsan Ali.

Response: There was a typing error in the name. The name ‘Ahsan Ali’ is in fact ‘Muhammad Ahsan’. We have addressed this error.

Comments of Reviewer # 1 and Responses

Comment # 1. The authors should delve into existing literature and cite papers that address the reduction of the conventional design cycle in robotics.

Response: We have reinforced the literature review at the start of Section II by including multiple latest references related to the optimization of design cycle in robotics. The newly added discussion may be found at the start of Section II.

Comment # 2. Although a PI controller was utilized for the motor, the authors did not explore alternative controllers like PID or PD. It remains unclear why other controller types were not considered. Further investigation into the choice of the PI controller and the omission of PID or PD controllers could provide valuable insights into the motor control strategy adopted in this study. The authors can provide justification through simulation results as well.

Response: In the second paragraph of the subsection titled "Frameless BLDC Motor Model," we explicitly mention that commercial motor drives may utilize PI, PD, or PID controllers for motor control. However, in the subsequent paragraph (para 3 of the same subsection), we clarify that the specific hardware motor controllers employed in our study exclusively incorporate a PI control loop. Consequently, our decision to simulate only the PI controller is reflective of the actual control mechanism embedded in the hardware utilized for this research.

Moreover, it is noteworthy that commercial BLDC motor controllers commonly employ PI controllers due to their effectiveness in various applications. This aligns with the prevalent industry practice, and it was this real-world consideration that guided our simulation approach.

To further elucidate this point, we have refined the discussion in the relevant section of the manuscript to ensure clarity on our rationale for simulating the PI controller exclusively.

Comment # 3. The authors presented simulation and experimental results, yet they should further validate the design approach's feasibility by exploring additional scenarios, particularly under diverse disturbance conditions. Demonstrating the system's performance in the face of various disturbances would enhance the comprehensiveness and robustness of their findings.

Response: For industrial robotic manipulators, primary sources of disturbance include environmental interaction, unmodeled dynamics, and gravitational effects. However, it's important to note that the scope of our research primarily focuses on assessing the impact of commercially available components on the overall robotic manipulator design.

Interaction with the environment involves torque control schemes implemented at the system level, and its consideration is often application dependent. As such, it falls beyond the specific objectives of our study. Regarding unmodeled dynamics, our experimentation explicitly addresses the system-level robustness against such dynamics, as mentioned in the manuscript. Lastly, the SimScape dynamic simulation accounts for gravitational effects in our model. The demonstrated positional accuracy in both simulation and hardware experimentation indicates that gravitational effects are appropriately managed throughout the trajectory, leveraging additional torque well within the actuator's specified limits.

To further exhibit the strength of our simulated models and in alignment with your suggestion, we have conducted additional simulations introducing white Gaussian noise at the individual motor level to

specifically evaluate the efficacy of the individual PI controller in rejecting such disturbances. We have incorporated corresponding discussions and graphical data, Fig 18, in the relevant section of the manuscript, para 3, 9 and 10 of Section III Simulation Experimentation. These additions aim to provide a more comprehensive understanding of the disturbance rejection capabilities at the individual motor level.

Comment # 4. The authors didn't follow the formatting guidelines of the journal available on the Plos One website.

Response: We have updated the formatting as per the guidelines provided by the academic editor.

Comment # 5. The references are not formatted according to the "International Committee of Medical Journal Editors (ICMJE)". The reference should include all the information, such as the month of publication, and place in case of a conference.

Response: We have changed the reference style to that of ICMJE.

Comment # 6. Please report the values in SI units in Table 1.

Response: We have converted all the values to SI units in Table 1.

We trust that these updates contribute significantly to addressing your concerns and further enriching the content of our manuscript. We look forward to any additional guidance or feedback you may have.

Thank you for your time and consideration.
