# Supplementary material for: Experimental development of lightweight manipulators with improved design cycle time that leverages off-the-shelf robotic arm components
Source: PLoS One. 2024 Jul 18;19(7):e0305379. doi: 10.1371/journal.pone.0305379 (PMC11257280; doi:10.1371/journal.pone.0305379)
Supplement: Attachment — Submitted filename: PONE-D-23-31418_R2_reviewer.pdf [file pone.0305379.s004.pdf]

PLOS ONE

Experimental Development of Lightweight Manipulators with Improved Design Cycle Time that leverages off-the-shelf Robotic Arm Components

--Manuscript Draft--

Manuscript Number:	PONE-D-23-31418R2
Article Type:	Research Article
Full Title:	Experimental Development of Lightweight Manipulators with Improved Design Cycle Time that leverages off-the-shelf Robotic Arm Components
Short Title:	Robotic Manipulator - Design and Experimentation
Corresponding Author:	Jamshed Iqbal, PhD<br>University of Hull<br>Hull, County (optional) UNITED KINGDOM
Keywords:	Lightweight robot; cobot; robot design process
Abstract:	The growing market for lightweight robots inspires new use-cases, such as collaborative manipulators for human-centered automation. However, widespread adoption faces obstacles due to high R&D costs and longer design cycles, although rapid advances in mechatronic engineering have effectively narrowed the design space to affordable robot components, turning the development of lightweight robots into a component selection and integration challenge. Recognizing this transformation, we demonstrate a practical framework for designing lightweight industrial manipulators using a case-study of indigenously developed 5 Degrees-of-Freedom (DOF) cobot prototype. Our framework incorporates off-the-shelf sensors, actuators, gears, and links for Design for Manufacturing and Assembly (DFMA), along with complete virtual prototyping. The design cycle time is reduced by approximately 40% at the cost of cobot real-time performance deviating within 2.5% of the target metric. Our physical prototype, having repeatability of 0.05mm calculated as per the procedure defined in ISO 9283:1998, validates the cost-effective nature of the framework for creating lightweight manipulators, benefiting robotic startups, R&D organizations, and educational institutes without access to expensive in-house fabrication setups.
Order of Authors:	Muhammad Rzi Abbas
	Muhammad Ahsan, PhD
	Jamshed Iqbal, PhD
Response to Reviewers:	Response to reviewers file is attached for your kind review.
Additional Information:	
**Question**	**Response**
**Financial Disclosure**<br><br>Enter a financial disclosure statement that describes the sources of funding for the work included in this submission. Review the submission guidelines for detailed requirements. View published research articles from *PLOS ONE* for specific examples.<br><br>This statement is required for submission and **will appear in the published article** if the submission is accepted. Please make sure it is accurate.	Yes

**Funded studies**<br>Enter a statement with the following details:<br>• Initials of the authors who received each award<br>• Grant numbers awarded to each author<br>• The full name of each funder<br>• URL of each funder website<br>• Did the sponsors or funders play any role in the study design, data collection and analysis, decision to publish, or preparation of the manuscript?<br><br>Did you receive funding for this work?	
Please add funding details.<br>as follow-up to "**Financial Disclosure**<br><br>Enter a financial disclosure statement that describes the sources of funding for the work included in this submission. Review the submission guidelines for detailed requirements. View published research articles from *PLOS ONE* for specific examples.<br><br>This statement is required for submission and **will appear in the published article** if the submission is accepted. Please make sure it is accurate.<br><br>**Funded studies**<br>Enter a statement with the following details:<br>• Initials of the authors who received each award<br>• Grant numbers awarded to each author<br>• The full name of each funder<br>• URL of each funder website<br>• Did the sponsors or funders play any role in the study design, data collection and analysis, decision to publish, or preparation of the manuscript?<br><br>Did you receive funding for this work?"	Grant Number: NRPU-16242 awarded by Higher Education Commission Pakistan. The funders had no role in study design, data collection and analysis, decision to publish, or preparation of the manuscript.
Please select the country of your main research funder (please select carefully as in some cases this is used in fee	

calculation).<br>as follow-up to "**Financial Disclosure**<br><br>Enter a financial disclosure statement that describes the sources of funding for the work included in this submission. Review the submission guidelines for detailed requirements. View published research articles from *PLOS ONE* for specific examples.<br><br>This statement is required for submission and **will appear in the published article** if the submission is accepted. Please make sure it is accurate.<br><br>**Funded studies**<br>Enter a statement with the following details:<br>• Initials of the authors who received each award<br>• Grant numbers awarded to each author<br>• The full name of each funder<br>• URL of each funder website<br>• Did the sponsors or funders play any role in the study design, data collection and analysis, decision to publish, or preparation of the manuscript?<br><br>Did you receive funding for this work?"	
**Competing Interests**<br><br>Use the instructions below to enter a competing interest statement for this submission. On behalf of all authors, disclose any competing interests that could be perceived to bias this work—acknowledging all financial support and any other relevant financial or non-financial competing interests.<br><br>This statement is required for submission and **will appear in the published article** if the submission is accepted. Please make sure it is accurate and that any funding sources listed in your Funding Information later in the submission form are also declared in your Financial Disclosure	The authors have declared that no competing interests exist.

statement.<br><br>View published research articles from *PLOS ONE* for specific examples.<br><br>**NO authors have competing interests**<br><br>Enter: *The authors have declared that no competing interests exist.*<br><br>**Authors with competing interests**<br><br>Enter competing interest details beginning with this statement:<br><br>*I have read the journal's policy and the authors of this manuscript have the following competing interests: [insert competing interests here]*<br><br>* typeset	
**Ethics Statement**<br><br>Enter an ethics statement for this submission. This statement is required if the study involved:<br><br>• Human participants<br>• Human specimens or tissue<br>• Vertebrate animals or cephalopods<br>• Vertebrate embryos or tissues<br>• Field research<br><br>Write "N/A" if the submission does not require an ethics statement.<br><br>General guidance is provided below. Consult the submission guidelines for detailed instructions. **Make sure that all information entered here is included in the Methods section of the manuscript.**	N/A

**Format for specific study types**<br><br>**Human Subject Research (involving human participants and/or tissue)**<br>• Give the name of the institutional review board or ethics committee that approved the study<br>• Include the approval number and/or a statement indicating approval of this research<br>• Indicate the form of consent obtained (written/oral) or the reason that consent was not obtained (e.g. the data were analyzed anonymously)<br><br>**Animal Research (involving vertebrate animals, embryos or tissues)**<br>• Provide the name of the Institutional Animal Care and Use Committee (IACUC) or other relevant ethics board that reviewed the study protocol, and indicate whether they approved this research or granted a formal waiver of ethical approval<br>• Include an approval number if one was obtained<br>• If the study involved *non-human primates*, add *additional details* about animal welfare and steps taken to ameliorate suffering<br>• If anesthesia, euthanasia, or any kind of animal sacrifice is part of the study, include briefly which substances and/or methods were applied<br><br>**Field Research**<br><br>Include the following details if this study involves the collection of plant, animal, or other materials from a natural setting:<br>• Field permit number<br>• Name of the institution or relevant body that granted permission	
**Data Availability**<br><br>Authors are required to make all data underlying the findings described fully available, without restriction, and from the time of publication. PLOS allows rare exceptions to address legal and ethical concerns. See the PLOS Data Policy and FAQ for detailed information.	Yes - all data are fully available without restriction

A Data Availability Statement describing where the data can be found is required at submission. Your answers to this question constitute the Data Availability Statement and **will be published in the article**, if accepted.<br><br>**Important:** Stating 'data available on request from the author' is not sufficient. If your data are only available upon request, select 'No' for the first question and explain your exceptional situation in the text box.<br><br>Do the authors confirm that all data underlying the findings described in their manuscript are fully available without restriction?	
**Describe where the data may be found in full sentences. If you are copying our sample text, replace any instances of XXX with the appropriate details.**<br><br>• If the data are **held or will be held in a public repository**, include URLs, accession numbers or DOIs. If this information will only be available after acceptance, indicate this by ticking the box below. For example: *All XXX files are available from the XXX database (accession number(s) XXX, XXX.)*.<br>• If the data are all contained **within the manuscript and/or Supporting Information files**, enter the following: *All relevant data are within the manuscript and its Supporting Information files.*<br>• If neither of these applies but you are able to provide **details of access elsewhere**, with or without limitations, please do so. For example:<br><br>*Data cannot be shared publicly because of [XXX]. Data are available from the XXX Institutional Data Access / Ethics Committee (contact via XXX) for researchers who meet the criteria for access to confidential data.*<br><br>*The data underlying the results presented in the study are available from (include the name of the third party*	All relevant data are within the manuscript.

and contact information or URL).<br>• This text is appropriate if the data are owned by a third party and authors do not have permission to share the data.<br><br>* typeset	
Additional data availability information:	

Powered by Editorial Manager® and ProduXion Manager® from Aries Systems Corporation

Manuscript

Click here to access/download;Manuscript;Clean_Manuscript_Submitted.docx

Experimental Development of Lightweight Manipulators with Improved Design Cycle Time that leverages off-the-shelf Robotic Arm Components

Muhammad Rzi Abbas[1], Muhammad Ahsan[1], Jamshed Iqbal[2*]

[1]Department of Mechatronics and Control Engineering, University of Engineering and Technology, Lahore, Pakistan

[2]School of Computer Science, Faculty of Science and Engineering, University of Hull, United Kingdom

*Corresponding author: j.iqbal@hull.ac.uk

Abstract

The growing market for lightweight robots inspires new use-cases, such as collaborative manipulators for human-centered automation. However, widespread adoption faces obstacles due to high R&D costs and longer design cycles, although rapid advances in mechatronic engineering have effectively narrowed the design space to affordable robot components, turning the development of lightweight robots into a component selection and integration challenge. Recognizing this transformation, we demonstrate a practical framework for designing lightweight industrial manipulators using a case-study of indigenously developed 5 Degrees-of-Freedom (DOF) cobot prototype. Our framework incorporates off-the-shelf sensors, actuators, gears, and links for Design for Manufacturing and Assembly (DFMA), along with complete virtual prototyping. The design cycle time is reduced by approximately 40% at the cost of cobot real-time performance deviating within 2.5% of the target metric. Our physical prototype, having repeatability of 0.05mm calculated as per the procedure defined in ISO 9283:1998, validates the cost-effective nature of the framework for creating lightweight manipulators, benefiting robotic startups, R&D organizations, and educational institutes without access to expensive in-house fabrication setups.

Keywords

Lightweight robot, Cobot, Robot Design Process

Section I

Introduction

The field of robotics has undergone a significant evolution, particularly with the emergence of lightweight robots, aligning seamlessly with the requisites of Industry 5.0 (1). This

progression has paved the way for substantial advancements in collaborative robotics, where lightweight robots, often referred to as "cobots," are tailored for harmonious human-robot interaction in applications ranging from industrial collaboration to medical-related scenarios (2-6). These versatile entities boast characteristics that position them as adaptable collaborators within cooperative work environments. Their intrinsic attributes, characterized by reduced speeds and inertias, make them particularly conducive for seamless interaction with human counterparts, leading to a broadening spectrum of applications across diverse domains. From assembly line installations (7) to medical setups (8), from education sector (9) to space endeavors (10) , these robots have found their utility across varied industries. The escalating demand for automation positions lightweight robots as pivotal players across industrial sectors (1). In this evolving landscape, our research takes on heightened significance, strategically addressing the imperative for streamlined design and development of lightweight robots, a cornerstone in facilitating the transition toward Industry 5.0-aligned industrial and R&D ecosystems.

Conventional engineering product design cycle (11) is illustrated in Fig. 1 which is used to argue about robotic product development cycle. The whole process may be divided into seven major stages as labeled by circled digits in the figure.

Figure 1: Typical Robot Design and Development Cycle. The table in the figure shows typically what percentage of total design cycle time (12-14) is spent on each stage. The portions enclosed in Red Boxes are either eliminated or simplified through the proposed scheme.

- Stage 1 is the initial stage where the concept of the robot is formulated, and its basic design is outlined. It involves defining the robot's purpose, capabilities, and basic architecture. Typically, 10%[1] (12-14) of the total design cycle time is spent in this stage.
- Stage 2 relates to the detailed design of the robot's components, such as mechanical, electrical, and software aspects. Planning for the manufacturing and assembly processes also occurs in this stage, and it takes around 20% of the total design cycle time.
- Stage 3 implements the initial design, usually in simulated environments, which is a quicker method, or sometimes a physical prototype is also developed, that normally takes longer. Physical prototype development consumes around 15% of the design cycle time.
- Stage 4 tests and iterates the prototype based on several performance parameters. Typically, 15% of the design cycle time is spent on this activity.
- Stage 5 starts once the design is optimized and tested. The final version of the robot design is prepared for manufacturing. This includes sourcing components, manufacturing, and assembly. Normally 20% of the total design cycle time is utilized in this stage.

[1] These values are based on general industry knowledge and practices. The breakdown of time spent on different stages of robot development can vary widely depending on the specific project, organization, and goals. These percentages are meant to give a rough idea of how time may classically be allocated in a typical robot development process.

- Stage 6 tests and validates the manufactured robot to make it deployment ready. This stage typically consumes 15% of the total design cycle time.
- Stage 7 is related to production of documentation, user manuals, training material and deployment of the robot in the intended environment. This stage lasts around 5% of the total design cycle time.

Considering the extensive range of commercially available components, it would be both economical and timesaving for developers to simulate robotic manipulators exclusively composed of such components. The design approach presented in this paper modifies the robot development process along these lines, hence reducing the complexity and required time. First, stage 1 is reduced to only gathering of the robot specifications. Currently, these specifications are the degrees-of-freedom, payload capability, reach, and maximum workspace speed of the end-effector. The time needed to develop initial conceptual design based on the given specifications will be reduced because of the developed library of components. Once the library of subcomponents has been established, our proposed scheme will only use validated models of commercially available components to design the robotic manipulator. Hence stage 1 can almost be eliminated resulting in saving of around 10% of the total design cycle time. Second, the modeling of the subcomponents allows for virtual prototyping, eliminating the need for physical prototype development, saving additional 10% of the total design cycle time by simplifying stage 2. Moreover, the developed library eliminates the requirement of subcomponents' performance analysis, further saving valuable development time. Lastly, the testing and validation of the manufactured robot is greatly reduced because the virtual prototype is made of all commercially available or produced components. The Pearson's Correlation Coefficient and Root Mean Squared Error (RMSE) of trajectory tracking presented in the experimentation section of this paper, between the performance of the virtual prototype and the developed robot confirms this claim. Lastly, for the functional performance validation, our criterion requires that the physical prototype attains repeatability of the order of magnitude same as that of the commercially available lightweight robot. Table 7 in Section IV Hardware Experimentation validates that our developed prototype exhibits the desired performance.

This research work focuses on the simulation of hollow shaft Integrated Drive Joints (IDJs) integrated into lightweight robotic manipulators designed to operate as cobots. The developed library encompasses models of several commercially available subcomponents. Within the simulation environment, the IDJ models and the manipulator's structure are utilized to identify the suitable components, the frameless BLDC Motor and the strain wave gear, for the integrated drive joints. Moreover, the simulation of the complete structure enables the consideration of coupled dynamics throughout the entire system, which is significant to control system engineers (15). While a single IDJ exhibits distinct performance characteristics, coupling multiple joints introduces coupled dynamic effects across all joints (16, 17). These couplings can be mitigated at the structural level through the appropriate selection of gearing systems or addressed at the software level by fine-tuning the control system accordingly.

The simulation tool was developed using the MATLAB/Simulink R2021b environment, while the robot structure was modeled using SolidWorks 2021. Subsequently, based on the findings obtained from the simulation tool, a 5 degrees-of-freedom (DOF) lightweight robot was developed, as depicted in Fig. 2. Though the authors have also developed a 6-DOF lightweight robot by following the same footsteps, but for the sake of compactness of the article, the detailed analysis of 5-DOF robot is presented.

Figure 2: 5 DOF Robot's FBD (Left) and 5 DOF robot's hardware (Right).

Table 1: Man-hours saved in result of following the proposed scheme for designing and developing a 5 Degrees of freedom Lightweight Robotic Manipulator in a typical R&D Lab

**Stage**	**Title**	**Conventional Design Approach's Initial R&D man-hour estimate**	**Actual R&D man-hours under the proposed scheme**	**Man-hours saved**	**Percentage of the Design Cycle Time saved**
1	Conceptual Design	160	32	128	5.9
2	Detailed Design	320	320	0	0.0
3	Assembly Design Model	480	480	0	0.0
4	Design Analysis	320	64	256	11.9
5	Fabrication	400	400	0	0.0
6	Hardware Iterations	480	0	480	22.2
7	Commissioning	80	80	0	0.0
**Total Percentage of saved Design Cycle Time**					**38.6**

Table 1 summarizes the potential time savings offered by the proposed method for developing robotic manipulators. This data, based on research conducted at the Human Centered Robotics Lab, demonstrates a 38.6% reduction in the total design cycle time. The table itself is divided into six columns. The first two columns list the distinct stages involved in a typical engineering design process for a product. The third column displays estimated man-hours required to complete each stage under the conventional design approach, described in (11).

Equation 1, shown below, details the calculation used to determine these man-hour values.

$$Man\ hours = P\ x\ D\ x\ W \qquad (1)$$

Where:

- P = Number of personnel assigned to a stage
- D = Number of days required to complete a stage

- W = Working hours per day

Column 4 details the actual man-hours consumed during the design and development of a 5-DOF lightweight robotic arm. Column 5 quantifies the man-hours saved by implementing the proposed approach, while Column 6 expresses these savings as a percentage. The sum of these percentages demonstrates the overall 38.6% reduction in design cycle time. It is important to note that the time distribution across different design and development stages may deviate from the general percentage values presented in Fig. 1.

The organization of the remaining manuscript is as follows: Section II describes the challenges involved in the in-house development of light-weight robots and then propose a suitable methodology for generating an IDJ model that includes a synergetic combination of frameless brushless DC (BLDC) motor, strain wave gear and other subcomponents. Section III showcases the simulation results of the developed model, whereas Section IV details the performance results of the physically developed 5 DOF manipulator based on the in-horse manufactured integrated drive joint using the same hardware subcomponents as those used in the simulation. Lastly, Section V concludes the manuscript and steers the reader to possible avenues of future work.

Section II

Addressing challenges involved in the in-house development of Light-Weight Robots

Robot design methodologies encompass both bottom-up and top-down approaches, which have been extensively explored in literature. However, a critical gap remains: many analyses neglect the integration of commercially available, off-the-shelf components into the design process. Bottom-up strategies are well-suited for developing general-purpose robots with broad functionalities, while top-down approaches excel at creating robots specifically tailored for particular applications (18). The top-down paradigm initiates the design process with a clear focus on the final application, resulting in a more streamlined design space. The recent studies (19) highlight the efficacy of top-down approaches in mitigating cyclic dependencies, particularly beneficial for the efficient design of mechatronic systems characterized by intricate interdependencies. Another relevant contribution to this field comes from a study that optimized reconfigurable collaborative robots, focusing on the importance of modularity and structural design (20). This innovative top-down approach draws inspiration from the V-model employed in the automotive and aerospace industries, as introduced in (21). This optimization strategy aims to improve development cost-effectiveness by optimizing the physical structure, which in turn reduces manufacturing material costs. Additionally, leveraging established modular interfaces significantly cuts down on development time compared to customizing interfaces for each module, as detailed in (20). These recent

studies together highlight the changing nature of design processes. They reveal the valuable advantages of integrating off-the-shelf components within a top-down approach.

Like any robotic system, lightweight manipulators adhere to basic design principles and employ specific components to achieve their lightweight characteristics (22). The IDJ or Direct Drive Joint (DDJ) incorporates frameless BLDC Motors and Strain Wave Gears, distinct from conventional manipulators. Additionally, lightweight manipulators utilize differently designed links made from lightweight materials. Over the past two decades, researchers have proposed various drive unit designs suitable for lightweight robots (22-25). The design constraints imposed by the hollow shafts of drive units and of links connecting the drive units to form the kinematic chain, require specific component choices. For example, both the flexspline of the strain wave gear and the lightweight structure of the robot contribute to its overall mechanical flexibility, necessitating the consideration of dynamic coupling among multiple drive units through lightweight links. Consequently, optimizing the subcomponents of drive units and links becomes a primary objective in the designing and manufacturing of lightweight robots. Moreover, considering the extensive experience in the development of robotic manipulator components (26, 27), it is prudent to leverage this existing knowledge to narrow down the design space. Furthermore, researchers have also proposed schemes to optimize the selection of off-the-shelf motors, both AC Servos (28) and BLDC Motors (29), that can enhance the performance of already built manipulators. These studies analyze the dynamic behavior of the robotic system for optimizing the choice of motors using cost functions for energy consumption, trajectory tracking error, and total weight of the motors. Additionally, another research (30) showcases a method for testing drive-transmission couples suitable for following a defined motion. The analysis considers each pair separately without integrating them into a multi-DOF coupled system. Therefore, while this method enables the individual performance evaluation of drive-transmission couples, it does not provide analysis for situations where such pairs are joined together through a multi-DOF system. This highlights the need for comprehensive approaches that account for the dynamic interactions between coupled components within a multi-DOF robotic system.

Evaluating the performance of interconnected systems with multiple drives and links requires simulation tools. Unfortunately, current tools often lack the capability to simulate entire robotic systems with integrated drive joints, forcing developers to physically test and validate certain components. This gap arises because existing robot simulators typically allow designers to only simulate idealized systems or systems based on various assumptions (31, 32). Modeling complex systems with readily available hardware is crucial. For example, when designing a speed reduction mechanism, engineers typically rely on calculations and simulations to identify suitable gears. However, these simulations might not account for real-world limitations like manufacturability or available parts. A more advantageous approach would be to explore commercially available gear options suitable for lightweight robots, model their performance, and then adapt other system components to achieve the desired speed reduction. This concept

applies beyond gears – it extends to motors, sensors, bearings, motor drivers, and even the robot structure itself.

Industrial robotics has benefited for decades from the use of readily available components to build high-performance manipulators. A counter-intuitive approach, where existing components influence the design process, could be even more desirable. However, this would require easy access to comprehensive simulated models of all commercially available components. Unfortunately, the current landscape does not provide an extensive repertoire of simulated models, and even when such models do exist, there is a notable absence of a *unified and scalable platform* capable of testing the combinations of subcomponents employed in the fabrication of critical elements, such as a single joint within a robotic manipulator or the manipulator as a complete system made up of several subcomponents.

Figure 3 illustrates the comprehensive workflow of the proposed scheme. The end-user provides the initial specifications of the robot, which in the current stage are limited to the degrees-of-freedom, reach of the manipulator, payload carrying capacity, and the maximum workspace speed of the end-effector. These specifications serve as the basis for component selection, leading to the creation of a Computer Aided Design (CAD) model of the robot. This CAD model is subsequently imported into the Simulink environment, where it is subjected to predefined trajectories. Based on the evaluation of position, velocity, and torque tracking performance, frameless BLDC motors and/or strain wave gears may be substituted, if necessary. Once satisfactory performance is achieved in the simulation, that is the robotic manipulator successfully follows the reference trajectories with correlation coefficient greater than 0.95, the robot is fabricated and assembled utilizing the commercially available components identified during the simulation phase. To complete the entire process, a comparative analysis is performed, contrasting the operational performance of the manufactured robot with that of the simulated robot. This research introduces a novel design and development framework for lightweight robots. However, opportunities remain for further optimization of each subcomponent, which will be a focus of future research.

Figure 3: Comprehensive workflow of the proposed scheme.

Methodology for Implementation of the Proposed Scheme

Building a simulation model that captures all aspects of a real-world Integrated Direct Drive Joint (IDJ) is a complex task. However, the frameless BLDC motor and the gearing system emerge as the critical components influencing the IDJ's behavior. Therefore, detailed models were created for these elements, while less complex representations sufficed for smaller components like position encoders and bearings. This prioritization is justified because the BLDC motor and gearing system dominate the IDJ's inertia and electrical power consumption.

Consequently, these two key components primarily define the IDJ's electrical and mechanical characteristics.

The development of models for the involved subcomponents necessitates consideration of several aspects. For instance, a motor requires modeling of both its electrical and mechanical attributes, whereas a gearing system pertains solely to mechanical aspects. However, different types of gearing systems exhibit distinct behaviors. For instance, planetary gear systems offer limited internal gear flexibility but have measurable backlash. In contrast, strain wave gears introduce flexibility through their flex spline and eliminate backlash. Backlash and rigidity are just two key differences between these gear types. Therefore, a comprehensive gear system model requires considering and optimizing various parameters based on specific needs. However, to simplify the process, focusing solely on modeling commercially available components' parameters is a practical approach, without concerning about the validity and optimization of such parameters. Consequently, developers can expedite the process by leveraging existing hardware components and utilizing their models within the simulation environment, effectively bridging the gap between simulation and actual hardware.

The subsequent discussion elaborates on the potential components that an IDJ may encompass. However, it is worth noting that certain components may be omitted in practice without compromising performance, albeit at the expense of non-performance-related features. Moreover, the simulated models utilize parameters sourced from component datasheets, which are typically freely available on the manufacturer's website.

Frameless BLDC Motor Model

"Significant research already exists on simulation models for various motors, including BLDC motors. Since this study aims to find an effective combination of motor models, strain wave gears, and simplified models for other components, there's no need to develop entirely new models from scratch. Therefore, we leveraged standard BLDC motor models e.g. AC7 block of Simulink MATLAB, and adapted them to create models for specific commercially available frameless BLDC motors.".

In addition to the BLDC motor, the drive system used to operate it plays a crucial role. The choice of drive system determines the required feedback and the level of control it can exert over the motor's motion. Typically, commercially available BLDC motor drives implement speed control through stator current control, employing a position sensor such as a rotary encoder or a hall sensor. The generalized mechanical system dynamics are represented by:

$$T_e = J \frac{d}{dt}\omega_r + F\omega_r + T_m \qquad (2)$$

Where T_e represents the electromagnetic torque, J the inertia, ω_r the rotor speed, F the viscous friction, and T_m the load torque. These kinds of drives have an inner current loop that will take in the commanded torque and utilize the torque constant of the motor to generate suitable

stator phase currents. Whereas the outer speed control loop takes in the load or applied torque and reference speed to figure out the electromagnetic torque as per the mechanical system's dynamic equation. Though, the Sliding Mode Control (SMC) is registered as one of the robust model free control but they are complex in nature and the accompanied chattering limits its use in sensitive applications (33-34). Thus, commercial motor drives implement Proportional-Integral (PI), Proportional-Derivative (PD) or Proportional-Integral-Derivative (PID) controllers, due to their simple analog/digital implementation, for maintaining the desired speed-torque characteristics. These controllers are pre-tuned or may easily be tuned using the manufacturer's defined method and provided scheme. Fig. 4 shows the block diagram of the overall control scheme for commercially available motor drives.

Furthermore, commercial motor drives implement Proportional-Integral (PI), Proportional-Derivative (PD) or Proportional-Integral-Derivative (PID) controller for maintaining the desired speed-torque characteristics. These controllers are pre-tuned or may easily be tuned using the manufacturer's defined method and provided scheme. Fig. 4 shows the block diagram of the overall control scheme for commercially available motor drives.

Figure 4: General control scheme implementing closed loop control at all three stages, that is, torque (current), velocity, and position.

This study employed BLDC motor controllers with PI controllers at all three levels: the current loop (innermost), velocity loop, and position loop (outermost). The integrator in PI controllers eliminates steady-state errors, which is particularly beneficial for lightweight robotic manipulators. These manipulators often experience noisy signals due to weak sensor signals being close to powerful BLDC motors. Additionally, all commercially available hardware motor controllers used in this study relied solely on PI control, further supporting our choice. The PI parameters within these hardware controllers were adjusted according to the manufacturer's recommendations. and the trajectory following of a certain actuator before and after the tuning is shown in the hardware experimentation section, while the simulation-based motor controllers were autotuned using the damping factor and desired response time, as outlined in (35). The damping factor is defined as follows:

$$\zeta = \frac{\omega_n K_p}{2K_i} \quad (3)$$

If $\zeta < 0.69$, then natural frequency is defined as:

$$\omega_n = \frac{-1}{\zeta \times T_{nl}} \log(0.05 \times \sqrt{1-\zeta^2}) \quad (4)$$

Where if $\zeta \geq 0.69$, then natural frequency is defined as:

$$\omega_n = \frac{0.9257}{T_{nl}} e^{1.6341 \times \zeta} \quad (5)$$

Strain Wave Gear Model

In this research, the modeling of strain wave gears encompasses several crucial parameters that can be sourced from either the manufacturer's datasheet or the CAD model. These include weight, moment and product of inertia, stiffness, damping constant, nominal torque, and nominal speed. These parameters form the foundational framework for the accurate representation of strain wave gears within the simulation environment, ensuring a comprehensive and precise analysis of their performance characteristics.

While most parameters can be directly extracted, the moment and product of inertia are obtained from the manufacturer-provided CAD model. The damping constant is influenced not only by the grease used in the gearing system but also by the friction in the bearings and overall component alignment. Therefore, an empirical determination of the damping constant was performed for one of the manufactured joints to account for manufacturing imperfections and friction. Standardizing the manufacturing process, materials, assembly processes, strain wave gear grease, and bearings will improve the approximation of the damping constant. The determined damping constant was incorporated into the simulated model, present in the component library, to capture its effects.

Robot Structure

We modeled the robot structure with SolidWorks 2021. All necessary parts were meticulously modeled and analyzed for validity. The Integrated Direct Drive Joint (IDJ) was constructed using supplier-provided CAD models of its components: the frameless BLDC motor, strain wave gear, optical encoder, bearings, and the joint housing that encases them. Additionally, the links and connection flanges were modeled and subjected to stress-strain analysis to ensure the model's validity under worst-case loading conditions. Aluminum 6061-T6, known for its strength-to-weight ratio, was chosen as the material for the links and joint housings. Meanwhile, the connection flanges utilized high-strength Alloy Steel with a yield strength of $6.2\text{x}10^8$ N/m^2. The factor of safety of the designed Aluminum links is at least 2.4, while for flanges its 4.3. Fig. 5 shows stress analysis figure of one of the links being subjected to a maximum of $1.158\text{x}10^8$ N/m^2 stress, while Aluminum 6061-T6 has yield strength of $2.75\text{x}10^8$ N/m^2, hence achieve 2.4 factor of safety. These values of the factor of safety ensure that the structure can easily withstand higher loading, hence increasing the structural integrity and life of the manipulator. Some covers which were not bearing any load, were later 3D printed, and were excluded from the analysis.

Figure 5: Stress Analysis of one of the links showing maximum stress being applied on it in worst case.

The CAD model of the robot's structure generated in SolidWorks was imported into MATLAB's environment for simulation. The CAD model considered the following parameters:

- Robot configuration

- Link weights
- Link moment and product of inertias
- Link lengths

Fig. 6 illustrates one of the designed links and its integration with the joints to form the complete CAD model of the 5 DOF robot.

Figure 6: Exploded view of the 5 DOF robot showing Links, Joints, Connection flanges, and 3D printed covers.

The integrated drive joint of the robotic manipulator includes additional subcomponents with minimal weight and moments of inertia. These components, listed below, can be grouped and represented as a single model by combining their net weight and inertia. Their individual influence on the overall dynamics is negligible due to their small size.

Other components

Several other components, like miniaturized circuit boards, position sensors, bearings, torque sensors, brakes, and housings, might be included in an integrated drive joint (36). These components typically have significantly lower weights and moments of inertia compared to the BLDC motor, strain wave gear, and robot structure. To simplify the analysis, these components are grouped together as a single unit. The next section details the considerations for modeling and manufacturing the integrated drive joint.

Simulation Model Flow

The direct drive joint model was built by individually modeling and assembling commercially available subcomponents. These subcomponents include a frameless BLDC motor, strain wave gear, incremental position encoder, hollow shaft, bearings, joint housing, and various minor parts. The electrical behavior of the BLDC motor was modeled in Simulink MATLAB using the Brushless DC Motor Drive (AC7) block, which simulates a standard current-controlled drive system for BLDC motors with closed-loop speed control. The mechanical aspects of the motor and strain wave gear were modeled within SolidWorks. The modeled parts make up the component library from which suitable parts are to be selected to make up the desired robotic manipulator. Table 2 presents the parameters used to model the frameless BLDC motor, extracted from commercially available datasheets. Furthermore, to account for potential manufacturing inaccuracies, up to 10% random variations were introduced in the weight, inertia, and stiffness parameters of the simulated direct drive joint and robot's structure. These variations compensate for possible deviations that may arise during the actual manufacturing process, such as material and machining differences and assembly imperfections. Additionally, an empirically determined damping constant was incorporated to model frictional forces. A test joint was manufactured to evaluate manufacturing imperfections and determine frictional losses, which were then included in the simulated models present in the developed library. All joints of

the 5-DOF manipulator were configured with a damping constant of 0.035 Nm/rad/s. Fig. 7 illustrates a snapshot of a test joint.

Figure 7: IDJ Testing Setup

The developed simulator includes eight different frameless BLDC motors from T-Motor and Maxon, allowing for modeling of various motors without manufacturer dependence.

Table 2: List of BLDC Motor Parameters that were used for modeling the commercially available motors. Sample of three different BLDC Motors are shown.

Sr. #	Parameter	Unit	TMotor RI50	TMotor RI80	Maxon 588849
1	Resistance	Ohm	0.673	0.196	0.109
2	Inductance	mH	0.474	0.364	0.0665
3	BEMF Flat Area	rad	1.197	1.197	0.761
4	Torque Constant	mNm/A	99	127.3	80.7
5	Speed Constant	rad/s/V	10.11	7.85	12.36
6	Robot Inertia	$g.cm^2$	3500	6000	5300
7	Poles	-	7	7	11
8	Nominal Torque	mNm	500	1450	1010

Similarly, several parameters of strain wave gear were used to model its effect in the direct drive joint, out of which the gear ratio is defined in the motor block. Apart from gear ratio, the weight, moment and product of inertias, stiffness, nominal torque, and nominal speed parameters were defined and used in different blocks. Furthermore, the simulated direct drive joint model includes the weights and inertias of position encoder, bearings, joint housing, and the hollow shaft through which the power and communication wires are routed.

Fig. 8 shows the section view of the CAD model of the simulated direct drive joint.

Figure 8: Section view of the CAD model of the assembled Integrated Drive Joint

Later, the simulated integrated drive joint was installed in the simulated robot structure, and the manipulator was driven on a defined trajectory. The position, velocity, and torque requirement graphs were plotted to establish the suitability of the simulated direct drive motor joint.

Manipulator Model

To evaluate the simulated integrated drive joint, a robotic manipulator with specifications defined in the Simulation Experimentation section was simulated. The development of the manipulator's model considered critical parameters, including robot configuration, link weights, link moment, product of inertias, and link lengths. These considerations were pivotal in ensuring

the accuracy and effectiveness of the simulated manipulator's performance under various conditions.

Table 3 presents the parameters of the robot links and joints used to generate the simulation model. These parameters were obtained from the CAD model, which considers the commercially available material, its properties, and the machining processes involved. While importing the model from SolidWorks 2021 to the Simulink environment, the dimensions and shape of the links were preserved. However, the shape and size of the joints were approximated by a point mass while maintaining all other parameters.

Table 3: List of Parameters used for modeling the structure of the robot in Simulink Environment.

Sr. #	Manipulator Link		Manipulator Joint	
	**Parameter**	**Unit**	**Parameter**	**Unit**
1	Size and Shape	mm	Point Mass	-
1	Mass	kg	Mass	kg
2	Center of Mass	mm	Center of Mass	mm
3	Moments of Inertia	$g.cm^2$	Combined Moments of Inertia	$g.cm^2$
4	Products of Inertia	$g.cm^2$	Combined Products of Inertia	$g.cm^2$

Individual links of the robotic arm were imported into the Multibody environment and were assembled in the defined configuration to build the complete structure. Fig. 9 shows the Simulink model for building the robotic arm in MATLAB.

Figure 9: Simulink SimScape Multibody model for building 5 DOF Robotic Arm

Section III

Simulation Experimentation

Using the steps outlined in Fig. 3, the experimentation was initiated by specifying the initial specifications of the desired robot. The DOF were chosen to be 5, reach of about $1.1\ m$, with a payload capacity of $3\ kg$ and as we are only focusing on developing lightweight robots then maximum speed of the end-effector was confined to $1\ m/s$. This value of speed ensures that the designed robot doesn't breach the safety guidelines laid down in ISO/TS-15066 (37). In the experimentation phase, direct drive joints featuring various commercially available frameless BLDC motors were integrated with the 5-DOF manipulator model made of manually selected robot links from the produced library. Trapezoidal velocity trajectories were generated as reference trajectories for different scenarios, and the simulated model was driven on these trajectories to evaluate the performance of the direct drive joints. Fig. 10 provides a snapshot of the simulation running on a specific trajectory.

Figure 10: Snapshot of simulation of 5 DOF manipulator on a given trajectory.

The simulation was conducted using the finalized parameters and SimScape Multibody model. The SimScape Multibody simulates system dynamics using a range of solvers, which can be variable or fixed steps. As we are going to test the physically manufactured robot on EtherCAT protocol, we used a fixed step solver with step-size of $0.1\ ms$. The EtherCAT protocol runs at a sampling time of $1\ ms$, that is why we had to run the simulation at an even smaller time step. The type fo the solver was left to Simulink by allowing it to automatically select the solver type. The results aimed to verify if the simulated joints could precisely track the desired reference trajectories. For a robotic manipulator with interconnected joints and links, evaluating the coupled dynamics of the entire structure provided a more meaningful assessment than analyzing individual joint performance. In industrial settings, robotic manipulators encounter various disturbances from environmental interactions, unmodeled dynamics, and gravitational effects (38). These disturbances are addressed at the system level. Figure 11 illustrates the system-level Simulink model used to simulate the complete robotic system.

Figure 11: System level Simulink Model for simulating the robotic manipulator and its joints.

The system model shown in Fig. 11 outlines the simulation flow. A reference trajectory was generated in joint space and applied to the Multibody model of the robot. The model followed the trajectory, while position, velocity, and torque sensors captured corresponding signals. The sensed positions and torques were then used to control the BLDC motors through a PI-based control system. Joints that were capable of accurately following the desired position and torque profile exhibited minimal deviation between the output and reference signals. To quantitatively assess the similarity between the reference and simulated data, Pearson's Correlation Coefficient was calculated using the provided equation. Additionally, visual comparisons were also conducted.

$$r = \frac{\sum(x_i-\bar{x})(y_i-\bar{y})}{\sqrt{\sum(x_i-\bar{x})^2 \sum(y_i-\bar{y})^2}} \quad (5)$$

Where r = Pearson's Correlation Coefficient, x = value of the first variable, $\bar{x}$ = mean value of the first variable, y = value of the second variable, and $\bar{y}$ = mean value of the second variable.

The Pearson's Correlation Coefficient remains a crucial metric to assess the degree of similarity between the reference and simulated torque profiles. It serves as an indicator of the suitability of the direct drive joint at its installed position. A low correlation coefficient value suggests an unsuitable joint, while a value closer to one indicates that the joint is well-suited for its installed position within the robot. The correlation coefficient is specifically calculated for the torque profile, as the correlation coefficients of the position and velocity profiles are dependent

on the coefficient of the torque profile. Fig. 12 demonstrates the response of an underpowered joint, highlighting the significance of adequate power for joint performance.

Figure 12: (a) Position, (b) Velocity, (c) Torque Reference vs Simulation Data of Joint 2 when an underpowered joint was installed at this location.

The underpowered joint may cause significant deviations in position and velocity responses from the reference trajectory due to insufficient torque. The generated torque fell short of desired value, reaching around 50 Nm compared to the required 70 Nm, leading to a failure in following the trajectory. The correlation coefficient of 0.2593 quantitatively indicated a mismatch between the required and generated torque. To address this issue, we iterated the simulation using different integrated drive joints, gradually moving from low to high power joints. This IDJ selection method is currently manual with the aim of being automated in future. Currently the criteria for selection were to minimize the total weight of the joints and achieve enough torque capability to drive the robot on the reference trajectory with a correlation coefficient greater than 0.95. The selection process began with the last joint and moved backward, considering the impact of later joints on the earlier ones. This selection methodology helps identify optimal joints for each position. Intelligent selection methods will be implemented as the library of simulated integrated drive joints expands, currently including frameless BLDC motors and strain wave gears from Maxon, TMotor, and Laifual Drive. Furthermore, we successfully resolved the underpowered joint issue by substituting it with the suitable direct drive joints and tested the robot on a pick-and-place trajectory. Trajectories for position, velocity, and torque were obtained for all five joints of the 5-DOF robot. Through visual inspection, point-by-point comparison, and Pearson's correlation coefficient analysis, we confirmed that the joint trajectories of the simulated model closely followed the reference trajectories, surpassing the defined acceptable level of correlation coefficients greater than 0.95.

For the pick and place application, the reference versus simulated data for all the joints is shown in Figs. 13 to 17.

Figure 13: Reference vs Simulation Data of Joint 1's (a) Position, (b) Velocity, and (c) Torque.

Figure 14: Reference vs Simulation Data of Joint 2's (a) Position, (b) Velocity, and (c) Torque.

Figure 15: Reference vs Simulation Data of Joint 3's (a) Position, (b) Velocity, and (c) Torque.

Figure 16: Reference vs Simulation Data of Joint 4's (a) Position, (b) Velocity, and (c) Torque.

Figure 17: Reference vs Simulation Data of Joint 5's (a) Position, (b) Velocity, and (c) Torque.

The simulation results demonstrate that the simulated integrated drive joint successfully produces the reference trajectory in all cases. The torque graph reveals a disparity between the required and simulated torque, but this difference does not result in noticeable deviations in the position or velocity trajectories due to the robustness of the PI controller. Additionally, Table 4 provides the Pearson's Correlation Coefficients for all joints, quantifying the degree of match between the reference and simulated trajectories.

Table 4: Pearson's Correlation Coefficient between Reference and Simulated Torque Profiles of all joints for Pick and Place Trajectory.

Parameter	Joint 1	Joint 2	Joint 3	Joint 4	Joint 5
Correlation Coefficient	0.9969	0.9984	0.9982	0.9992	0.9963

The high correlation coefficients obtained in the simulation experiments highlight the effectiveness of the simulation tool and the chosen motor and harmonic drive combinations in achieving accurate and reliable robot motion control. These results demonstrate that the simulated robot performance closely aligns with the intended reference trajectories, which is crucial for ensuring precise and consistent robot motion.

Several sources of noise and disturbances including EMI, sensor (encoder) noise, input and output disturbances can cause the manipulator to deviate from an ideal trajectory in real-life. We analyzed how adequately our PI controller would reject sources of noise disturbances in real-life by lumping these at the output of the speed controller, in the form of a single noise source. Specifically, this is an additive white gaussian noise with unit variance, resulting in a signal-to-noise ratio (SNR) of 15 dB, which is in close agreement with (Fig. 22) noise levels observed in real experiments. Fig. 18 depicts the responses of position, velocity, and torque tracking for one of the joints, joint 3, subject to noise/disturbance.

Figure 18: Reference vs Simulation data of Joint 3 after introducing disturbance, (a) Position, (b) Velocity, and (c) Torque.

In Fig. 18, although there is distortion in the speed and, consequently, in the torque, the PI controller implemented in the position loop adeptly mitigates the effects of the introduced disturbance. This reinforces the robustness of the controller.

This finding carries significant implications for practical applications, as it suggests that physically manufactured robots using the same integrated drive joints and control system can potentially achieve similar performance. The subsequent section presents the results of fabricating the robot based on the integrated drive joints recommended by the simulation studies.

Section IV

Hardware Experimentation

Based on the simulation results, integrated drive joints were built using the recommended commercially available components. To comply with modern industrial standards, Nanotec® BLDC motor controllers with EtherCAT communication capabilities were chosen to drive the BLDC motors. Five such joints were manufactured alongside the manipulator's structure. The complete assembly resulted in a 5-DOF manipulator, pictured in Fig. 19, controlled via EtherCAT-enabled BLDC motor controllers.

Figure 19: Manufactured 5DOF Robot with Integrated Drive Joints performing a pick and place task.

We used Plug & Drive software, provided by Nanotec®, for tuning the PI control loops of the motor controllers. This motor controller, similar to implements the PI control loops on three levels, that is, the inner most on torque, then on velocity, and the outer most on position. The position and velocity following response of one of the motor controllers before and after tuning is shown in Figs, 20 and 21.

Figures 20 and 21: Before and After tuning response of position (left) and Velocity (right) of one of the joints. The x-axis shows time in milliseconds, whereas y-axis shows the position and velocity in motor controller units.

The physical prototype robot was programmed to follow the trajectory used in the simulation, and the corresponding torque, velocity, and position data from the integrated drive joints were collected. The collected data was compared with the simulated data for the pick and place joint space trajectory, as shown in Figs. 22 to 26. It is important to note that the time constant of a geared joint prevents any noticeable movement of the output shaft due to current spikes. However, for experimentation purposes, the torque data was obtained from the current sensor in the motor controller, at a sampling rate of 1000 Hz, and passed through a moving average filter with a window size of 15 samples, approximately equal to 0.015 seconds in time. This moving average filter emulates the damping effect of the geared joint.

Figure 22: Simulated versus Experimental data of Joint 1's (a) Position, (b) Velocity, and (c) Torque.

Figure 23: Simulated versus Experimental data of Joint 2's (a) Position, (b) Velocity, and (c) Torque.

Figure 24: Simulated versus Experimental data of Joint 3's (a) Position, (b) Velocity, and (c) Torque.

Figure 25: Simulated versus Experimental data of Joint 4's (a) Position, (b) Velocity, and (c) Torque.

Figure 26: Simulated versus Experimental data of Joint 5's (a) Position, (b) Velocity, and (c) Torque.

Once again, visual and Pearson's correlation coefficient were used to establish the similarity of the trajectories coming from the simulated model and the actual robot. Table 5 lists the Pearson's Correlation Coefficient for torque profiles of all joints.

Table 5: Pearson's Correlation Coefficient between Simulated and Actual Torque Profiles of all joints for Pick and Place Trajectory.

Parameter	Joint 1	Joint 2	Joint 3	Joint 4	Joint 5
Correlation Coefficient	0.9966	0.9872	0.9882	0.9888	0.9895

Although the correlation coefficients have slightly decreased, they remain within an acceptable range as the position and velocity trajectories are still being accurately followed. However, the mismatches in the torque trajectories require further investigation. The torque impulses observed in the physical prototype come from the current sensing on the motor controller. These impulses are within the current limits of the BLDC motors and do not cause any deviation in the required position trajectory. Additionally, the average torque deviation from the simulated model remains below 5 Nm for most of the time, which is a small percentage of the nominal torque capability of the integrated drive joints. This additional torque compensates for unmodeled dynamics, friction, and manufacturing variations. Simulation tools capable of predicting the robot behavior in an ideal world may fail to account for the performance under real-world imperfections such as friction and manufacturing variations. However, the position and velocity following performance of the robot is still below acceptable level due to the disturbance rejection and noise cancellation properties of the PI controllers used. These results emphasize the importance of considering real-world complexities and the need for ongoing calibration and optimization. We aim to further investigate this problem to minimize the simulation-experiment performance disparity.

Apart from visual comparison and correlation coefficient between the simulated and the actual trajectories, trajectory tracking error was also calculated to further establish the trajectory following capability of the physically developed manipulator. We calculated Root Mean Squared Error (RMSE) for all five joints using equation 6, given below.

$$RMSE = \sqrt{\frac{1}{N}\sum_{i=1}^{N}(x_{ref,i} - x_{measured,i})^2} \quad (7)$$

Table 6 gives the RMSE of joint space position, velocity, and torque trajectories. Position and velocity RMSE signifies the position and time tracking efficiency of the developed robot, whereas torque RMSE once again suggests that the deviation in torque is a result of unmodeled dynamics. The maximum RMSE of torque is for joint 2, which is 5.4244 Nm, and if considered

along with Fig. 23, then this is around 10% of the total torque output for the desired trajectory. Moreover, the IDJ installed at joint 2 can produce torque up to 145 Nm, therefore, if the RMSE is seen in comparison to the maximum torque capability of the joint then it is only around 3.74%.

Table 6: Root Mean Squared Error of joint space position (rad), velocity (rad/s), and torque (Nm) trajectories calculated using the simulated and measured trajectories of physically developed 5 DOF manipulator.

Trajectory	Joint 1	Joint 2	Joint 3	Joint 4	Joint 5
Position (rad)	0.0018	0.0086	0.0042	0.0091	0.0046
Velocity (rad/s)	0.0062	0.0223	0.0134	0.0238	0.0125
Torque (Nm)	1.8585	5.4244	2.3571	3.7471	1.5893

Furthermore, the repeatability of the developed 5-DOF robot prototype was calculated as per the procedure described in ISO 9283:1998 (39), defined specifically for industrial robots. A dial gauge having positional resolution of 0.01mm was used to measure the position of the end-effector of the robot. Five different positions were commanded to the robot and 100 iterations were carried out for each position to generate the position data. To calculate the positioning repeatability the following calculations were carried out.

$$l_i = \sqrt{(x_i - \bar{x})^2} \qquad (8)$$

$$\bar{l} = \frac{1}{n}\sum_{i=1}^{n} l_i \qquad (9)$$

Where 'x_i' is the measured value in i^th iteration, '$\bar{x}$' is the mean position of all iterations, 'n' is the number of iterations, and '$\bar{l}$' is then the mean positional repeatability according to ANSI/RIA R15.05 standard (40). Moreover, the standard deviation 'S' is then calculated as:

$$S = \sqrt{\sum_{i=1}^{n} \frac{(l_i - \bar{l})^2}{n-1}} \qquad (10)$$

Lastly, the positional repeatability, 'Rp', is defined and calculated as

$$Rp = \bar{l} + 3S \qquad (11)$$

Table 7 shows the Standard Deviation and the Positional Repeatability for each position. The worst value of the repeatability achieved in this experimentation is claimed as the repeatability of the developed robot prototype. Comparison with commercially available lightweight robots establishes that this value of repeatability is acceptable for robotic manipulators. The worst-case value of repeatability, 0.0503 mm, of our developed robotic manipulator is equivalent to commercially available lightweight robot Techman Robot TM5 (41).

Table 7: Standard Deviation and Repeatability of 5-DOF robot measured and calculated as suggested in ISO 9283:1998 for five different positions. All values are in mm.

Robot	Position 1		Position 2		Position 3		Position 4		Position 5	
	S	Rp	S	Rp	S	Rp	S	Rp	S	Rp
5-DOF	0.0018	0.0080	0.0039	0.0163	0.0062	0.0260	0.0107	0.0503	0.0030	0.0138

Overall, the developed simulation tool demonstrates its potential for robot design and development, particularly in the selection of integrated drive joint subcomponents. Using the proposed scheme, the authors along with their research team have also developed a 6-DOF robot manipulator, with 1m reach and 4Kg payload capacity, shown in Fig. 27, is in the testing phase. Fig. 27 shows the configuration and hardware of the new manipulator.

Figure 27: Newly developed 6 DOF Robot's FBD (Left) and its hardware (Right).

Section V

Conclusion and Future Work

This research underscores the benefits of a component-driven approach for designing and building lightweight robotic manipulators. By leveraging models of commercially available components within the design process, the gap between simulations and real-world systems narrows significantly. This can lead to eliminating or streamlining various steps in the development process, resulting in faster and more efficient creation of lightweight robotic manipulators.

The presented framework demonstrates the effectiveness of this approach through the modeling and analysis of a physical prototype of a 5-DOF lightweight robotic manipulator. Notably, the experimental evaluation of the developed robotic system yielded impressive results. The positional repeatability, assessed in accordance with ISO 9283:1998, demonstrated a repeatability of 0.05 mm, a performance level comparable to the Techman Robot TM5, a commercially available lightweight robot.

Following hardware experiments, where the same integrated drive joints and robot structure were manufactured and tested along predetermined trajectories, results showed close alignment between the simulated model and the actual position and velocity readings. However, a noticeable difference was observed in the required torque compared to the simulation. This discrepancy can be attributed to factors not included in the model, such as friction from the strain wave gears and potential misalignments introduced during assembly. Encouragingly, the magnitude of this torque difference remained minor compared to the joints' nominal torque capacity.

Our study presents a simulation tool and framework with the potential to significantly transform the robot manufacturing industry. By allowing developers in educational research, robotics startups, and related fields to simulate manipulators using real-world component models, it offers substantial savings in time, effort, and investment. This is particularly valuable in today's fast-paced and resource-constrained environment.

Future work will focus on several key areas: refining the simulation tool for even greater accuracy, expanding the library of simulated models to encompass a wider range of components from diverse manufacturers, and tackling the complexities of unmodeled dynamics. By continuously enhancing the framework's accuracy, reliability, and user-friendliness, we aim to solidify its position as a transformative tool within the robot manufacturing industry.

Acknowledgements

This research is supported by the Higher Education Commission (HEC) Pakistan under the project 'The establishment of National Center for Robotics and Automation (NCRA). We would also like to thank Dr. Ali Raza, Principal Investigator (PI) of Human Centered Robotics Lab UET Lahore for his unwavering support and guidance.

References

1. Akundi A, Euresti D, Luna S, Ankobiah W, Lopes A, Edinbarough I. State of Industry 5.0—Analysis and identification of current research trends. Applied System Innovation. 2022;5(1):27.
2. Hirzinger G, Albu-Schaffer A, Hahnle M, Schaefer I, Sporer N, editors. On a new generation of torque controlled light-weight robots. Proceedings 2001 ICRA IEEE International Conference on Robotics and Automation (Cat No 01CH37164); 2001: IEEE.
3. Popić S, Miloradović B. Light weight robot arms-an overview. INFOTEH-JAHORINA. 2015;14.
4. Balding S, Gning A, Cheng Y, Iqbal J. Information Rich Voxel Grid for Use in Heterogeneous Multi-Agent Robotics. Applied Sciences. 2023;13(8):5065.
5. Bilal M, Rizwan M, Maqbool HF, Ahsan M, Raza A. Design optimization of powered ankle prosthesis to reduce peak power requirement. Science Progress. 2022;105(3):00368504221117895.
6. Naeem A, Rizwan M, Maqbool HF, Ahsan M, Raza A, Abouhossein A, et al. Virtual constraint control of Knee-Ankle prosthesis using an improved estimate of the thigh phase-variable. Biomedical Signal Processing and Control. 2022;73:103366.
7. Calvo R, Gil P. Evaluation of collaborative robot sustainable integration in manufacturing assembly by using process time savings. Materials. 2022;15(2):611.
8. Gheorghe G, editor Concepts and mechatronics and cyber-mixmechatronics constructions, integrated in COBOT type technology platform for intelligent industry (4.0). Proceedings of the International Conference of Mechatronics and Cyber-MixMechatronics–2019 3; 2020: Springer.
9. Timms MJ. Letting artificial intelligence in education out of the box: educational cobots and smart classrooms. International Journal of Artificial Intelligence in Education. 2016;26:701-12.
10. Djuric AM, Urbanic R, Rickli J. A framework for collaborative robot (CoBot) integration in advanced manufacturing systems. SAE International Journal of Materials and Manufacturing. 2016;9(2):457-64.
11. Chung JC, Hwang T-S, Wu C-T, Jiang Y, Wang J-Y, Bai Y, et al. Framework for integrated mechanical design automation. Computer-aided design. 2000;32(5-6):355-65.

12. SANCHEZ S. Career Karma2022. Available from: https://careerkarma.com/blog/how-to-build-your-own-robot/#:~:text=How%20Long%20Does%20It%20Take,mechanical%20and%20electrical%20assembly%20expertise.
13. Ruishu Z, Chang Z, Weigang Z, editors. The status and development of industrial robots. IOP Conference Series: Materials Science and Engineering; 2018: IOP Publishing.
14. BRACJUNOVÁ VL. Robots take the robot out of the human: Soitron Group; 2019 [Available from: https://www.soitron.com/robots-take-the-robot-out-of-the-human/#:~:text=Creating%20a%20new%20robot%20from,part%20of%20the%20development%20process.
15. Imran K. Y, Farrukh W, Qudrat K, A. I. Bhatti, R. Ullah and R. Akmeliawati. (2021). A Linear Parameter Varying Strategy Based Integral Sliding Mode Control Protocol Development and Its Implementation on Ball and Beam Balancer. IEEE Access, 74437-74445.
16. Hirose S, Arikawa K. Coupled and decoupled actuation of robotic mechanisms. Advanced Robotics. 2001;15(2):125-38.
17. Tarkian M, O¨ lvander J, Feng X, Petterson M, editors. Design automation of modular industrial robots. International Design Engineering Technical Conferences and Computers and Information in Engineering Conference; 2009.
18. Covell DM. Top-Down & Bottom-Up Approaches to Robot Design: West Virginia University; 2022.
19. Zimmermann M, Königs S, Niemeyer C, Fender J, Zeherbauer C, Vitale R, et al. On the design of large systems subject to uncertainty. Journal of Engineering Design. 2017;28(4):233-54.
20. Sathuluri A, Sureshbabu AV, Frank J, Amm M, Zimmermann M. Computational Systems Design of Low-Cost Lightweight Robots. Robotics. 2023;12(4):91.
21. Walden DD. Systems engineering handbook: A guide for system life cycle processes and activities. (No Title). 2015.
22. Albu-Schäffer A, Haddadin S, Ott C, Stemmer A, Wimböck T, Hirzinger G. The DLR lightweight robot: design and control concepts for robots in human environments. Industrial Robot: an international journal. 2007;34(5):376-85.
23. Bolignari M, Fontana M. Design and experimental characterization of a high performance hydrostatic transmission for robot actuation. Meccanica. 2020;55(5):1169-79.
24. Mo S, Li X, Yang Z-n, Zhou C-p, Gao H-j, Cen G-j, et al. Integrated design of brushless motor drive and control system for robot joints. Journal of Central South University. 2021;28(12):3818-28.
25. Bischoff R, Kurth J, Schreiber G, Koeppe R, Albu-Schäffer A, Beyer A, et al., editors. The KUKA-DLR Lightweight Robot arm-a new reference platform for robotics research and manufacturing. ISR 2010 (41st international symposium on robotics) and ROBOTIK 2010 (6th German conference on robotics); 2010: VDE.
26. Mohanraj D, Aruldavid R, Verma R, Sathyasekar K, Barnawi AB, Chokkalingam B, et al. A Review of BLDC Motor: State of Art, Advanced Control Techniques, and Applications. IEEE Access. 2022.
27. Routh B, editor Design aspects of harmonic drive gear and performance improvement of its by problems identification: A review. AIP conference proceedings; 2018: AIP Publishing LLC.
28. Padilla-Garcia EA, Rodriguez-Angeles A, Resendiz JR, Cruz-Villar CA. Concurrent optimization for selection and control of AC servomotors on the powertrain of industrial robots. IEEE Access. 2018;6:27923-38.
29. Padilla-García EA, Cervantes-Culebro H, Rodriguez-Angeles A, Cruz-Villar CA. Selection/control concurrent optimization of BLDC motors for industrial robots. Plos one. 2023;18(8):e0289717.
30. Cusimano G, Casolo F. An almost comprehensive approach for the choice of motor and transmission in mechatronic applications: Torque peak of the motor. Machines. 2021;9(8):159.
31. Machado TDN, Sousa M, Monteiro S, Bicho E. CoopDynSim: a 3D robotics simulator. 2012.

32. Baizid K, Ćuković S, Iqbal J, Yousnadj A, Chellali R, Meddahi A, et al. IRoSim: Industrial Robotics Simulation Design Planning and Optimization platform based on CAD and knowledgeware technologies. Robotics and Computer-Integrated Manufacturing. 2016;42:121-34.
33. Khan, I., Bhatti, A.I., Arshad, A., Khan, Q. (2016). Robustness and performance parameterization of smooth second order sliding mode control. *International journal of control, automation and systems, 14*, 681–690. doi:https://doi.org/10.1007/s12555-014-0181-6
34. Khan, I. (2016). *On performance based design of smooth sliding mode control.* Islamabad: Higher Education Commission of Pakistan.
35. Bose BK. Modern power electronics and AC drives: Prentice hall Upper Saddle River, NJ; 2002.
36. Gutiérrez S, Zotovic R, Navarro M, Meseguer M. Design and manufacturing of a prototype of a lightweight robot arm. Procedia Manufacturing. 2017;13:283-90.
37. ISO. ISO/TS 15066:2016(en) Robots and robotic devices — Collaborative robots 2016 [Available from: https://www.iso.org/obp/ui/#iso:std:iso:ts:15066:ed-1:v1:en.
38. San-Miguel A, Puig V, Alenyà G. Disturbance observer-based LPV feedback control of a N-DoF robotic manipulator including compliance through gain shifting. Control Engineering Practice. 2021;115:104887.
39. Standardization TIOf. ISO 9283:1998(en) Manipulating industrial robots — Performance criteria and related test methods. 1998.
40. Institute ANS. American National Standard for Industrial Robots and Robot Systems - Point-to-Point and Static Performance Characteristics – Evaluation. ANSI/RIA R1505-1-1990 (R1999)1999.
41. TECHMANROBOTINC. TM5 - 900 2019 [Available from: https://www.tm-robot.com/en/tm5-900/.

Click here to access/download;Figure;Fig1.eps

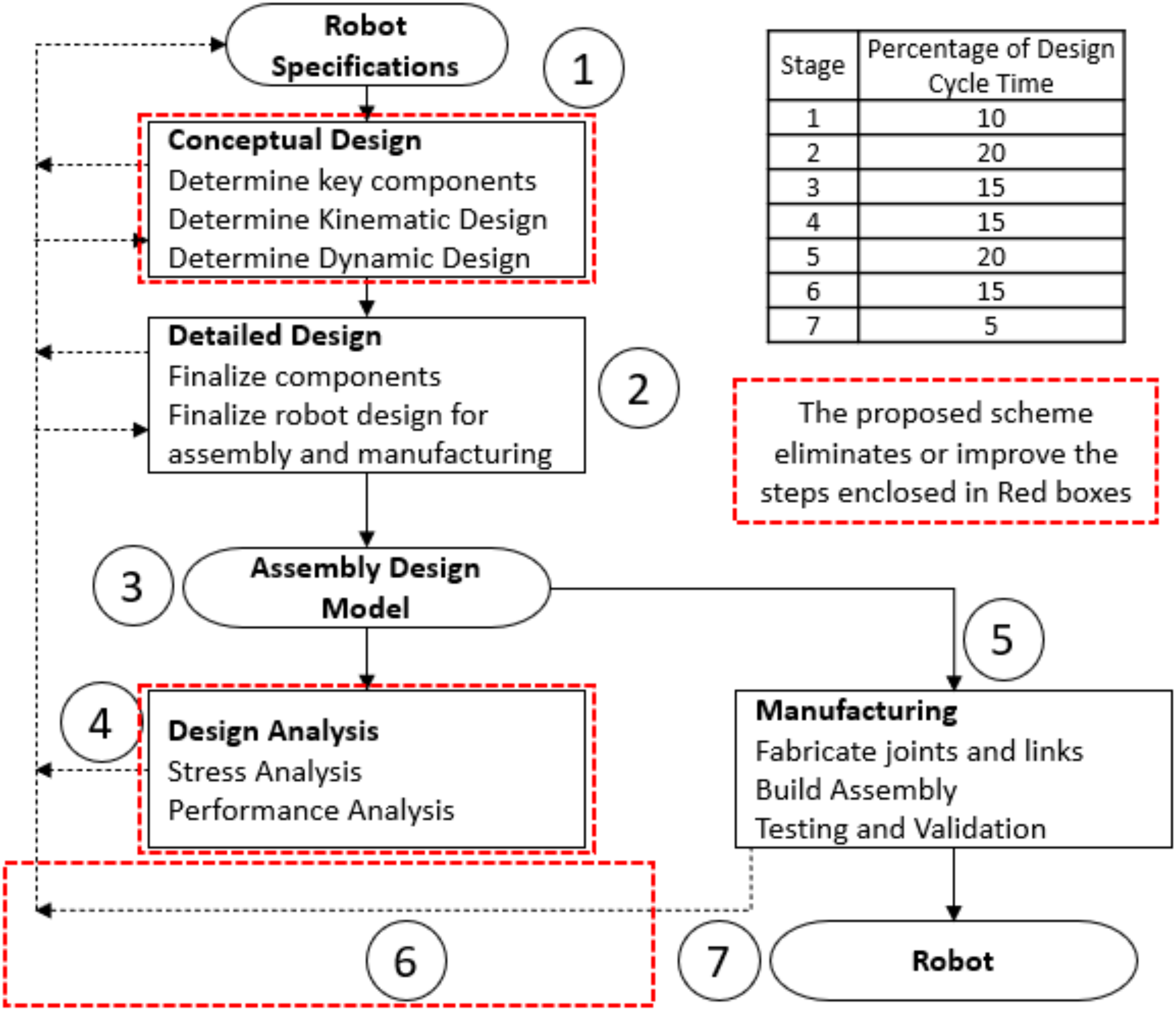

Stage	Percentage of Design Cycle Time
1	10
2	20
3	15
4	15
5	20
6	15
7	5

Click here to access/download;Figure;Fig2.eps

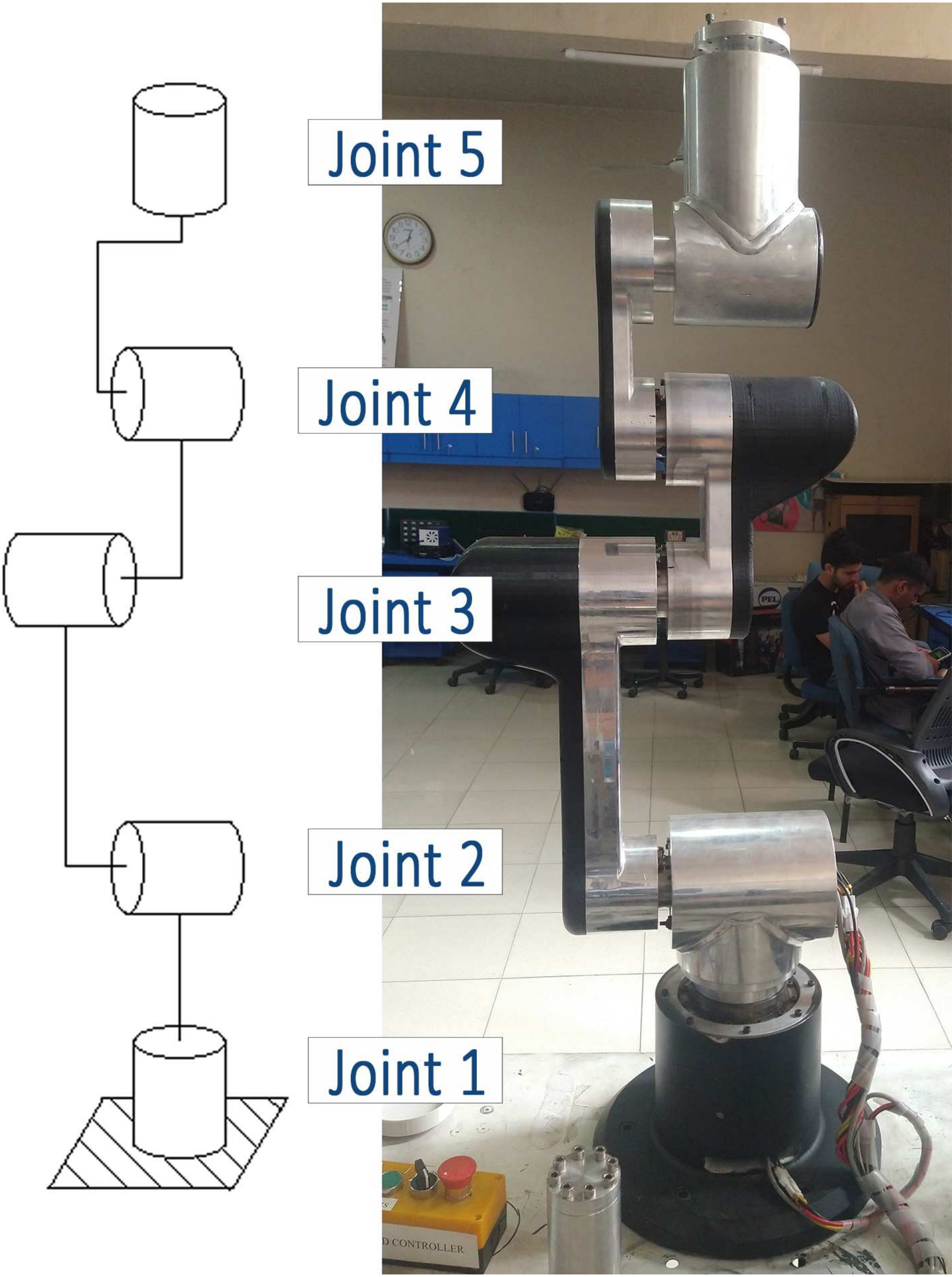

Click here to access/download;Figure;Fig3.eps

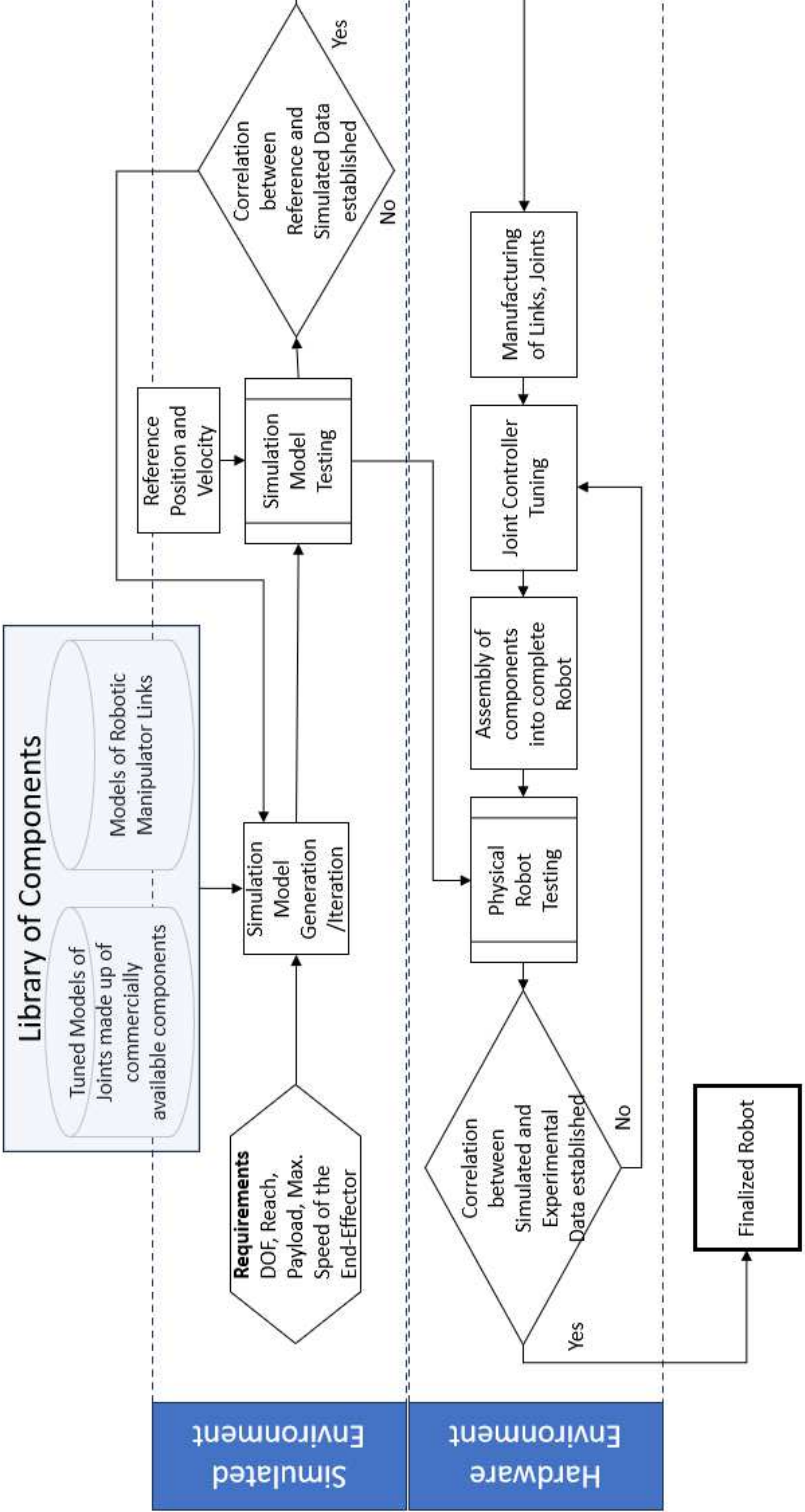

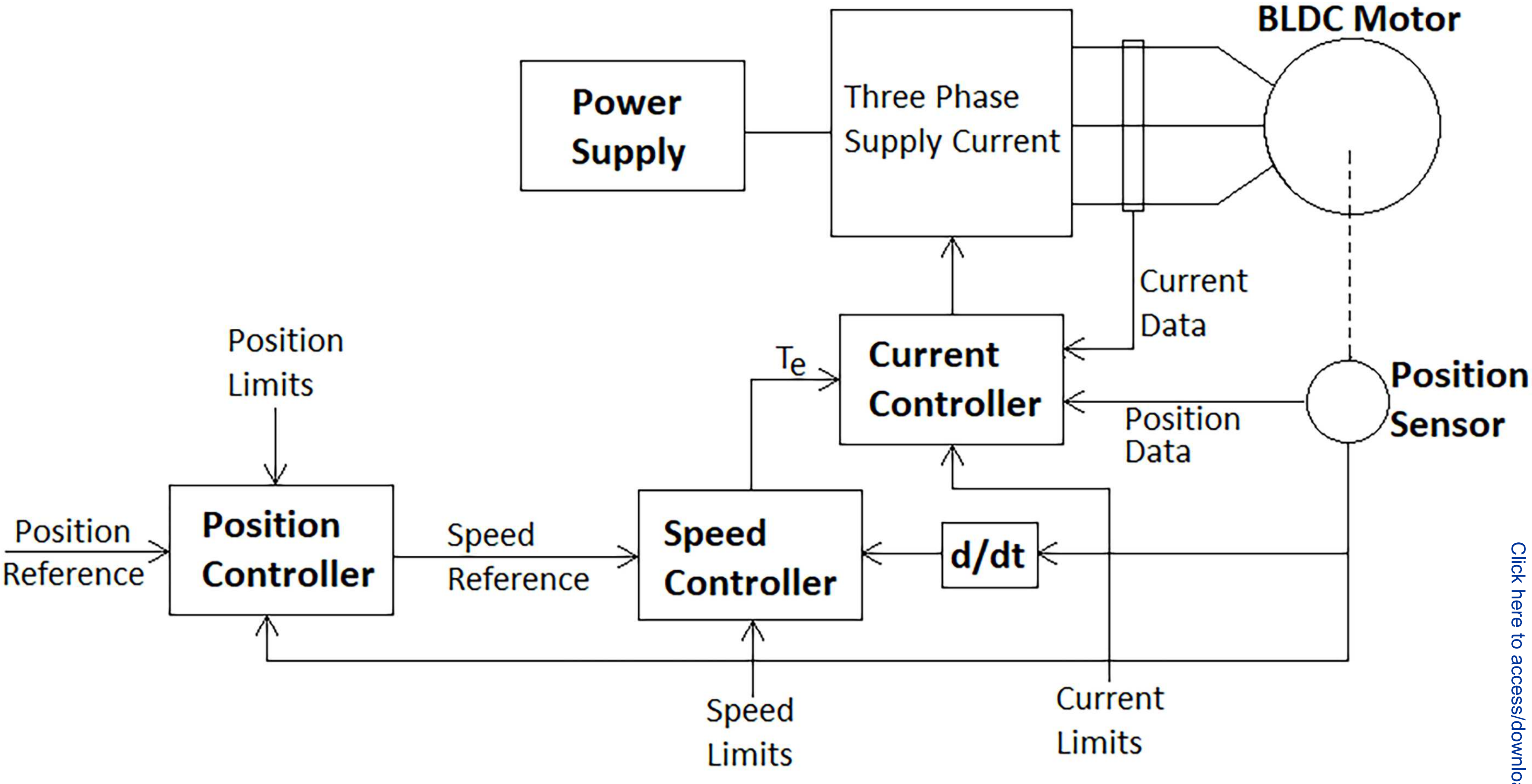

Click here to access/download;Figure;Fig4.eps

Click here to access/download;Figure;Fig5.eps

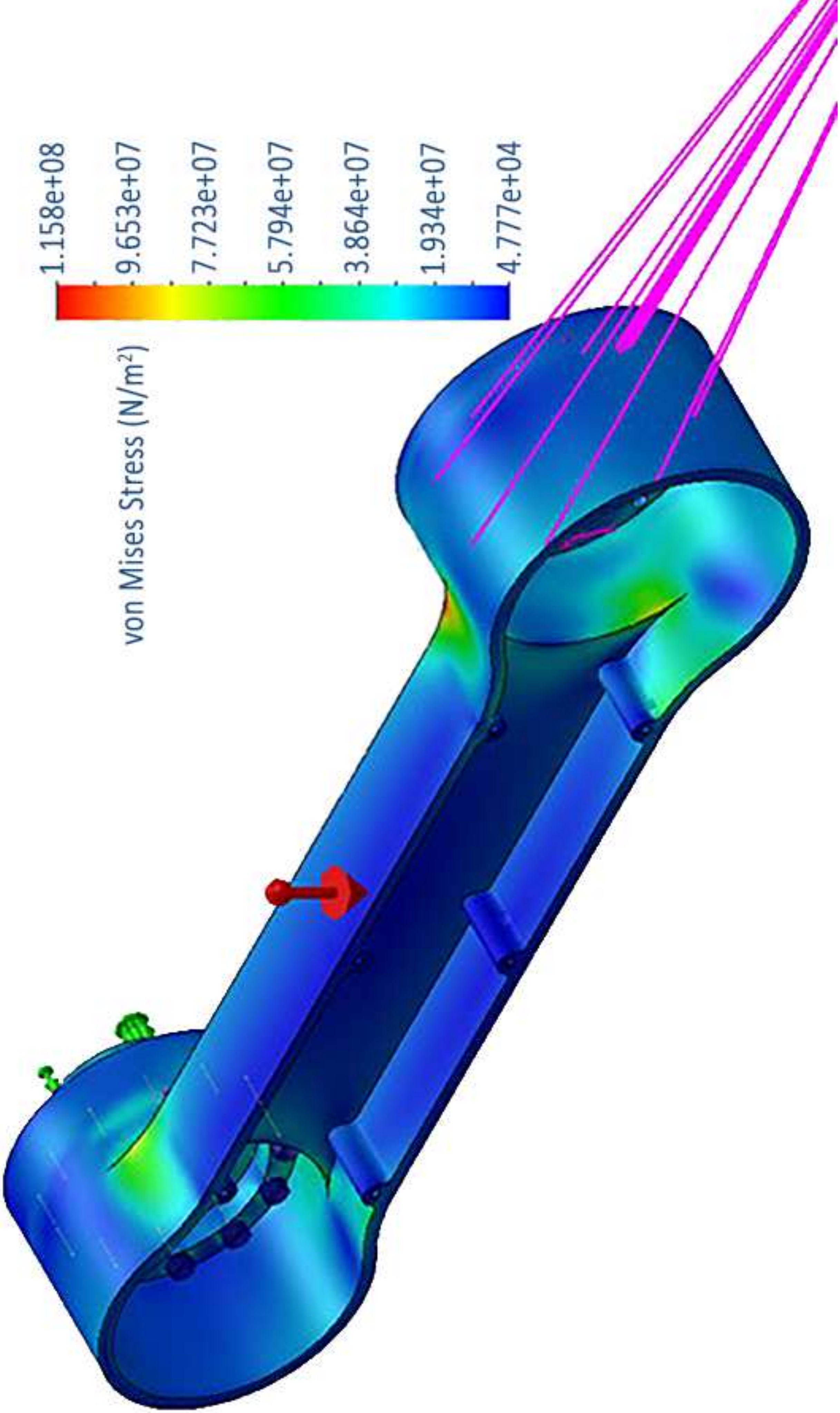

Click here to access/download;Figure;Fig6.eps

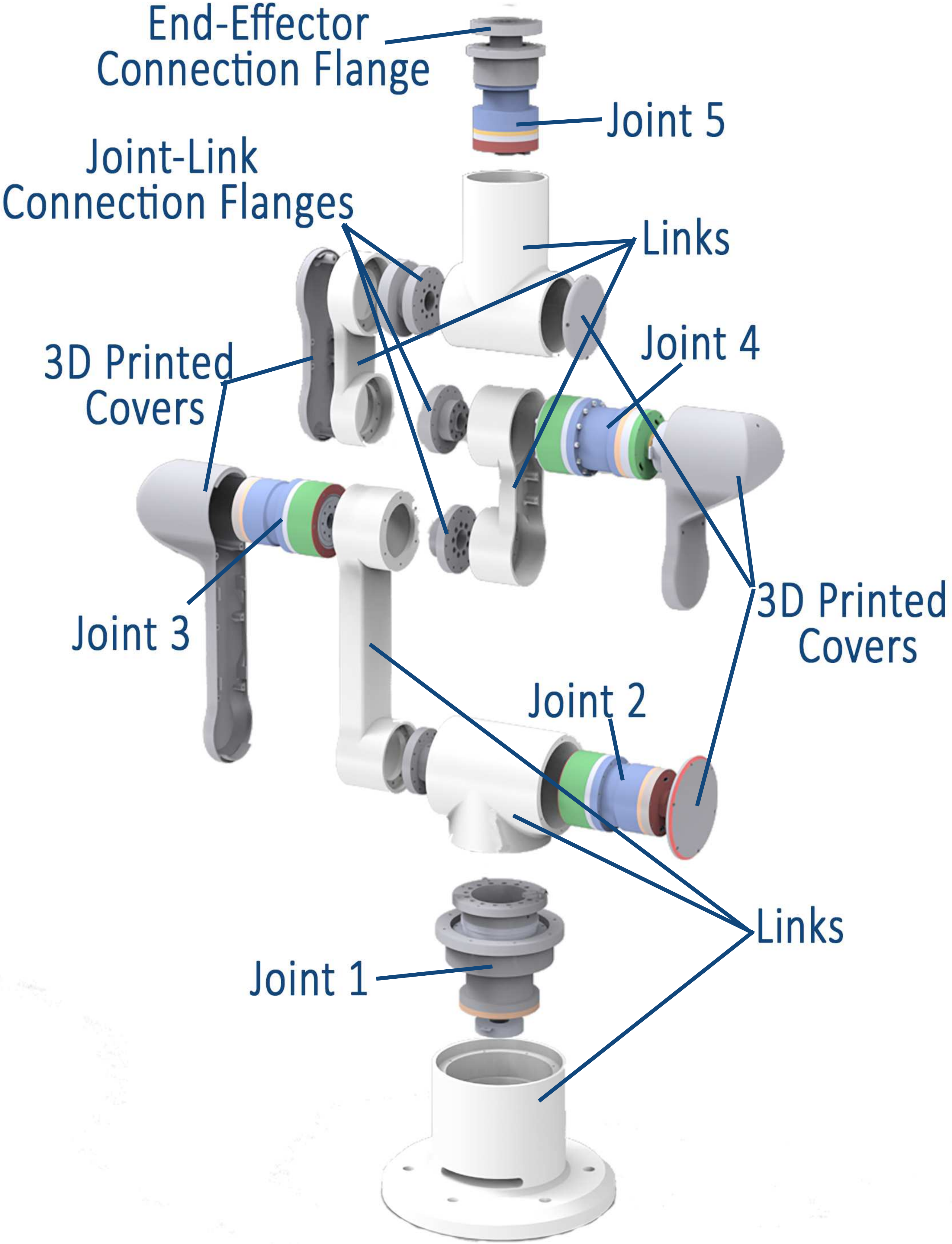

Click here to access/download;Figure;Fig7.eps

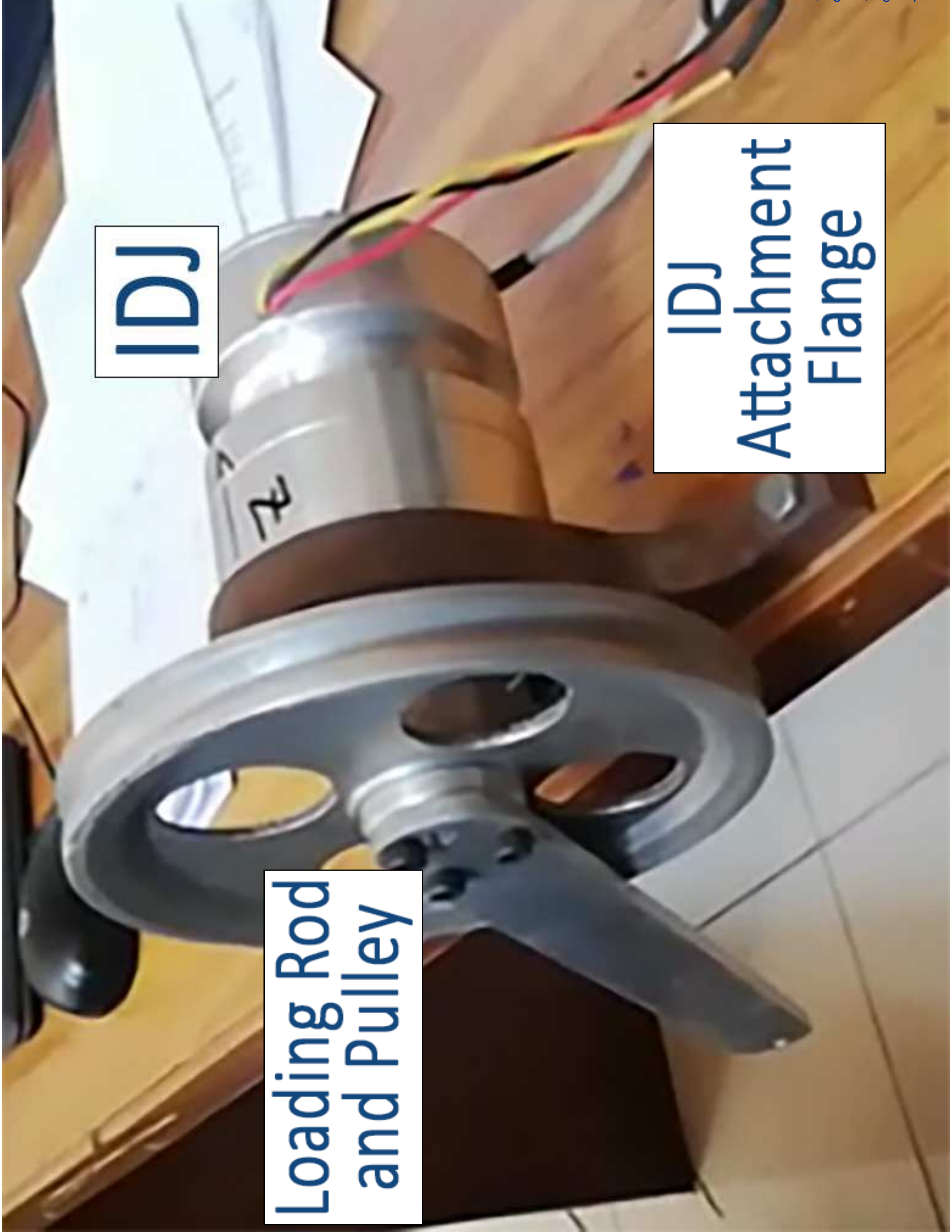

Click here to access/download;Figure;Fig8.eps

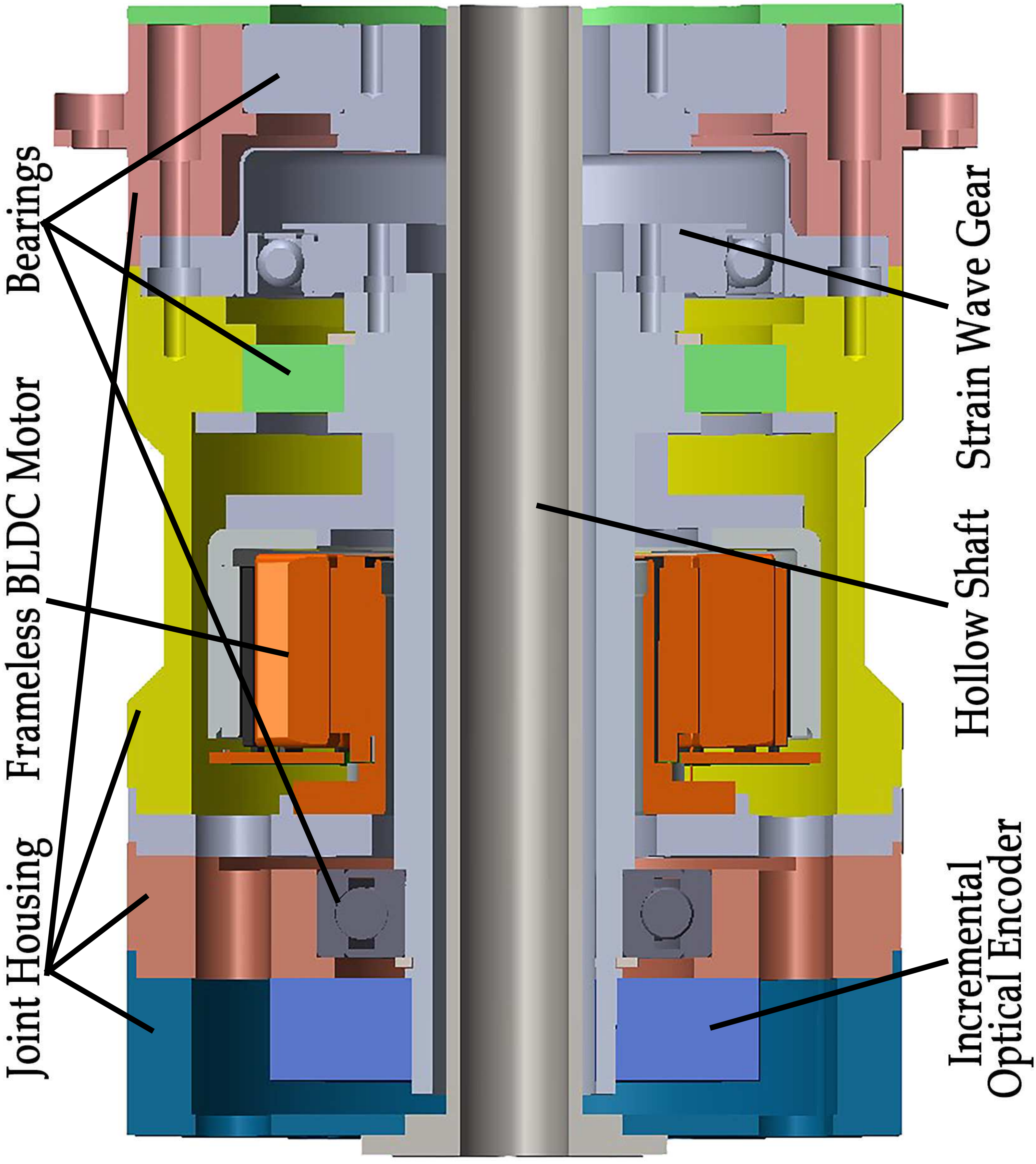

Click here to access/download;Figure;Fig9.eps

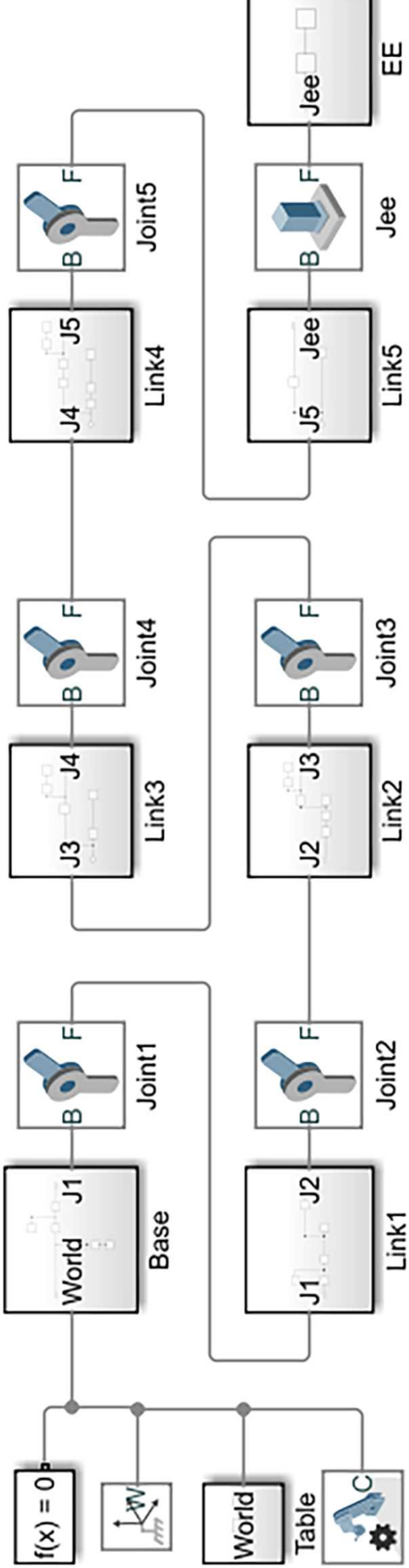

Click here to access/download;Figure;Fig10.eps

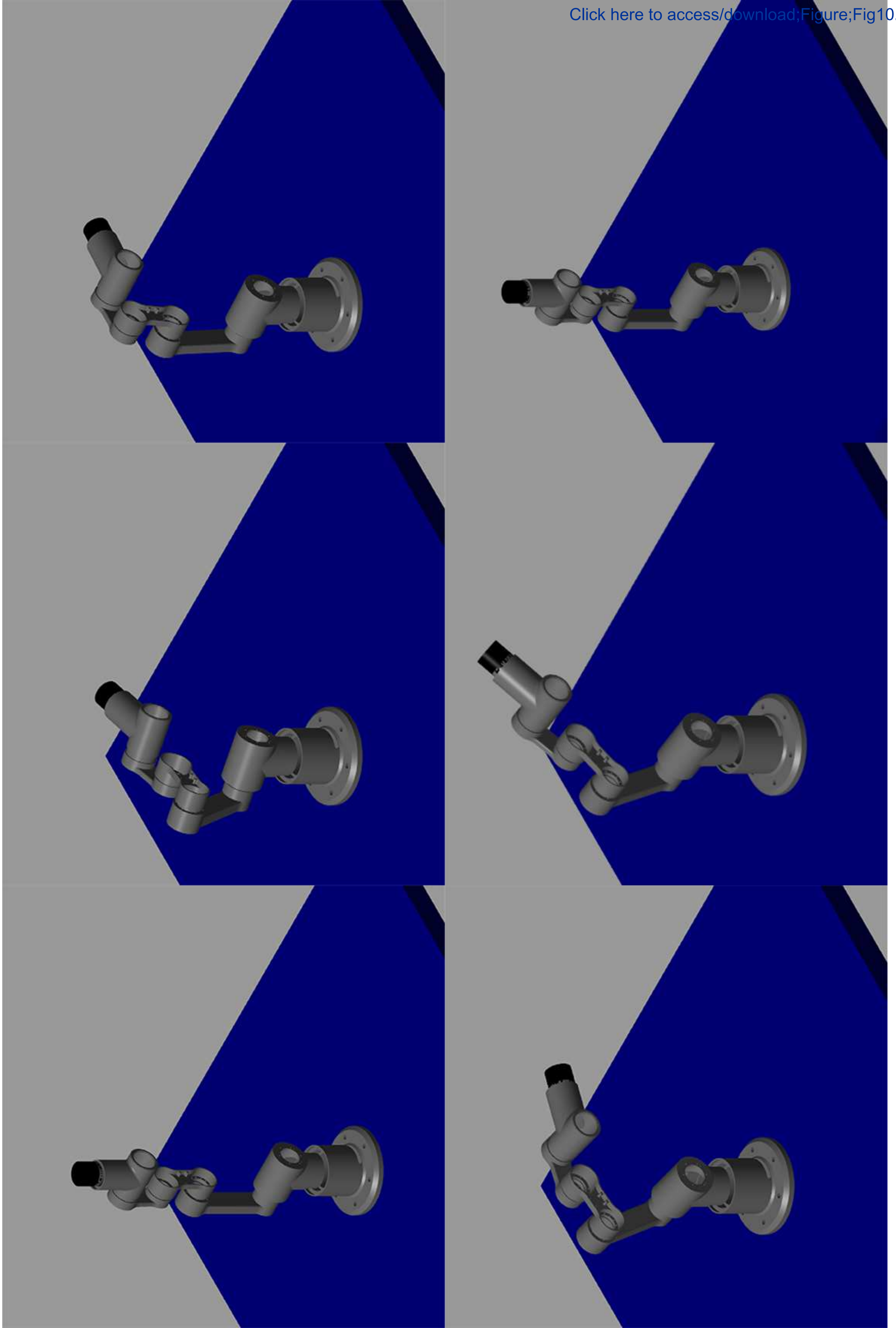

Click here to access/download;Figure;Fig11.eps

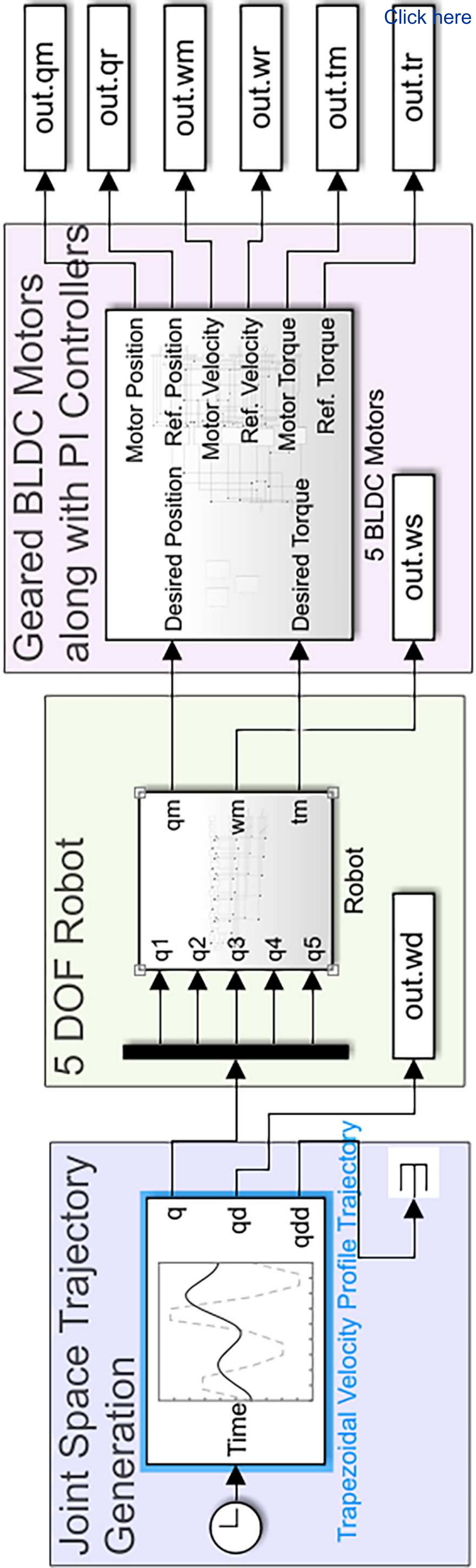

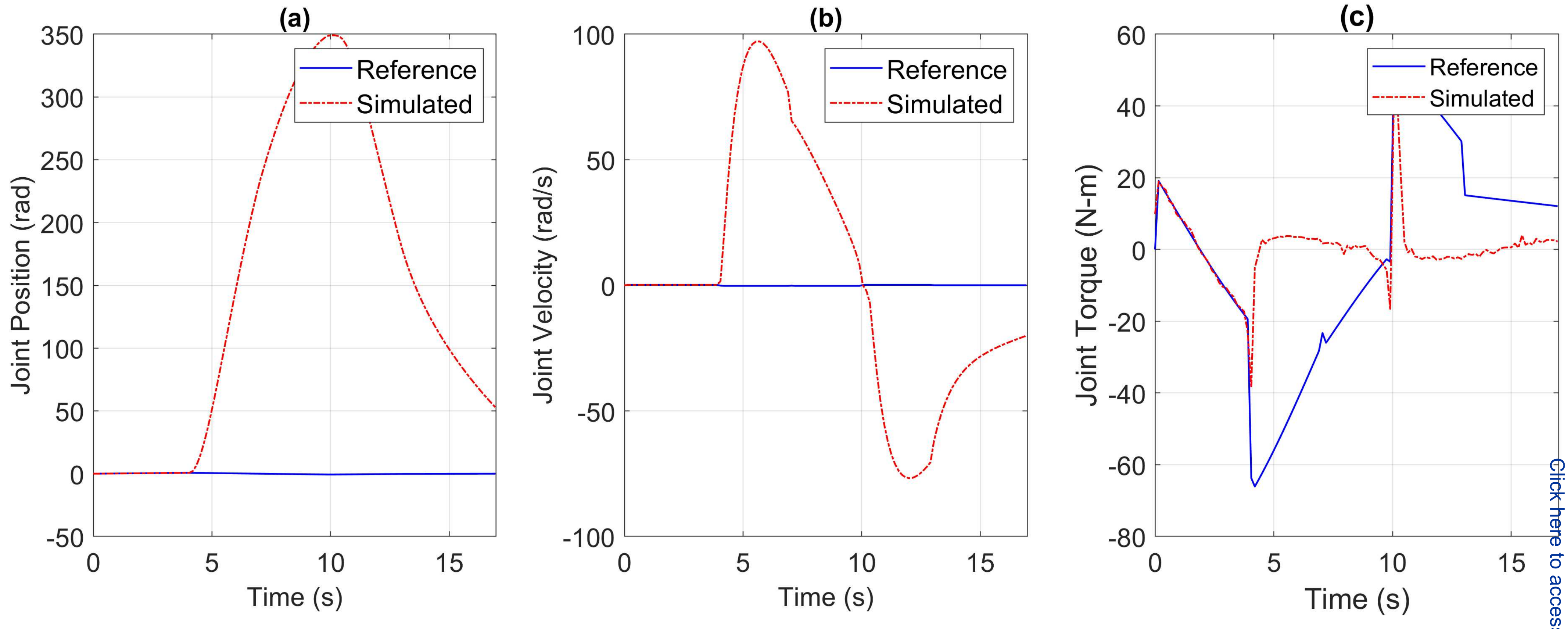

Click here to access/download;Figure;Fig12.eps

Click here to access/download;Figure;Fig13.eps

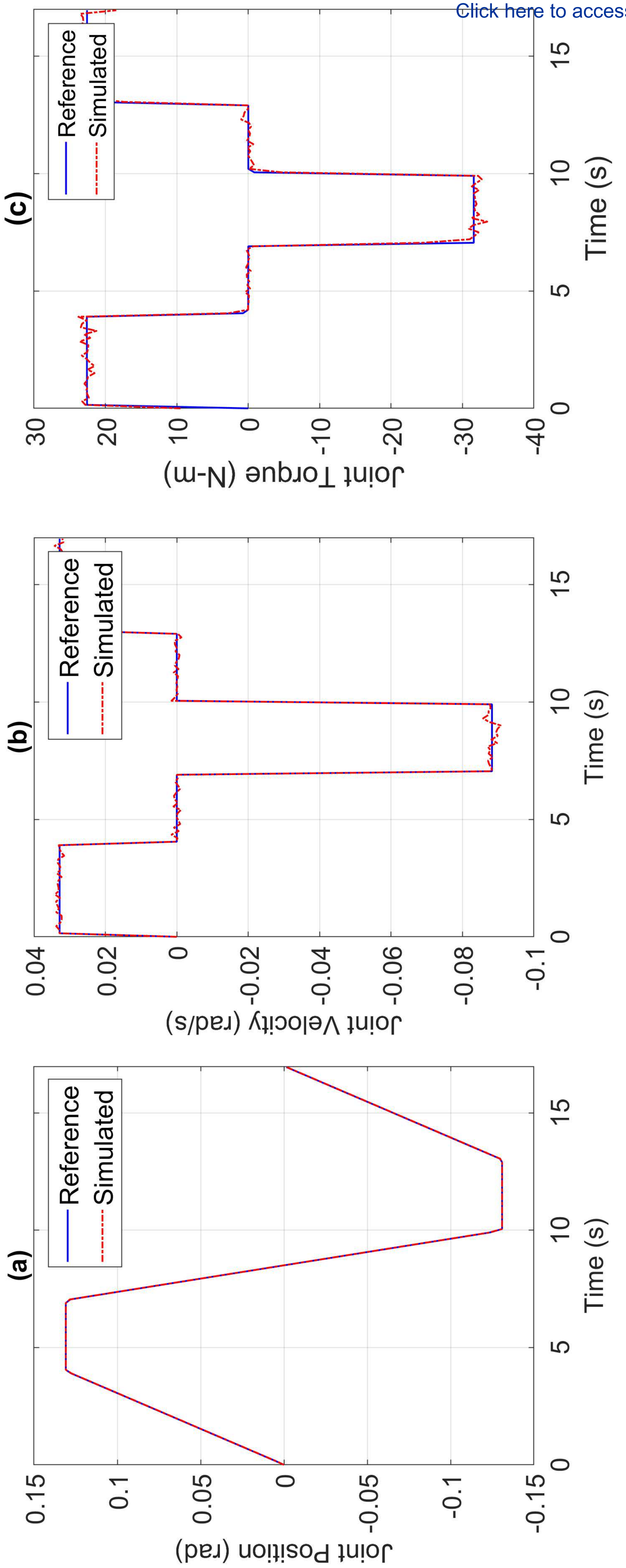

Click here to access/download;Figure;Fig14.eps

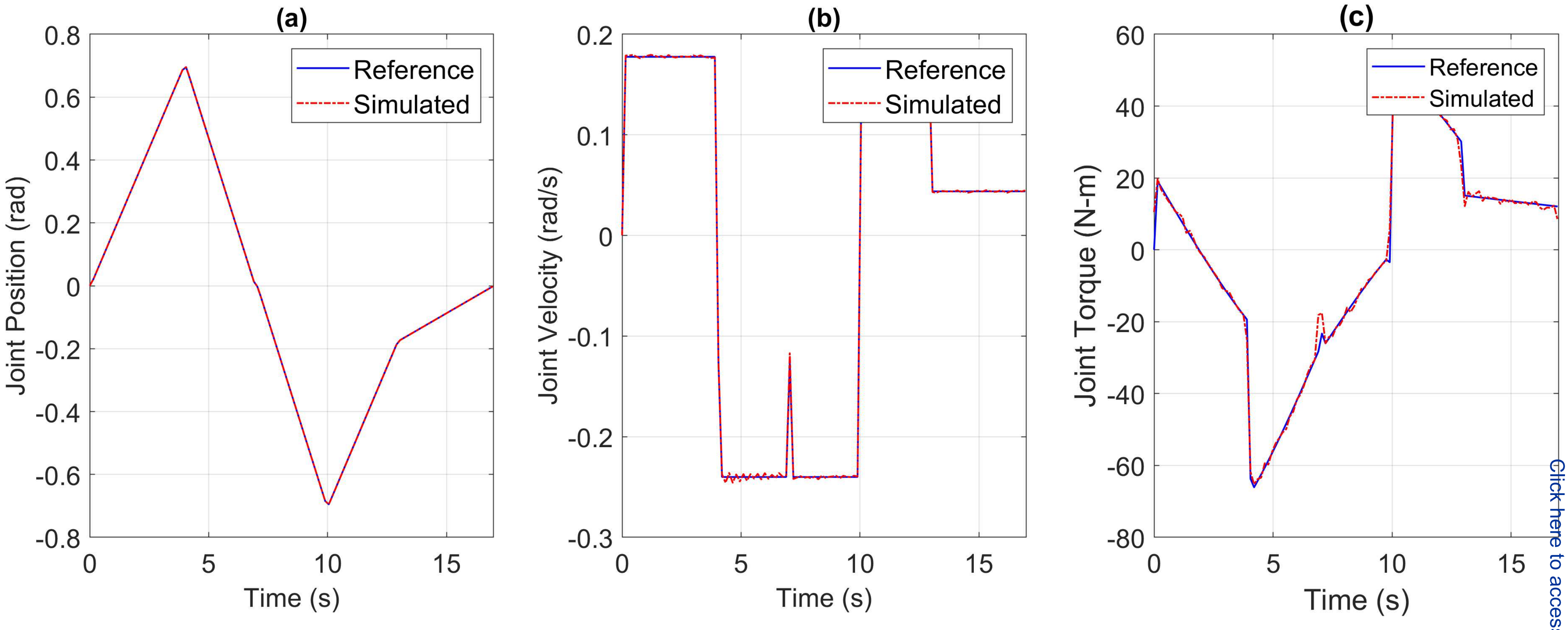

Click here to access/download;Figure;Fig15.eps

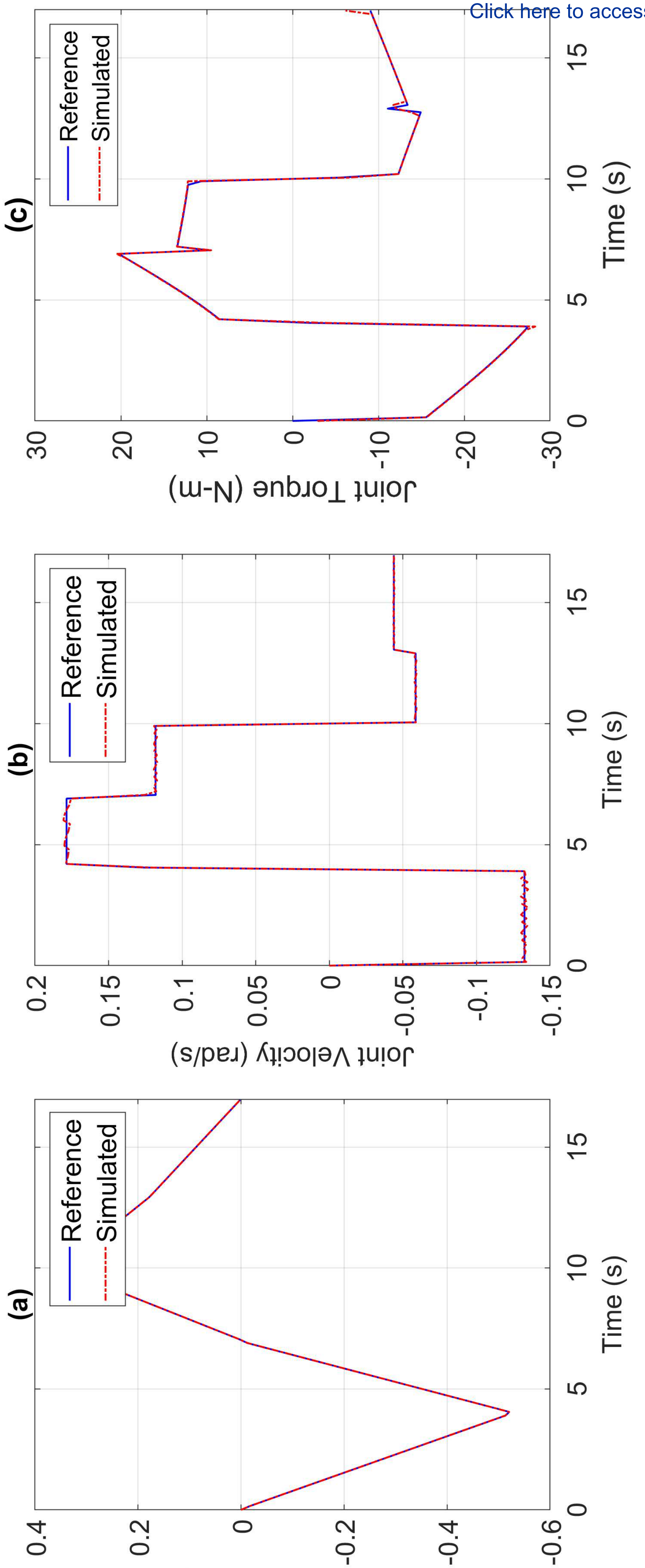

Click here to access/download;Figure;Fig16.eps

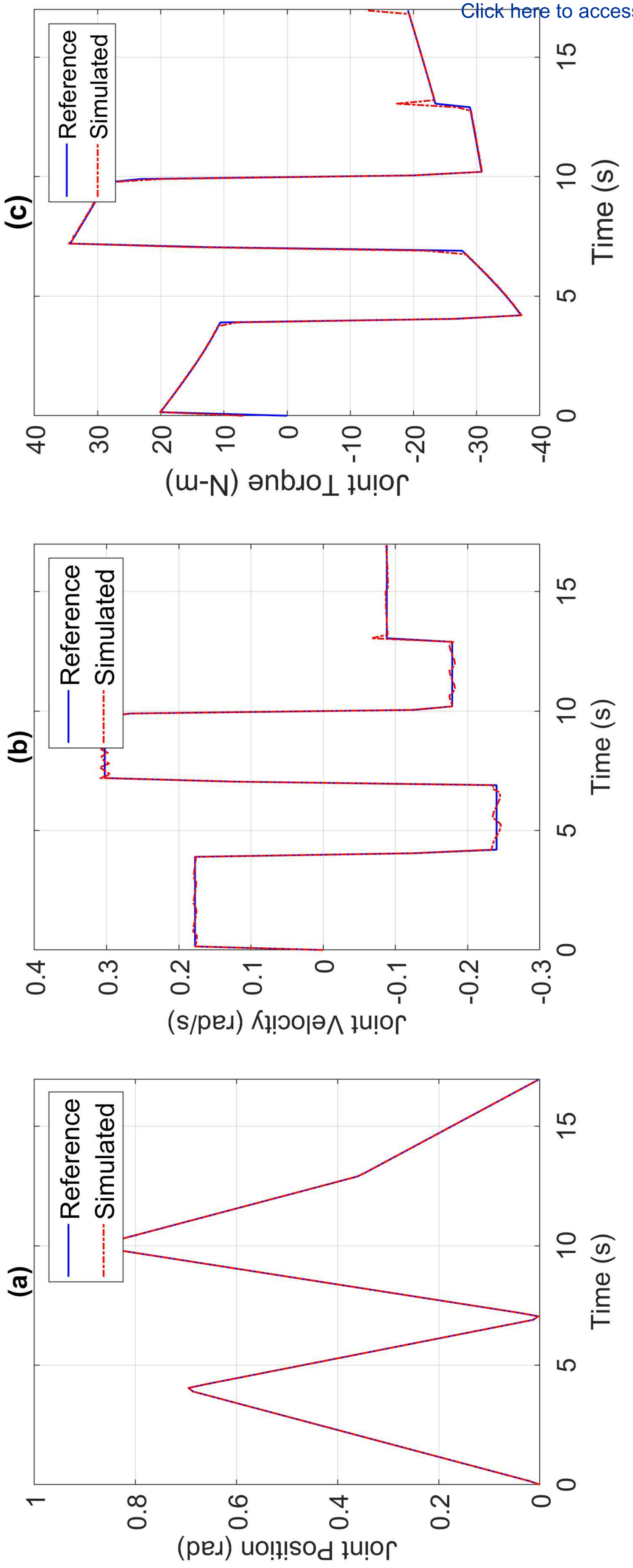

Click here to access/download;Figure;Fig17.eps

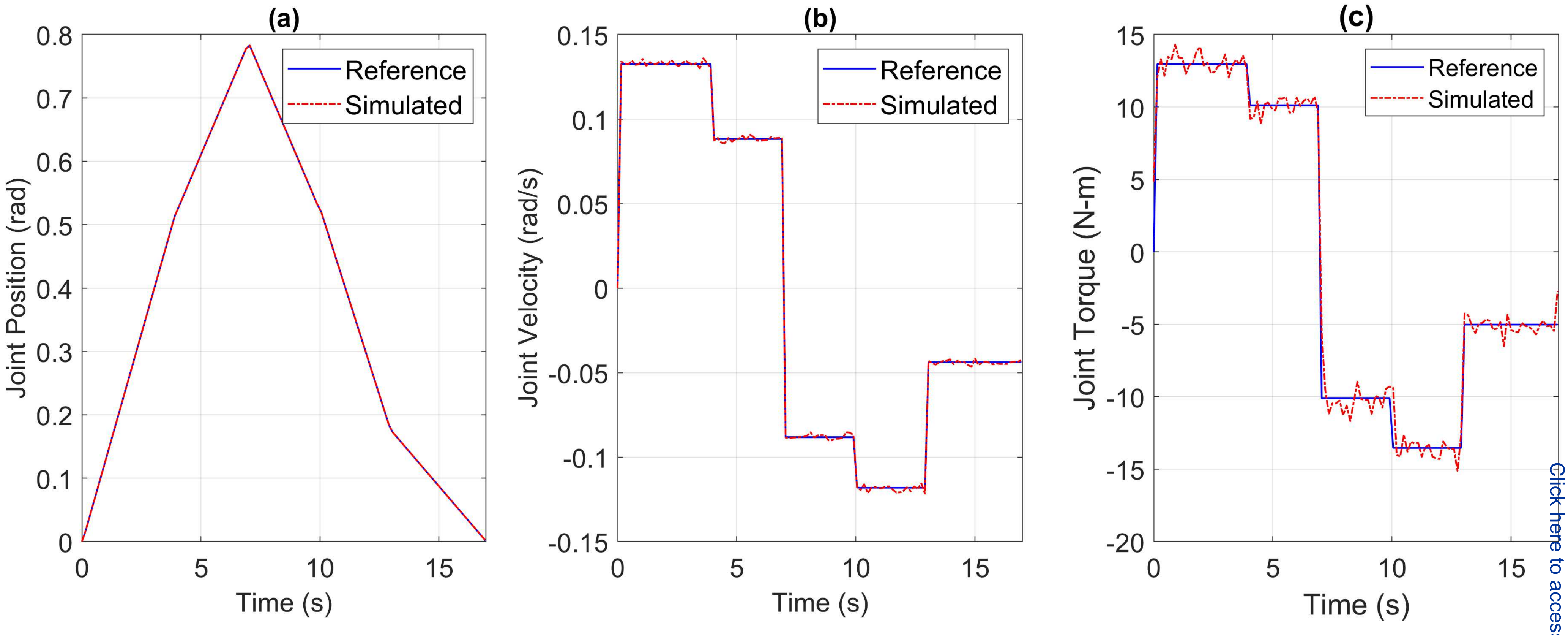

Click here to access/download;Figure;Fig18.eps

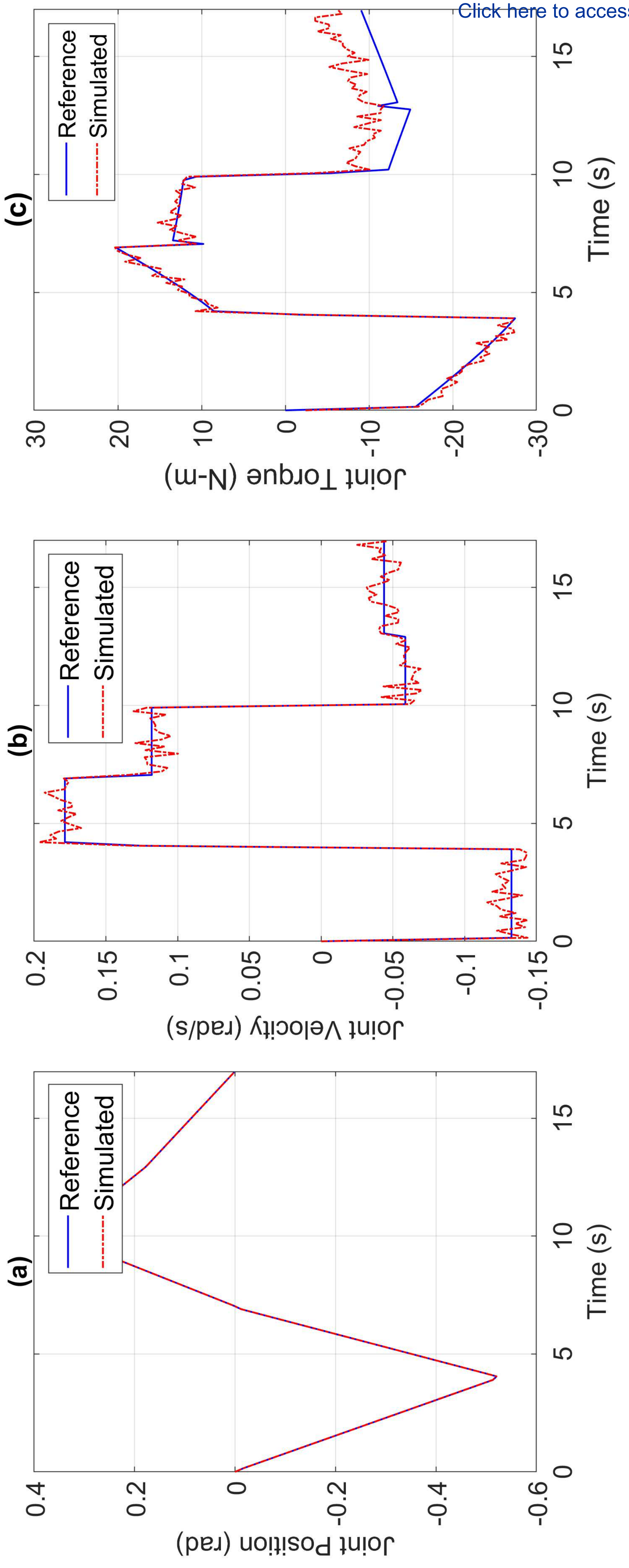

Click here to access/download;Figure;Fig19.eps

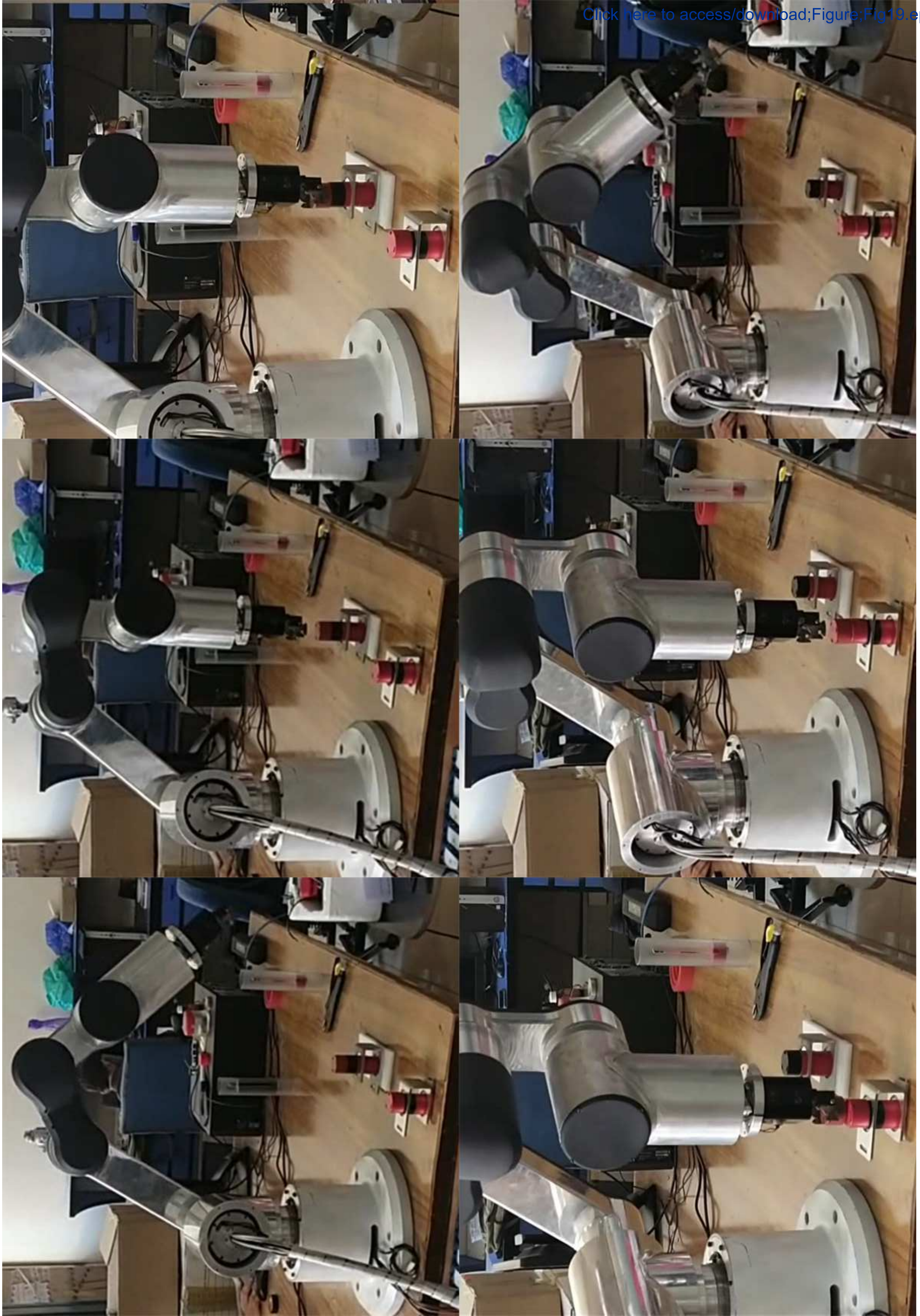

Click here to access/download;Figure;Fig20.eps

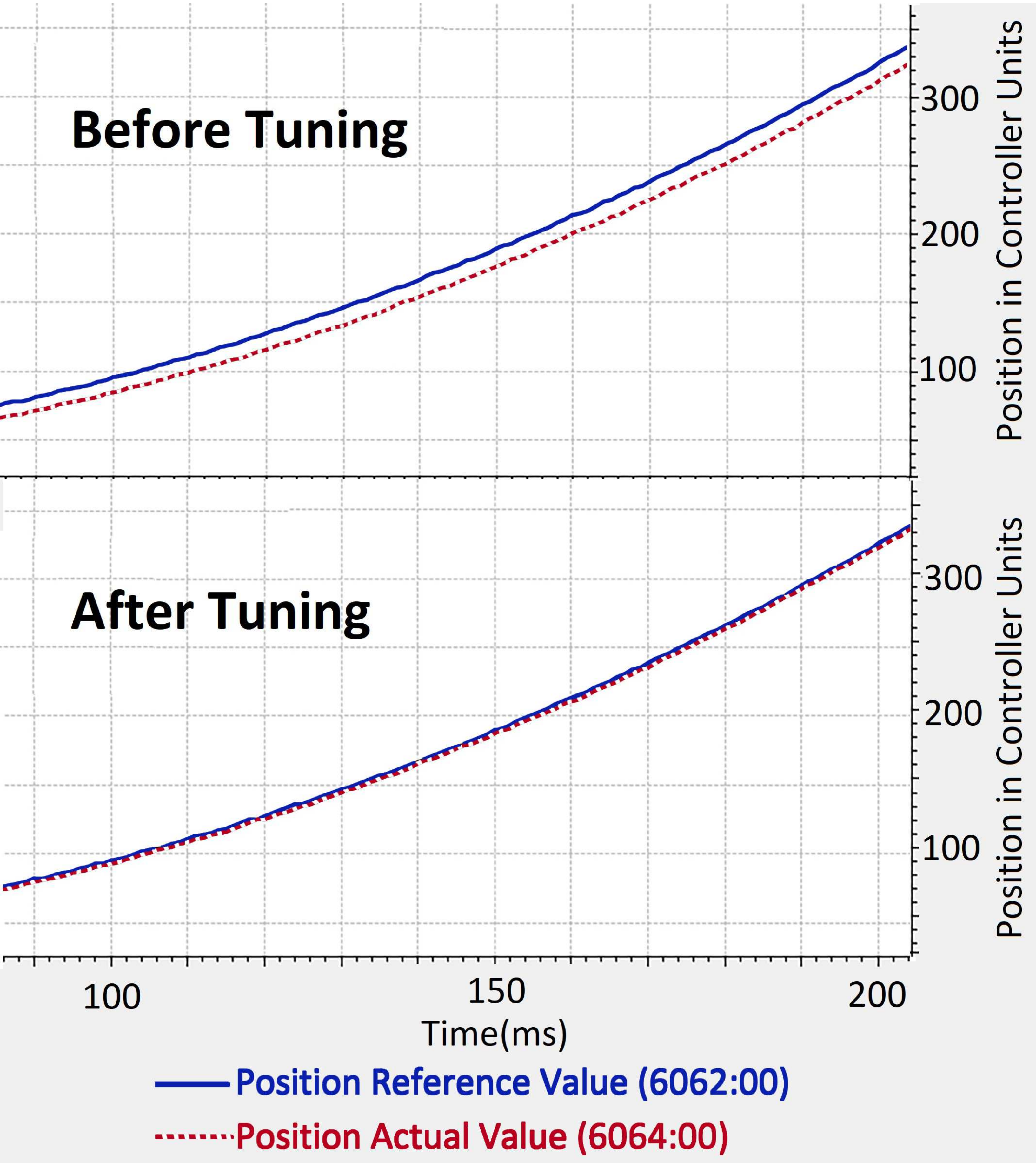

Click here to access/download;Figure;Fig21.eps

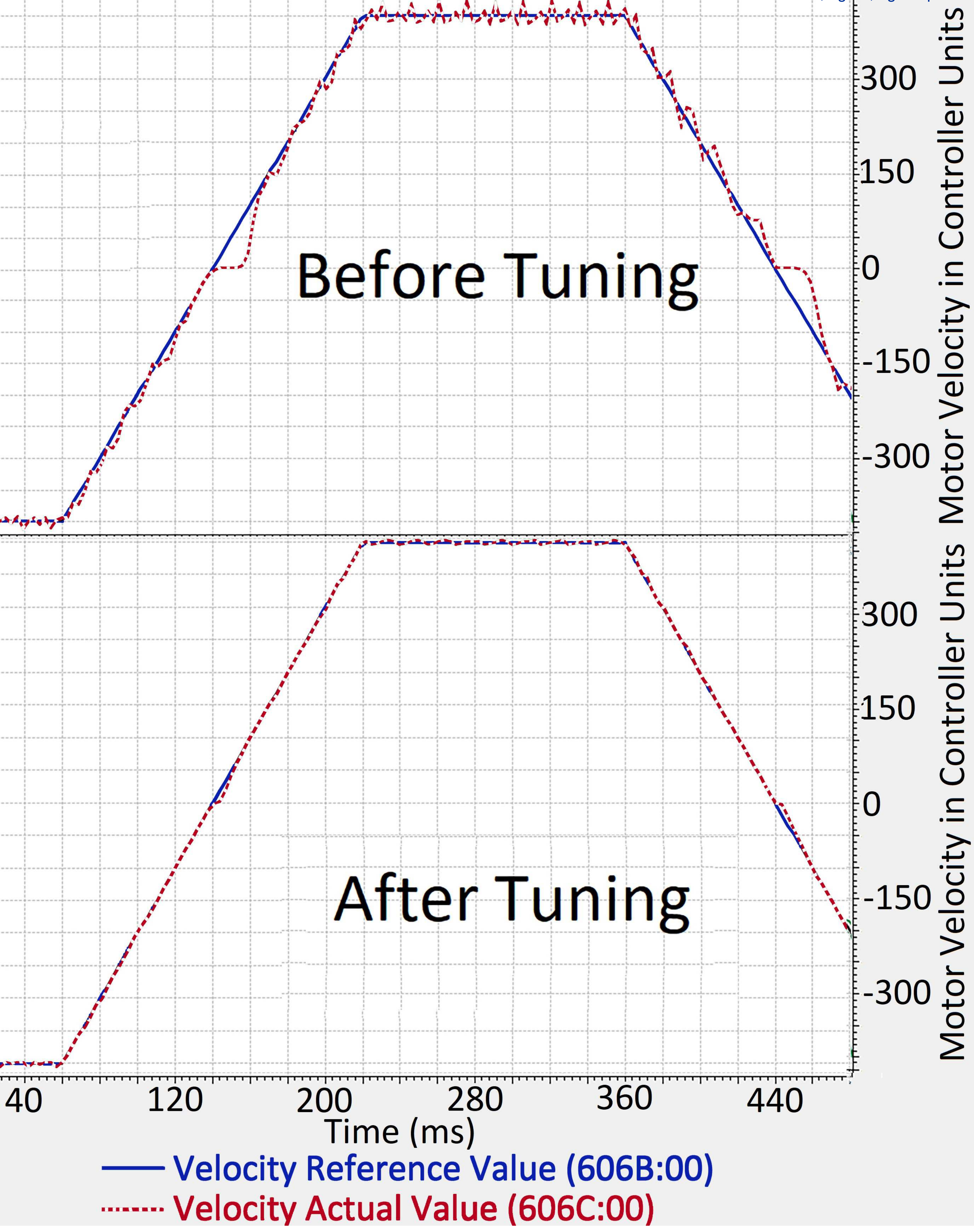

Click here to access/download;Figure;Fig22.eps

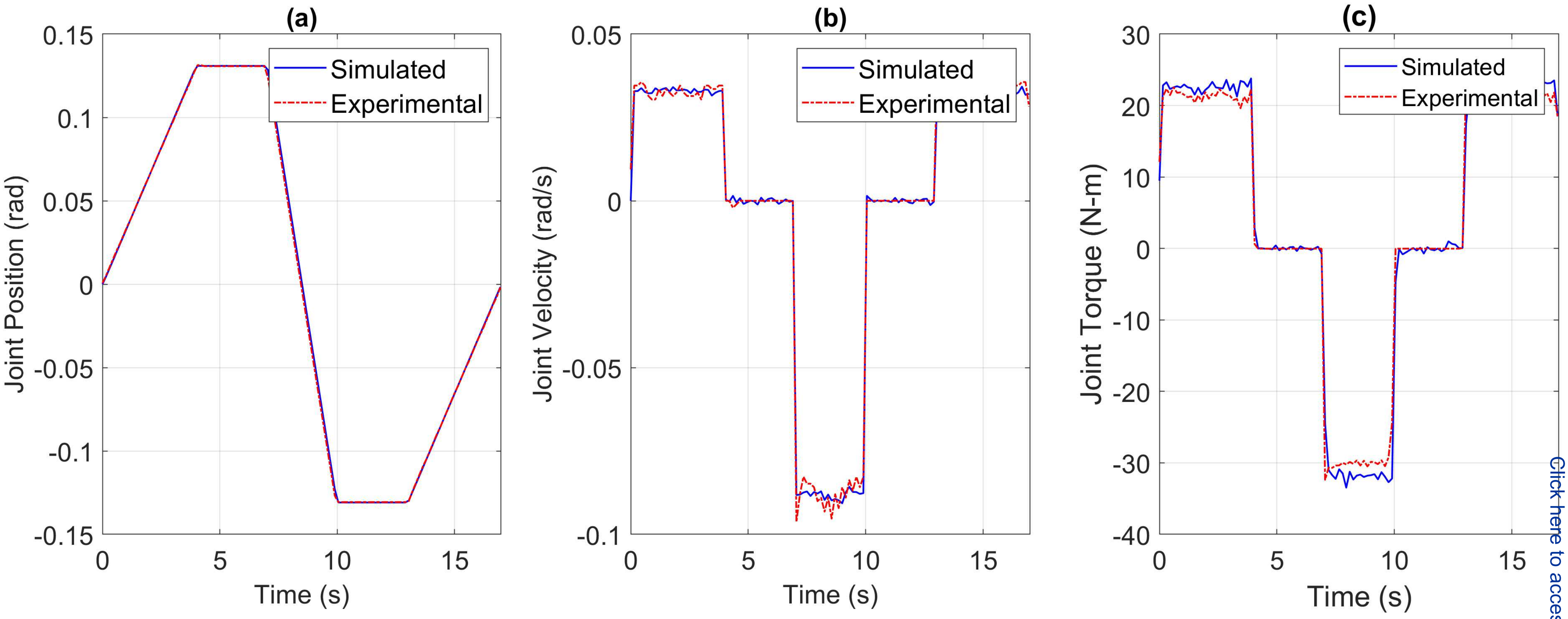

Click here to access/download;Figure;Fig23.eps

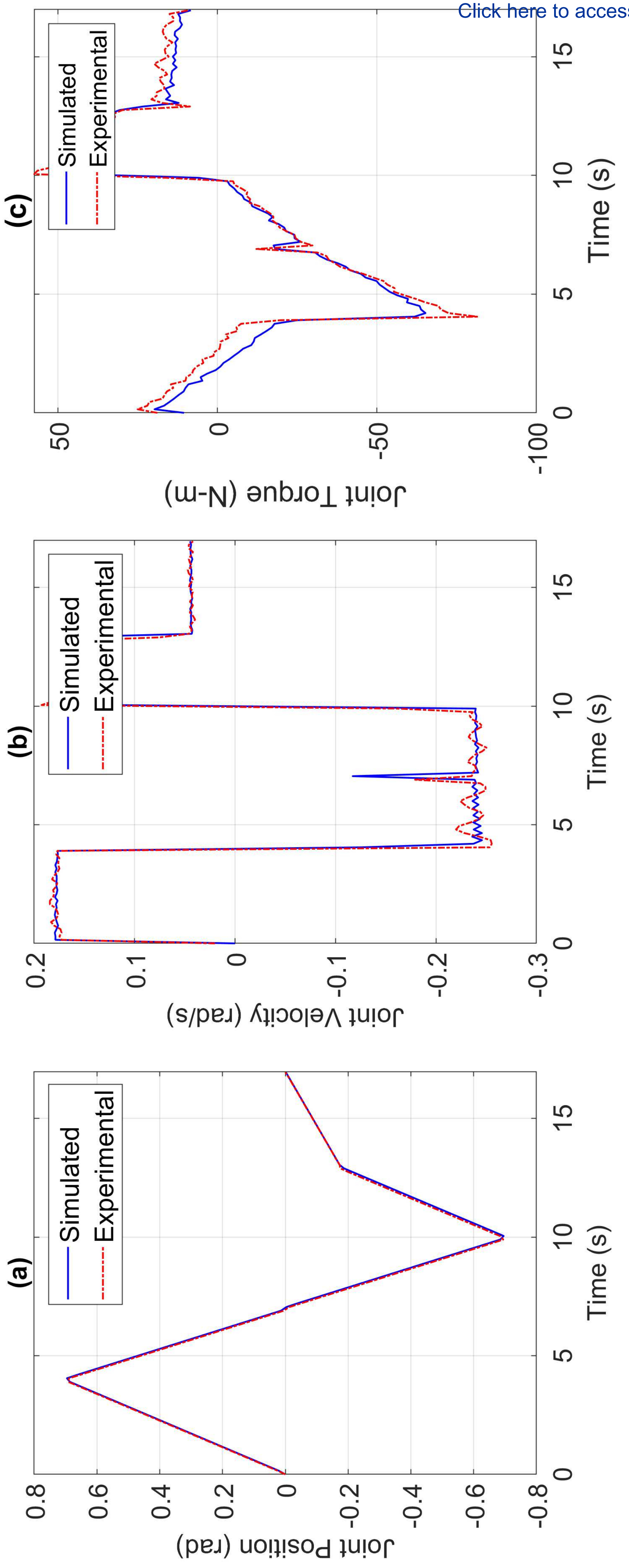

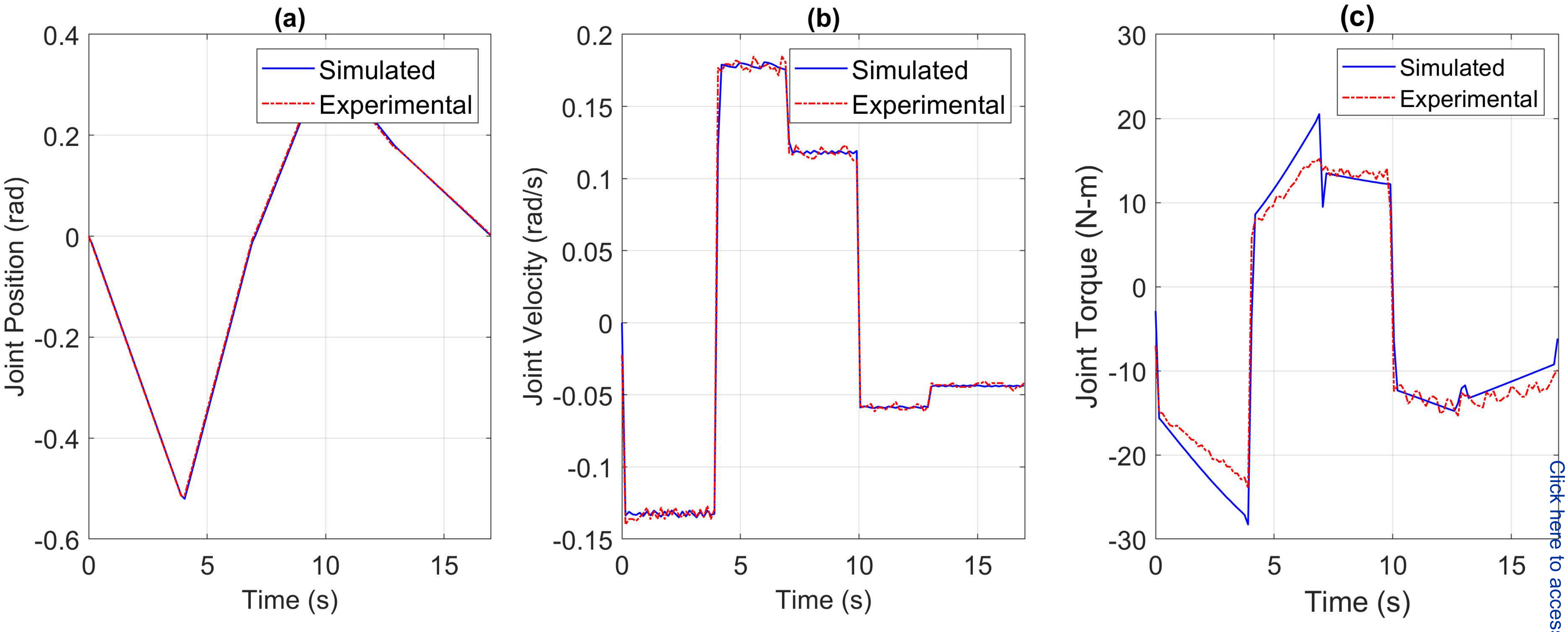

Click here to access/download;Figure;Fig24.eps

Click here to access/download;Figure;Fig25.eps

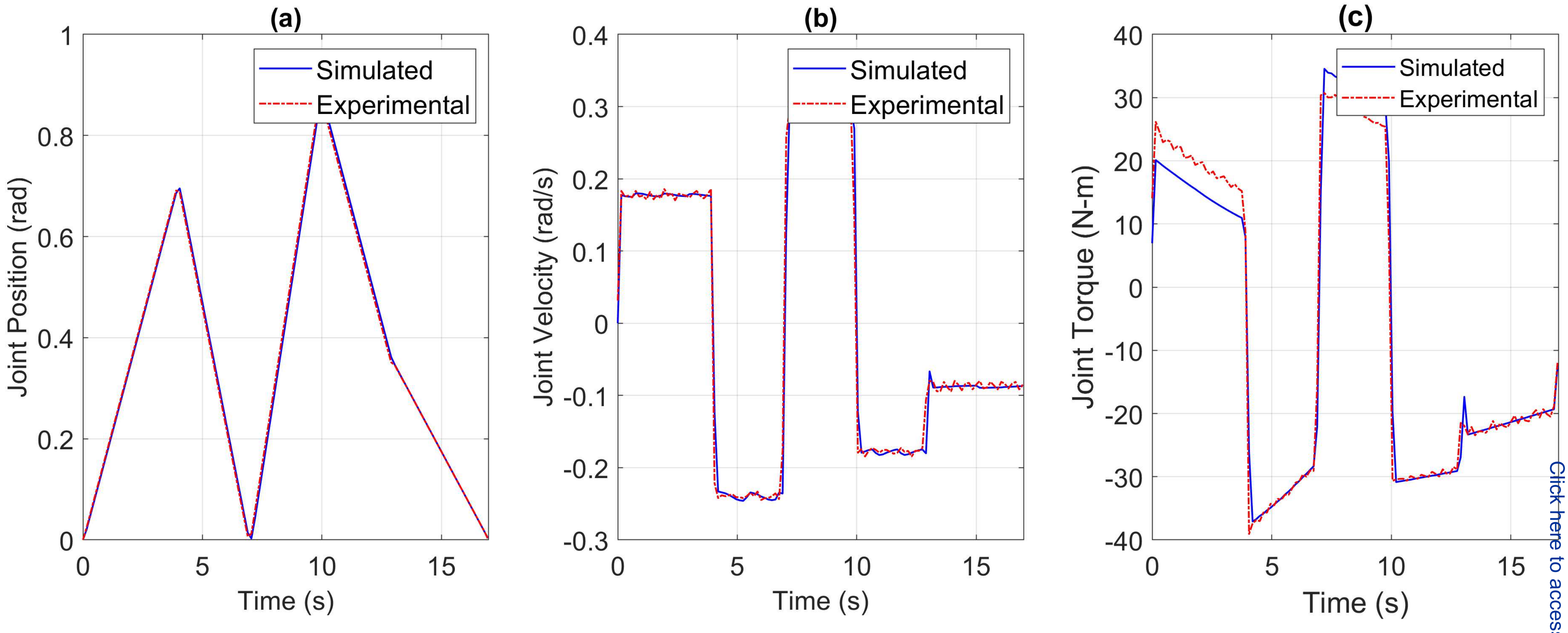

Click here to access/download;Figure;Fig26.eps

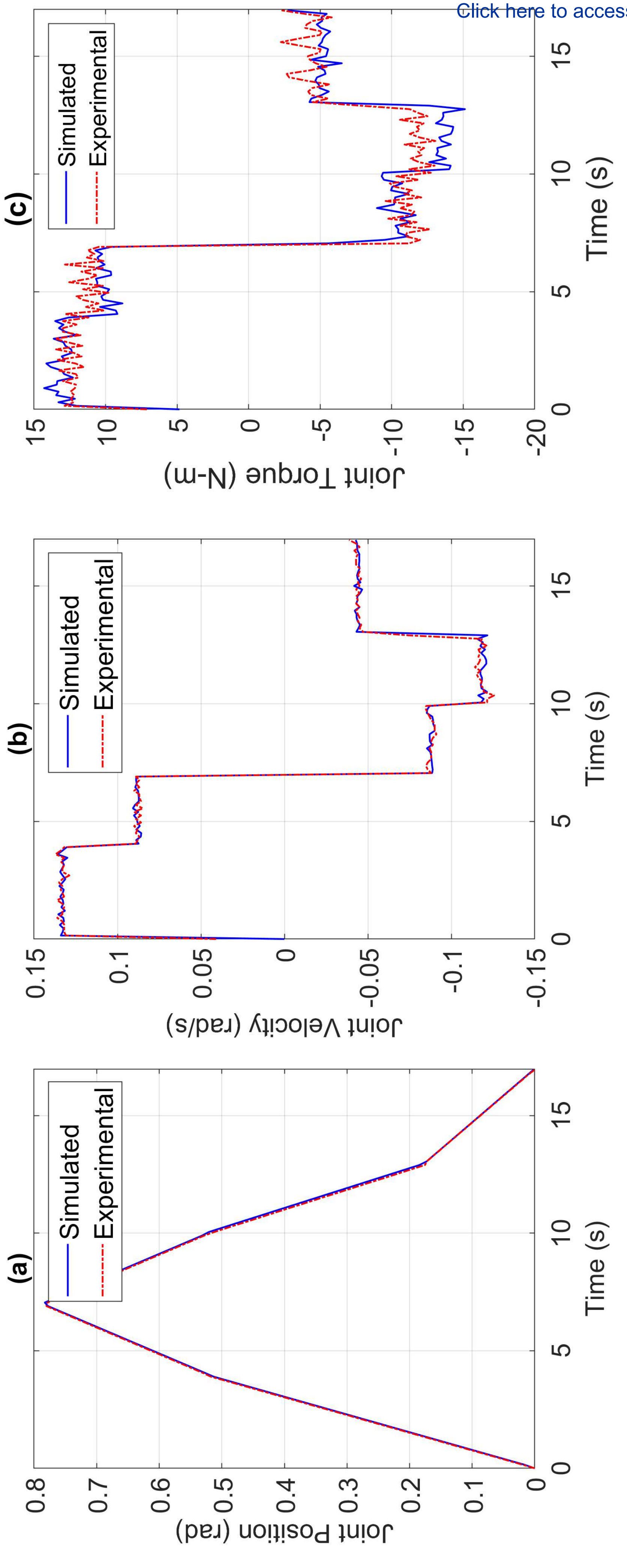

Click here to access/download;Figure;Fig27.eps

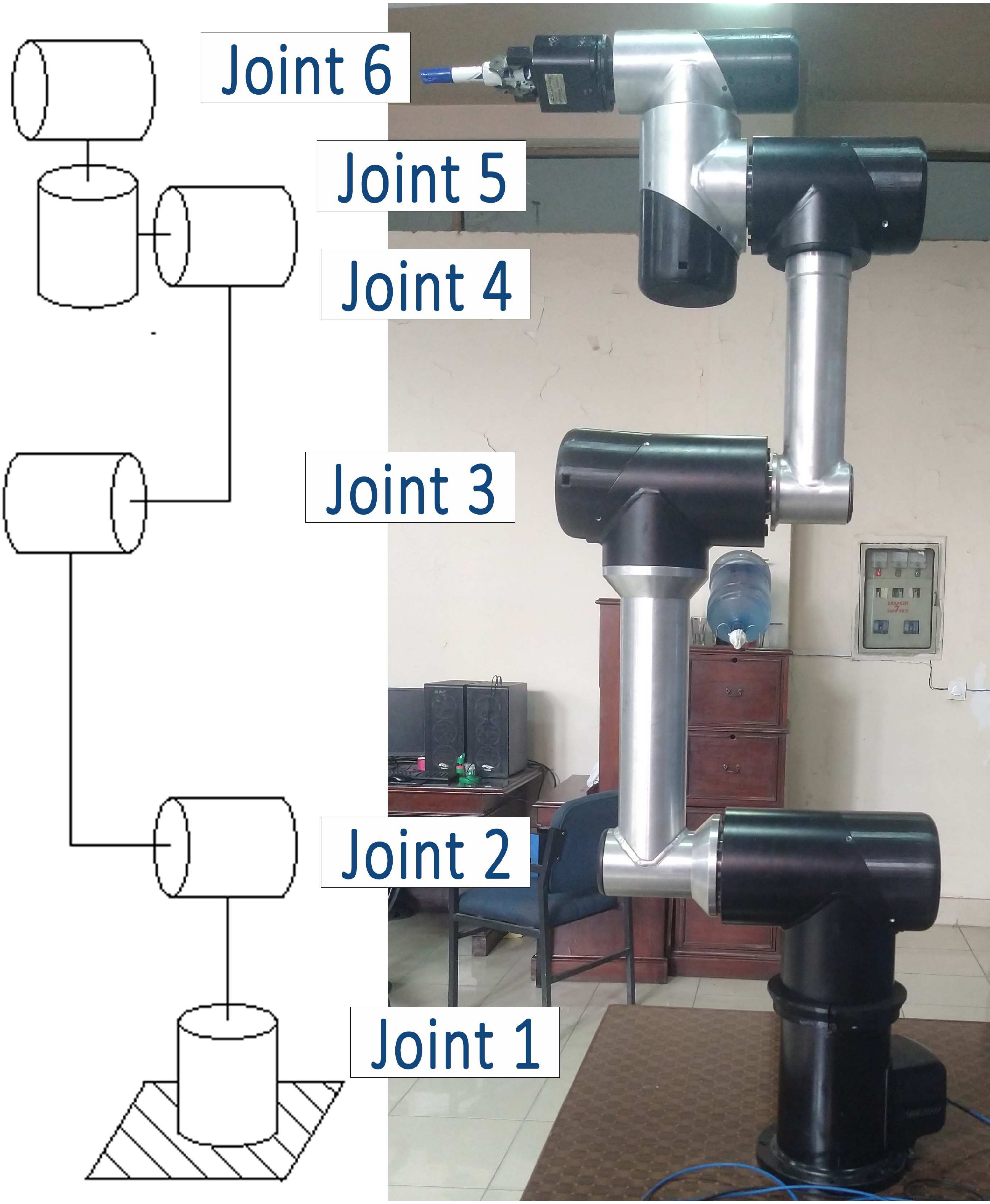

Experimental Development of Lightweight Manipulators with Improved Design Cycle Time that leverages off-the-shelf Robotic Arm Components

Muhammad Rzi Abbas[1], Muhammad Ahsan[1], Jamshed Iqbal[2]
[1]University of Engineering & Technology, Lahore, Pakistan
[2]University of Hull, Hull, United Kingdom

Abstract

The growing market for lightweight robots inspires new use-cases, such as collaborative manipulators for human-centered automation. However, widespread adoption faces obstacles due to high R&D costs and longer design cycles, although rapid advances in mechatronic engineering have effectively narrowed the design space to affordable robot components, turning the development of lightweight robots into a component selection and integration challenge. Recognizing this transformation, we demonstrate a practical framework for designing lightweight industrial manipulators using a case-study of indigenously developed 5 Degrees-of-Freedom (DOF) cobot prototype. Our framework incorporates off-the-shelf sensors, actuators, gears, and links for Design for Manufacturing and Assembly (DFMA), along with complete virtual prototyping. The design cycle time is reduced by approximately 40% at the cost of cobot real-time performance deviating within 2.5% of the target metric. Our physical prototype, having repeatability of 0.05mm calculated as per the procedure defined in ISO 9283:1998, validates the cost-effective nature of the framework for creating lightweight manipulators, benefiting robotic startups, R&D organizations, and educational institutes without access to expensive in-house fabrication setups.

Keywords

Lightweight robot, Cobot, Robot Design Process

Section I

Introduction

The field of robotics has undergone a significant evolution, particularly with the emergence of lightweight robots, aligning seamlessly with the requisites of Industry 5.0 (1). This progression has paved the way for substantial advancements in collaborative robotics, where lightweight robots, often referred to as "cobots," are tailored for harmonious human-robot interaction in applications ranging from industrial collaboration to medical-related scenarios (2-6). These versatile entities boast characteristics that position them as adaptable collaborators

within cooperative work environments. Their intrinsic attributes, characterized by reduced speeds and inertias, make them particularly conducive for seamless interaction with human counterparts, leading to a broadening spectrum of applications across diverse domains. From assembly line installations (7) to medical setups (8), from education sector (9) to space endeavors (10) , these robots have found their utility across varied industries. The escalating demand for automation positions lightweight robots as pivotal players across industrial sectors (1). In this evolving landscape, our research takes on heightened significance, strategically addressing the imperative for streamlined design and development of lightweight robots, a cornerstone in facilitating the transition toward Industry 5.0-aligned industrial and R&D ecosystems.

Conventional engineering product design cycle (11) is illustrated in Fig 1 which is used to argue about robotic product development cycle. The whole process may be divided into seven major stages as labeled by circled digits in the figure.

Fig 1: Typical Robot Design and Development Cycle. The table in the figure shows typically what percentage of total design cycle time (12-14) is spent on each stage. The portions enclosed in Red Boxes are either eliminated or simplified through the proposed scheme.

- Stage 1 is the initial stage where the concept of the robot is formulated, and its basic design is outlined. It involves defining the robot's purpose, capabilities, and basic architecture. Typically, 10%[1] (12-14) of the total design cycle time is spent in this stage.
- Stage 2 relates to the detailed design of the robot's components, such as mechanical, electrical, and software aspects. Planning for the manufacturing and assembly processes also occurs in this stage, and it takes around 20% of the total design cycle time.
- Stage 3 implements the initial design, usually in simulated environments, which is a quicker method, or sometimes a physical prototype is also developed, that normally takes longer. Physical prototype development consumes around 15% of the design cycle time.
- Stage 4 tests and iterates the prototype based on several performance parameters. Typically, 15% of the design cycle time is spent on this activity.
- Stage 5 starts once the design is optimized and tested. The final version of the robot design is prepared for manufacturing. This includes sourcing components, manufacturing, and assembly. Normally 20% of the total design cycle time is utilized in this stage.
- Stage 6 tests and validates the manufactured robot to make it deployment ready. This stage typically consumes 15% of the total design cycle time.
- Stage 7 is related to production of documentation, user manuals, training material and deployment of the robot in the intended environment. This stage lasts around 5% of the total design cycle time.

[1] These values are based on general industry knowledge and practices. The breakdown of time spent on different stages of robot development can vary widely depending on the specific project, organization, and goals. These percentages are meant to give a rough idea of how time may classically be allocated in a typical robot development process.

Considering the extensive range of commercially available components, it would be both economical and timesaving for developers to simulate robotic manipulators exclusively composed of such components. The design approach presented in this paper modifies the robot development process along these lines, hence reducing the complexity and required time. First, stage 1 is reduced to only gathering of the robot specifications. Currently, these specifications are the degrees-of-freedom, payload capability, reach, and maximum workspace speed of the end-effector. The time needed to develop initial conceptual design based on the given specifications ~~has been~~will be reduced because of the developed library of components. Once the library of subcomponents has been established, our proposed scheme will only use validated models of commercially available components to design the robotic manipulator. Hence stage 1 can almost be eliminated resulting in saving of around 10% of the total design cycle time. Second, the modeling of the subcomponents allows for virtual prototyping, eliminating the need for physical prototype development, saving ~~around~~ additional ~~20~~10% of the total design cycle time by simplifying stage 2. Moreover, the developed library eliminates the requirement of subcomponents' performance analysis, further saving valuable development time. Lastly, the testing and validation of the manufactured robot is ~~eliminated~~ greatly reduced because the virtual prototype is made of all commercially available or produced components. The Pearson's Correlation Coefficient and Root Mean Squared Error (RMSE) of trajectory tracking~~,~~ presented in the experimentation section of this paper, between the performance of the virtual prototype and the developed robot confirms this claim. Lastly, for the functional performance validation, our criterion requires that the physical prototype attains repeatability of the order of magnitude same as that of the commercially available lightweight robot. Table 7 in Section IV Hardware Experimentation validates that our developed prototype exhibits the desired performance.

Formatted: Font: 12 pt

Formatted: Font: 12 pt, Font color: Auto

Formatted: Font: 12 pt, Font color: Auto

This research work focuses on the simulation of hollow shaft Integrated Drive Joints (IDJs) integrated into lightweight robotic manipulators designed to operate as cobots. The developed library encompasses models of several commercially available subcomponents. Within the simulation environment, the IDJ models and the manipulator's structure are utilized to identify the ~~optimal~~ suitable components, the frameless BLDC Motor and the strain wave gear, for the integrated drive joints. Moreover, the simulation of the complete structure enables the consideration of coupled dynamics throughout the entire system, which is significant to control system engineers. While a single IDJ exhibits distinct performance characteristics, coupling multiple joints introduces coupled dynamic effects across all joints (15, 16). These couplings can be mitigated at the structural level through the appropriate selection of gearing systems or addressed at the software level by fine-tuning the control system accordingly.

The simulation tool was developed using the MATLAB/Simulink R2021b environment, while the robot structure was modeled using SolidWorks 2021. Subsequently, based on the findings obtained from the simulation tool, a 5 degrees-of-freedom (DOF) lightweight robot was developed, as depicted in Fig2. Though the authors have also developed a 6-DOF lightweight robot by following the same footsteps, but for the sake of compactness of the article, the detailed analysis of 5-DOF robot is presented.

Fig 2: 5 DOF Robot's FBD (Left) and 5 DOF robot's hardware (Right).

Table 1: Man-hours saved in result of following the proposed scheme for designing and developing a 5 Degrees of freedom Lightweight Robotic Manipulator in a typical R&D Lab

**Stage**	**Title**	**Conventional Design Approach's Initial R&D man-hour estimate**	**Actual R&D man-hours under the proposed scheme**	**Man-hours saved**	**Percentage of the Design Cycle Time saved**
1	Conceptual Design	160	32	128	5.9
2	Detailed Design	320	320	0	0.0
3	Assembly Design Model	480	480	0	0.0
4	Design Analysis	320	64	256	11.9
5	Fabrication	400	400	0	0.0
6	Hardware Iterations	480	0	480	22.2
7	Commissioning	80	80	0	0.0
**Total Percentage of saved Design Cycle Time**					**38.6**

Formatted: Font: Bold

Formatted: Font: Bold

Table 1 summarizes the potential time savings offered by the proposed method for developing robotic manipulators. This data, based on research conducted at the Human Centered Robotics Lab, demonstrates a 38.6% reduction in the total design cycle time. The table itself is divided into six columns. The first two columns list the distinct stages involved in a typical engineering design process for a product. The third column displays estimated man-hours required to complete each stage under the conventional design approach, described in (11).

Formatted: Indent: First line: 0.5"

Equation 1, shown below, details the calculation used to determine these man-hour values.

$$Man\ hours = P\ x\ D\ x\ W \qquad (1)$$

Where:

- P = Number of personnel assigned to a stage
- D = Number of days required to complete a stage
- W = Working hours per day

Column 4 details the actual man-hours consumed during the design and development of a 5-DOF lightweight robotic arm. Column 5 quantifies the man-hours saved by implementing the proposed approach, while Column 6 expresses these savings as a percentage. The sum of these percentages demonstrates the overall 38.6% reduction in design cycle time. It is important to

Formatted: Indent: First line: 0"

note that the time distribution across different design and development stages may deviate from the general percentage values presented in Fig 1.

The organization of the remaining manuscript is as follows: Section II describes the challenges involved in the in-house development of light-weight robots and then propose a suitable methodology for generating an IDJ model that includes a synergetic combination of frameless brushless DC (BLDC) motor, strain wave gear and other subcomponents. Section III showcases the simulation results of the developed model, whereas Section IV details the performance results of the physically developed 5 DOF manipulator based on the in-horse manufactured integrated drive joint using the same hardware subcomponents as those used in the simulation. Lastly, Section V concludes the manuscript and steers the reader to possible avenues of future work.

Section II

Addressing challenges involved in the in-house development of Light-Weight Robots

~~In the current landscape of robot design methodologies, an extensive exploration of both bottom-up and top-down approaches is available in the literature. However, a notable gap persists as many of these analyses overlook the integration of commercially available, off-the-shelf components into the design process. While bottom-up strategies are apt for the development of generic multi-purpose robots, top-down approaches excel in crafting application-specific robotic solutions~~ Robot design methodologies encompass both bottom-up and top-down approaches, which have been extensively explored in ~~the literature~~literature. However, a critical gap remains: many analyses neglect the integration of commercially available, off-the-shelf components into the design process. Bottom-up strategies are well-suited for developing general-purpose robots with broad functionalities, while top-down approaches excel at creating robots specifically tailored for particular applications (17). The top-down paradigm initiates the design process with a clear focus on the final application, resulting in a more streamlined design space. The ~~Addressing this,~~ recent studies (18) highlight the efficacy of top-down approaches in mitigating cyclic dependencies, particularly beneficial for the efficient design of mechatronic systems characterized by intricate interdependencies. ~~Another pertinent contribution in this domain comes from a study on the optimization of reconfigurable collaborative robots, emphasizing modularity and structural considerations~~ Another relevant contribution to this field comes from a study that optimized reconfigurable collaborative robots, focusing on the importance of modularity and structural design (19). This innovative top-down approach draws inspiration from the V-model employed in the automotive and aerospace industries, as introduced in (20). ~~The optimization strategy revolves around enhancing the cost-effectiveness of development by optimizing the physical structure, consequently yielding savings in manufacturing materials. Additionally, a significant reduction in development time is achieved by capitalizing on established modular interfaces instead of customizing interfaces for each module, as detailed in~~ This optimization strategy aims to improve development cost-effectiveness by optimizing the physical

structure, which in turn reduces manufacturing material costs. Additionally, leveraging established modular interfaces significantly cuts down on development time compared to customizing interfaces for each module, as detailed in (19). These contemporary studies collectively underscore the evolving landscape of design processes, shedding light on the nuanced benefits of incorporating off-the-shelf components within a top-down framework. These recent studies together highlight the changing nature of design processes. They reveal the valuable advantages of integrating off-the-shelf components within a top-down approach.

Like any robotic system, lightweight manipulators adhere to basic design principles and employ specific components to achieve their lightweight characteristics (21). The IDJ or Direct Drive Joint (DDJ) incorporates frameless BLDC Motors and Strain Wave Gears, distinct from conventional manipulators. Additionally, lightweight manipulators utilize differently designed links made from lightweight materials. Over the past two decades, researchers have proposed various drive unit designs suitable for lightweight robots (21-24). The design constraints imposed by the hollow shafts of drive units and of links connecting the drive units to form the kinematic chain, require specific component choices. For example, both the flexspline of the strain wave gear and the lightweight structure of the robot contribute to its overall mechanical flexibility, necessitating the consideration of dynamic coupling among multiple drive units through lightweight links. Consequently, optimizing the subcomponents of drive units and links becomes a primary objective in the designing and manufacturing of lightweight robots. Moreover, considering the extensive experience in the development of robotic manipulator components (25, 26), it is prudent to leverage this existing knowledge to narrow down the design space. Furthermore, researchers have also proposed schemes to optimize the selection of off-the-shelf motors, both AC Servos (27) and BLDC Motors (28), that can enhance the performance of already built manipulators. These studies analyze the dynamic behavior of the robotic system for optimizing the choice of motors using cost functions for energy consumption, trajectory tracking error, and total weight of the motors. Additionally, another research (29) showcases a method for testing drive-transmission couples suitable for following a defined motion. The analysis considers each pair separately without integrating them into a multi-DOF coupled system. Therefore, while this method enables the individual performance evaluation of drive-transmission couples, it does not provide analysis for situations where such pairs are joined together through a multi-DOF system. This highlights the need for comprehensive approaches that account for the dynamic interactions between coupled components within a multi-DOF robotic system.

Like any robotic system, lightweight manipulators adhere to basic design principles and employ specific components to achieve their lightweight characteristics (30). The IDJ or Direct Drive Joint (DDJ) incorporates frameless BLDC Motors and Strain Wave Gears, distinct from conventional manipulators. Additionally, lightweight manipulators utilize differently designed links made from lightweight materials. Over the past two decades, researchers have proposed various drive unit designs suitable for lightweight robots (22-24, 30). (27, 28)The design constraints imposed by the hollow shafts of drive units and of links connecting the drive units to

Formatted: Font: +Body (Calibri), 12 pt, Font color: Auto, Pattern: Clear

~~form the kinematic chain, require specific component choices. For example, the flexspline of the strain wave gear and the lightweight structure of the robot introduce mechanical flexibility, necessitating the consideration of dynamic coupling among multiple drive units through lightweight links. Consequently, optimizing the subcomponents of drive units and links becomes a primary objective in the design and manufacturing of lightweight robots. Moreover, considering the extensive experience in the development of robotic manipulator components (25, 26), it is prudent to leverage this existing knowledge to narrow down the design space.~~

~~To evaluate the performance of interconnected systems that incorporate multiple drive units and links, simulation tools are essential. However, the lack of simulation tools capable of simulating complete robotic systems with integrated drive joints forces developers to physically test and validate certain components. The discrepancy arises because designers often choose to simulate idealized systems or systems based on various assumptions in existing robot simulators~~ Evaluating the performance of interconnected systems with multiple drives and links requires simulation tools. Unfortunately, current tools often lack the capability to simulate entire robotic systems with integrated drive joints, forcing developers to physically test and validate certain components. This gap arises because existing robot simulators typically allow designers to only simulate idealized systems or systems based on various assumptions (31, 32). ~~There is a need to model complex systems using available hardware. For instance, when determining suitable gearing systems for a specific speed reduction task, a design engineer typically suggests gear specifications through calculations and simulations. However, these specific gearing systems might not be procurable or manufacturable. Conversely, it would be advantageous to explore the available gearing systems suitable for lightweight robotic manipulators, model them, and adapt other parts of the system to achieve the desired speed reduction. This concept extends beyond gearing systems to encompass motors, sensors, bearings, motor drives, and the robot structure as well.~~ Modeling complex systems with readily available hardware is crucial. For example, when designing a speed reduction mechanism, engineers typically rely on calculations and simulations to identify suitable gears. However, these simulations might not account for real-world limitations like manufacturability or available parts. A more advantageous approach would be to explore commercially available gear options suitable for lightweight robots, model their performance, and then adapt other system components to achieve the desired speed reduction. This concept applies beyond gears – it extends to motors, sensors, bearings, motor drivers, and even the robot structure itself.

~~In the realm of industrial robotics, decades of design and manufacturing experience have allowed for the identification of off-the-shelf components that can be utilized to construct high-performance functional manipulators. Adopting a counter approach, where the available components shape the design space, would be highly desirable if comprehensive simulated models of all commercially available components were readily accessible.~~ Industrial robotics has benefited for decades from the use of readily available components to build high-performance manipulators. A counter-intuitive approach, where existing components influence the design process, could be even more desirable. However, this would require easy access to

comprehensive simulated models of all commercially available components. Unfortunately ~~Regrettably~~, the current landscape does not provide an extensive repertoire of simulated models, and even when ~~a few~~ such models do exist, there is a notable absence of a *unified and scalable platform* capable of testing the combinations of ~~different~~ subcomponents employed in the fabrication of critical elements, such as a single joint within a robotic manipulator or the manipulator as a complete system made up of several subcomponents.~~.~~

Fig 3 illustrates the comprehensive workflow of the proposed scheme. The end-user provides the ~~system~~ initial specifications of the robot, which in the current stage are limited to the degrees-of-freedom, reach of the manipulator, payload carrying capacity, and the maximum workspace speed of the end-effector.~~requirements, which~~ These specifications serve as the basis for component selection, leading to the creation of a Computer Aided Design (CAD) model of the robot. This CAD model is subsequently imported into the Simulink environment, where it is subjected to predefined trajectories. Based on the evaluation of position, velocity, and torque tracking performance, frameless BLDC motors and/or strain wave gears may be substituted, if necessary. Once satisfactory performance is achieved in the simulation, that is the robotic manipulator successfully follows the reference trajectories with correlation coefficient greater than 0.95, the robot is fabricated and assembled utilizing the commercially available components identified during the simulation phase. To complete the entire process, a comparative analysis is performed ~~conducted~~, contrasting the operational performance of the manufactured robot with that of the simulated robot. This research introduces a novel design and development framework for lightweight robots. However, opportunities remain ~~exist~~ for further optimization of each subcomponent, which will be a focus of future research.

Formatted: Font: +Body (Calibri), 12 pt, Font color: Auto

Formatted: Font: +Body (Calibri), 12 pt, Font color: Auto

Fig 3: Comprehensive workflow of the proposed scheme.

Methodology for Implementation of the Proposed Scheme

~~Developing a comprehensive simulation model that encompasses all components of a real-life IDJ, presents a complex undertaking. However, it becomes evident that the frameless BLDC motor and the gearing system are the two major components. Consequently, detailed models were constructed for these components, while simplified approximations were employed for smaller subcomponents like position encoders and bearings. This approach is logical as the inertia of the IDJ is primarily attributed to the BLDC motor and the gearing system, with the BLDC motor also consuming most of the electrical power. Thus, both the electrical and mechanical characteristics of the IDJ are primarily dictated by these two key components.~~ Building a simulation model that captures all aspects of a real-world Integrated Direct Drive Joint (IDJ) is a complex task. However, the frameless BLDC motor and the gearing system emerge as the critical components influencing the IDJ's behavior. Therefore, detailed models were created for these elements, while less complex representations sufficed for smaller components like position encoders and bearings. This prioritization is justified because the BLDC motor and gearing system

dominate the IDJ's inertia and electrical power consumption. Consequently, these two key components primarily define the IDJ's electrical and mechanical characteristics.

The development of models for the involved subcomponents necessitates consideration of several aspects. For instance, a motor requires modeling of both its electrical and mechanical attributes, whereas a gearing system pertains solely to mechanical aspects. However, different types of gearing systems exhibit distinct behaviors. For example, a planetary gear system lacks flexibility within its internal gears but possesses measurable backlash. On the other hand, a strain wave gear introduces flexibility due to its flex spline but does not exhibit backlash. Backlash and rigidity are just a few of the differing characteristics between these two types of gearing systems. Therefore, a comprehensive modeling of a gearing system requires the consideration and optimization of various parameters based on the specific requirements. However, to streamline the process, it is practical to focus solely on modeling the parameters of commercially available hardware components For instance, planetary gear systems offer limited internal gear flexibility but have measurable backlash. In contrast, strain wave gears introduce flexibility through their flex spline and eliminate backlash. Backlash and rigidity are just two key differences between these gear types. Therefore, a comprehensive gear system model requires considering and optimizing various parameters based on specific needs. However, to simplify the process, focusing solely on modeling commercially available components' parameters is a practical approach, without concerning about the validity and optimization of such parameters. bypassing the optimization step entirely. Consequently, developers can expedite the process by leveraging existing hardware components and utilizing their models within the simulation environment, effectively bridging the gap between simulation and actual hardware.

The subsequent discussion elaborates on the potential components that an IDJ may encompass. However, it is worth noting that certain components may be omitted in practice without compromising performance, albeit at the expense of non-performance-related features. Moreover, the simulated models utilize parameters sourced from component datasheets, which are typically freely available on the manufacturer's website.

Frameless BLDC Motor Model

Extensive research has been conducted on developing simulation models for various types of motors, including BLDC motors. Given the objective of this study to establish an effective combination of motor models, strain wave gears, and approximate other components and parameters, there is no need to reinvent the wheel. Hence, standard BLDC motor models, "Significant research already exists on simulation models for various motors, including BLDC motors. Since this study aims to find an effective combination of motor models, strain wave gears, and simplified models for other components, there's no need to develop entirely new models from scratch. Therefore, we leveraged standard BLDC motor models e.g. AC7 block of Simulink MATLAB, have been utilized and adapted to create models for commercially available frameless

~~BLDC motors~~, and adapted them to create models for specific commercially available frameless BLDC motors.".

In addition to the BLDC motor, the drive system used to operate it plays a crucial role. The choice of drive system determines the required feedback and the level of control it can exert over the motor's motion. Typically, commercially available BLDC motor drives implement speed control through stator current control, employing a position sensor such as a rotary encoder or a hall sensor. The generalized mechanical system dynamics are represented by:

$$T_e = J\frac{d}{dt}\omega_r + F\omega_r + T_m \qquad (2)$$

Where T_e represents the electromagnetic torque, J the inertia, ω_r the rotor speed, F the viscous friction, and T_m the load torque. These kinds of drives have an inner current loop that will take in the commanded torque and utilize the torque constant of the motor to generate suitable stator phase currents. Whereas the outer speed control loop takes in the load or applied torque and reference speed to figure out the electromagnetic torque as per the mechanical system's dynamic equation. Furthermore, commercial motor drives implement Proportional-Integral (PI), Proportional-Derivative (PD) or Proportional-Integral-Derivative (PID) controller for maintaining the desired speed-torque characteristics. These controllers are pre-tuned or may easily be ~~auto~~tuned using the ~~damping factor and the desired response time~~manufacturer's defined method and provided scheme. Fig 4 shows the block diagram of the overall control scheme for commercially available motor drives.

Fig 4: General control scheme implementing closed loop control at all three stages, that is, torque (current), velocity, and position.

~~In this study, BLDC motor controllers incorporating PI controllers at all three levels were employed: the innermost current loop, velocity loop, and outermost position loop. The inclusion of an integrator in servomechanisms ensures zero steady-state error and helps to mitigate noisy signals commonly encountered in lightweight robotic manipulators, where weak sensor signals are in close proximity to high-powered BLDC motors. Moreover, the choice of using PI controllers only is further supported by the fact that all different types of hardware motor controllers used in this study employ only PI controllers to control the BLDC motor. The PI controllers of the hardware motor controllers were tuned according to the manufacturer's specifications~~ This study employed BLDC motor controllers with PI controllers at all three levels: the current loop (innermost), velocity loop, and position loop (outermost). The integrator in PI controllers eliminates steady-state errors, which is particularly beneficial for lightweight robotic manipulators. These manipulators often experience noisy signals due to weak sensor signals being close to powerful BLDC motors. Additionally, all commercially available hardware motor controllers used in this study relied solely on PI control, further supporting our choice. The PI parameters within these hardware controllers were adjusted according to the manufacturer's recommendations. and the trajectory following of a certain actuator before and after the tuning

is shown in the hardware experimentation section, while the simulation-based motor controllers were autotuned using the damping factor and desired response time, as outlined in (33). The damping factor is defined as follows:

$$\zeta = \frac{\omega_n K_p}{2K_i} \quad (3)$$

If $\zeta < 0.69$, then natural frequency is defined as:

$$\omega_n = \frac{-1}{\zeta \times T_{nl}} \log(0.05 \times \sqrt{1-\zeta^2}) \quad (4)$$

Where if $\zeta \geq 0.69$, then natural frequency is defined as:

$$\omega_n = \frac{0.9257}{T_{nl}} e^{1.6341 \times \zeta} \quad (5)$$

Strain Wave Gear Model

In this research, the modeling of strain wave gears encompasses several crucial parameters that can be sourced from either the manufacturer's datasheet or the CAD model. These include weight, moment and product of inertia, stiffness, damping constant, nominal torque, and nominal speed. These parameters form the foundational framework for the accurate representation of strain wave gears within the simulation environment, ensuring a comprehensive and precise analysis of their performance characteristics.

While most parameters can be directly extracted, the moment and product of inertia are obtained from the manufacturer-provided CAD model. The damping constant is influenced not only by the grease used in the gearing system but also by the friction in the bearings and overall component alignment. Therefore, an empirical determination of the damping constant was performed for one of the manufactured joints to account for manufacturing imperfections and friction. Standardizing the manufacturing process, materials, assembly processes, strain wave gear grease, and bearings will improve the approximation of the damping constant. The determined damping constant was incorporated into the simulated model, present in the component library, to capture its effects.

Robot Structure

For modeling the structure of the robotic manipulator, SolidWorks 2021 was employed in this research. All necessary parts were modeled, and analysis was performed to establish the validity. IDJ was modeled using the supplier-provided CAD models of the included components, which were the frameless BLDC motor, strain wave gear, optical encoder, bearings, and joint housing that will encase all the components. Moreover, the links and the connection flanges were modeled and passed through stress-strain analysis for establishing the validity of the model subjected to the worst-case loading. For links and joint housings, we used Aluminum 6061-T6 material because of its strength and lightweight, while for connection flanges we used Alloy Steel

~~having yield strength of~~ We modeled the robot structure with SolidWorks ~~2021 .~~2021. All necessary parts were meticulously modeled and analyzed for validity. The Integrated Direct Drive Joint (IDJ) was constructed using supplier-provided CAD models of its components: the frameless BLDC motor, strain wave gear, optical encoder, bearings, and the joint housing that encases them. Additionally, the links and connection flanges were modeled and subjected to stress-strain analysis to ensure the model's validity under worst-case loading conditions. Aluminum 6061-T6, known for its strength-to-weight ratio, was chosen as the material for the links and joint housings. Meanwhile, the connection flanges utilized high-strength Alloy Steel with a yield strength of $6.2x10^8$ N/m^2. The factor of safety of the designed ~~for~~ Aluminum links is at least 2.4, while for flanges its 4.3. Fig 5 shows stress analysis figure of one of the links being subjected to a maximum of $1.158x10^8$ N/m^2 stress, while Aluminum 6061-T6 has yield strength of $2.75x10^8$ N/m^2, hence achiev~~eing~~ 2.4 factor of safety ~~of 2.4~~. These values of the factor of safety ensure that the structure can easily withstand higher loading, hence increasing the structural integrity and life of the manipulator. Some covers which were not ~~load~~ bearing any load, were later 3D printed, and were excluded ~~hence doesn't need any~~ from the analysis.

Fig 5: Stress Analysis of one of the links showing maximum stress being applied on it in worst case.

The CAD model of the robot's structure generated in SolidWorks was ~~then~~ imported into MATLAB's environment for simulation. The CAD model considered the following parameters:

- Robot configuration
- Link weights
- Link moment and product of inertias
- Link lengths

Fig 6 illustrates one of the designed links and its integration with the joints to form the complete CAD model of the 5 DOF robot.

Fig 6: Exploded view of the 5 DOF robot showing Links, Joints, Connection flanges, and 3D printed covers.

~~The robotic manipulator consists of additional subcomponents within the integrated drive joint that have smaller weight and moment of inertia. These components, listed below, can be grouped together, and represented as a single model by combining their net weights and inertias, as their individual effects on the dynamics are relatively minor.~~ The integrated drive joint of the robotic manipulator includes additional subcomponents with minimal weight and ~~moment~~moments of inertia. These components, listed below, can be grouped and represented as a single model by combining their net weight and inertia. Their individual influence on the overall dynamics is negligible due to their small ~~size~~size.

Other components

~~In addition to the previously mentioned components, there are various other components that may or may not be included in an integrated drive joint~~ Several other components, like miniaturized circuit boards, position sensors, bearings, torque sensors, brakes, and housings, might be included in an integrated drive joint (34). ~~These components, such as the miniaturized circular circuit board, position sensor, bearings, torque sensor, brake, and housing, have significantly lower weights and moment of inertias compared to the BLDC motor, strain wave gear, and robot's structure. For analysis, these components are combined into a single unit for simplicity. The forthcoming section elaborates on the aspects considered for modeling and subsequent manufacturing of the integrated drive joint.~~ These components typically have significantly lower weights and moments of inertia compared to the BLDC motor, strain wave gear, and robot structure. To simplify the analysis, these components are grouped together as a single unit. The next section details the considerations for modeling and manufacturing the integrated drive joint.

Simulation Model Flow

~~The direct drive joint model was developed by individually modeling and combining commercially available subcomponents. The model includes a frameless BLDC motor, strain wave gear, incremental position encoder, hollow shaft, bearings, joint housing, and miscellaneous small parts. The electrical aspects of the frameless BLDC motor were modeled in Simulink MATLAB using the Brushless DC Motor Drive (AC7) block, which represents a standard current-controlled drive for BLDC motors with closed-loop speed control. The mechanical aspects of the motor and strain wave gears were modeled in SolidWorks~~ The direct drive joint model was built by individually modeling and assembling commercially available subcomponents. These subcomponents include a frameless BLDC motor, strain wave gear, incremental position encoder, hollow shaft, bearings, joint housing, and various minor parts. The electrical behavior of the BLDC motor was modeled in Simulink MATLAB using the Brushless DC Motor Drive (AC7) block, which simulates a standard current-controlled drive system for BLDC motors with closed-loop speed control. The mechanical aspects of the motor and strain wave gear were modeled within SolidWorks.. The modeled parts make up the component library from which suitable parts are to be selected to make up the desired robotic manipulator. Table ~~1~~ 2 presents the parameters used to model the frameless BLDC motor, extracted from commercially available datasheets. Furthermore, to account for potential manufacturing inaccuracies, up to 10% random variations were introduced in the weight, inertia, and stiffness parameters of the simulated direct drive joint and robot's structure. These variations compensate for possible deviations that may arise during the actual manufacturing process, such as material and machining differences and assembly imperfections. Additionally, an empirically determined damping constant was incorporated to model frictional forces. A test joint was manufactured to evaluate manufacturing imperfections and determine frictional losses, which were then included in the simulated models present in the developed library. All joints of the 5-DOF manipulator were configured with a damping constant of 0.035 Nm/rad/s. Fig 7 illustrates a snapshot of a test joint.

Fig 7: IDJ Testing Setup

The developed simulator includes eight different frameless BLDC motors from T-Motor and Maxon, allowing for modeling of various motors without manufacturer dependence.

Table ~~1~~2: List of BLDC Motor Parameters that were used for modeling the commercially available motors. Sample of three different BLDC Motors are shown.

Sr. #	Parameter	Unit	TMotor RI50	TMotor RI80	Maxon 588849
1	Resistance	Ohm	0.673	0.196	0.109
2	Inductance	mH	0.474	0.364	0.0665
3	BEMF Flat Area	rad	1.197	1.197	0.761
4	Torque Constant	mNm/A	99	127.3	80.7
5	Speed Constant	rad/s/V	10.11	7.85	12.36
6	Robot Inertia	$g.cm^2$	3500	6000	5300
7	Poles	-	7	7	11
8	Nominal Torque	mNm	500	1450	1010

Similarly, several parameters of strain wave gear were used to model its effect in the direct drive joint, out of which the gear ratio is defined in the motor block. Apart from gear ratio, the weight, moment and product of inertias, stiffness, nominal torque, and nominal speed parameters were defined and used in different blocks. Furthermore, the simulated direct drive joint model includes the weights and inertias of position encoder, bearings, joint housing, and the hollow shaft through which the power and communication wires are routed.

Fig ~~7~~ 8 shows the section view of the CAD model of the simulated direct drive joint.

Fig ~~7~~8: Section view of the CAD model of the assembled Integrated Drive Joint

Later, the simulated integrated drive joint was installed in the simulated robot structure, and the manipulator was driven on a defined trajectory. The position, velocity, and torque requirement graphs were plotted to establish the suitability of the simulated direct drive motor joint.

Manipulator Model

To evaluate the simulated integrated drive joint, a robotic manipulator with specifications defined in the Simulation Experimentation section was simulated. ~~it was integrated into a 5-DOF robotic manipulator model. This manipulator model was meticulously constructed in SolidWorks and seamlessly imported into MATLAB's Multibody environment.~~ The development of the manipulator's model considered critical parameters, including robot configuration, link weights, link moment, product of inertias, and link lengths. These considerations were pivotal in ensuring

the accuracy and effectiveness of the simulated manipulator's performance under various conditions.

Table ~~2~~ 3 presents the parameters of the robot links and joints used to generate the simulation model. These parameters were obtained from the CAD model, which considers the commercially available material, its properties, and the machining processes involved. While importing the model from SolidWorks 2021 to the Simulink environment, the dimensions and shape of the links were preserved. However, the shape and size of the joints were approximated by a point mass while maintaining all other parameters.

Table ~~2~~3: List of Parameters used for modeling the structure of the robot in Simulink Environment.

Sr. #	Manipulator Link		Manipulator Joint	
	**Parameter**	**Unit**	**Parameter**	**Unit**
1	Size and Shape	mm	Point Mass	-
1	Mass	kg	Mass	kg
2	Center of Mass	mm	Center of Mass	mm
3	Moments of Inertia	$g.cm^2$	Combined Moments of Inertia	$g.cm^2$
4	Products of Inertia	$g.cm^2$	Combined Products of Inertia	$g.cm^2$

Individual links of the robotic arm were imported into the Multibody environment and were assembled in the defined configuration to build the complete structure. Fig ~~8~~ 9 shows the Simulink model for building the robotic arm in MATLAB.

Fig ~~8~~9: Simulink SimScape Multibody model for building 5 DOF Robotic Arm

Section III

Simulation Experimentation

Using the steps outlined in Fig 3, the experimentation was initiated by specifying the initial specifications of the desired robot. The DOF were chosen to be 5, reach of about 1.1 m, with a payload capacity of 3 kg and as we are only focusing on developing lightweight robots then maximum speed of the end-effector was confined to 1 m/s. This value of speed ensures that the designed robot doesn't breach the safety guidelines laid down in ISO/TS-15066 (35). In the experimentation phase, direct drive joints featuring various commercially available frameless BLDC motors were integrated with the 5-DOF manipulator model made of manually selected robot links from the produced library. Trapezoidal velocity trajectories were generated as reference trajectories for different scenarios, and the simulated model was driven on these trajectories to evaluate the performance of the direct drive joints. Fig ~~9~~ 10 provides a snapshot of the simulation running on a specific trajectory.

Fig 910: Snapshot of simulation of 5 DOF manipulator on a given trajectory.

To account for potential manufacturing inaccuracies, up to 10% random variations were introduced in the weight, inertia, and stiffness parameters of the simulated direct drive joint and robot's structure. These variations compensate for possible deviations that may arise during the actual manufacturing process, such as material and machining differences and assembly imperfections. Additionally, an empirically determined damping constant was incorporated to model frictional forces. A test joint was manufactured to evaluate manufacturing imperfections and determine frictional losses, which were then included in the simulated model. All joints of the 5-DOF manipulator were configured with a damping constant of 2 Nm/deg/s. Fig 10 illustrates a snapshot of a test joint.

Fig 10: IDJ Testing Setup

The simulation was conducted using the finalized parameters and SimScape Multibody model. The SimScape Multibody simulates system dynamics using a range of solvers, which can be variable or fixed steps. As we are going to test the physically manufactured robot on EtherCAT protocol, we used a fixed step solver with step-size of 0.1 ms. The EtherCAT protocol runs at a sampling time of 1 ms, that is why we had to run the simulation at an even smaller time step. The type fo the solver was left to Simulink by allowing it to automatically select the solver type. The results were analyzed to determine if the simulated joints successfully followed the reference trajectories. Since a robotic manipulator consists of multiple joints and links, the focus was on analyzing the coupled dynamics of the entire structure rather than individual joint performance. Assessing the performance of each joint when interconnected through the robot's structure was deemed more meaningful. Moreover, For industrial robotic manipulators, primary sources of disturbance include environmental interaction, unmodeled dynamics, and gravitational effects The results aimed to verify if the simulated joints could precisely track the desired reference trajectories. For a robotic manipulator with interconnected joints and links, evaluating the coupled dynamics of the entire structure provided a more meaningful assessment than analyzing individual joint performance. In industrial settings, robotic manipulators encounter various disturbances from environmental interactions, unmodeled dynamics, and gravitational effects (36). All these sources of disturbance are encountered and treated at the system level. Fig 11 depicts the system level Simulink model used to simulate the complete system. These disturbances are addressed at the system level. Figure 11 illustrates the system-level Simulink model used to simulate the complete robotic system.

Fig 11: System level Simulink Model for simulating the robotic manipulator and its joints.

The system model shown in Fig 11 outlines the simulation flow. A reference trajectory was generated in joint space and applied to the Multibody model of the robot. The model followed the trajectory, while position, velocity, and torque sensors captured corresponding signals. The sensed positions and torques were then used to control the BLDC motors through a PI-based control system. Joints that were capable of accurately following the desired position and torque profile exhibited minimal deviation between the output and reference signals. To quantitatively assess the similarity between the reference and simulated data, Pearson's Correlation Coefficient was calculated using the provided equation. Additionally, visual comparisons were also conducted.

$$r = \frac{\sum(x_i - \bar{x})(y_i - \bar{y})}{\sqrt{\sum(x_i - \bar{x})^2 \sum(y_i - \bar{y})^2}} \qquad (5)$$

Where r = Pearson's Correlation Coefficient, x = value of the first variable, $\bar{x}$ = mean value of the first variable, y = value of the second variable, and $\bar{y}$ = mean value of the second variable.

The Pearson's Correlation Coefficient remains a crucial is a critical metric used to assess the degree of similarity between the reference and simulated torque profiles. It serves as an indicator of the suitability of the direct drive joint at its installed position. A low correlation coefficient value suggests an unsuitable joint, while a value closer to one indicates that the joint is well-suited for its installed position within the robot. The correlation coefficient is specifically calculated for the torque profile, as the correlation coefficients of the position and velocity profiles are dependent on the coefficient of the torque profile. Fig 12 demonstrates the response of an underpowered joint, highlighting the significance of adequate power for joint performance.

Fig 12: (a) Position, (b) Velocity, (c) Torque Reference vs Simulation Data of Joint 2 when an underpowered joint was installed at this location.

The underpowered joint may cause resulted in significant deviations in position and velocity responses from the reference trajectory due to insufficient torque. due to its inability to provide the required torque. The generated torque fell short of desired value, reaching around 50 Nm compared to the required 70 Nm, leading to a failure in following the trajectory. The correlation coefficient of 0.2593 quantitatively indicated a mismatch between the required and generated torque. To address this issue, we iterated the simulation using different integrated drive joints, gradually moving from low to high power joints. This IDJ selection method is currently manual with the aim of being automated in future. Currently the criteria for selection were to minimize the total weight of the joints and achieve enough torque capability to drive the robot on the reference trajectory with a correlation coefficient greater than 0.95. The selection process began with the last joint and moved backward, considering the impact of later joints on the earlier ones. This selection methodology aimed to helps identify optimal joints for each position. Intelligent selection methods will be implemented as the library of simulated integrated drive joints expands, currently including frameless BLDC motors and strain wave gears from Maxon, TMotor, and Laifual Drive. Furthermore, we successfully resolved the underpowered

joint issue by substituting it with the selecting suitable direct drive joints and tested the robot on a pick-and-place trajectory. Trajectories for position, velocity, and torque were obtained for all five joints of the 5-DOF robot. Through visual inspection, point-by-point comparison, and Pearson's correlation coefficient analysis, we confirmed that the joint trajectories of the simulated model closely followed the reference trajectories, surpassing the defined acceptable level of correlation coefficients greater than 0.95.

For the pick and place application, the reference versus simulated data for all the joints is shown in Figs 13 to 17.

Fig 13: Reference vs Simulation Data of Joint 1's (a) Position, (b) Velocity, and (c) Torque.

Fig 14: Reference vs Simulation Data of Joint 2's (a) Position, (b) Velocity, and (c) Torque.

Fig 15: Reference vs Simulation Data of Joint 3's (a) Position, (b) Velocity, and (c) Torque.

Fig 16: Reference vs Simulation Data of Joint 4's (a) Position, (b) Velocity, and (c) Torque.

Fig 17: Reference vs Simulation Data of Joint 5's (a) Position, (b) Velocity, and (c) Torque.

The simulation results demonstrate that the simulated integrated drive joint successfully produces the reference trajectory in all cases. The torque difference graph reveals a disparity between the required and simulated torque, but this difference does not result in noticeable deviations in the position or velocity trajectories due to the robustness of the PI controller. Additionally, Table 3 4 provides the Pearson's Correlation Coefficients for all joints, quantifying the degree of match between the reference and simulated trajectories.

Table 34: Pearson's Correlation Coefficient between Reference and Simulated Torque Profiles of all joints for Pick and Place Trajectory.

Parameter	Joint 1	Joint 2	Joint 3	Joint 4	Joint 5
Correlation Coefficient	0.9969	0.9984	0.9982	0.9992	0.9963

Formatted Table

The high correlation coefficients obtained in the simulation experiments highlight the effectiveness of the simulation tool and the chosen motor and harmonic drive combinations in achieving accurate and reliable robot motion control. These results demonstrate that the simulated robot performance closely aligns with the intended reference trajectories, which is crucial for ensuring precise and consistent robot motion.

Several sources of noise and disturbances including EMI, sensor (encoder) noise, input and output disturbances can cause the manipulator to deviate from an ideal trajectory in ~~the real~~real-life. We analyzed how adequately our PI controller would reject sources of noise disturbances in real-life by lumping these at the output of the speed controller, in the form of a single noise source. Specifically, this is an additive white gaussian noise with unit variance, resulting in a signal-to-noise ratio (SNR) of 15 dB, which is in close agreement with (Fig.22) noise levels observed in real experiments. Fig 18 depicts the responses of position, velocity, and torque tracking for one of the joints, joint 3, subject to noise/disturbance.

Fig 18: Reference vs Simulation data of Joint 3 after introducing disturbance, (a) Position, (b) Velocity, and (c) Torque.

In Fig 18, although there is distortion in the speed and, consequently, in the torque, the PI controller implemented in the position loop adeptly mitigates the effects of the introduced disturbance. This reinforces the robustness of the controller.

This finding carries significant implications for practical applications, as it suggests that physically manufactured robots using the same integrated drive joints and control system can potentially achieve similar performance. The subsequent section presents the results of fabricating the robot based on the integrated drive joints recommended by the simulation studies.

Section IV

Hardware Experimentation

~~As per the results obtained from the simulation experimentation, integrated drive joints were manufactured using the suggested commercially available components. Moreover, to meet the latest industrial standards, BLDC motor controllers from Nanotec® having EtherCAT communication capability were used to drive the BLDC motors. Five such joints were manufactured along with the manipulator’s structure. Assembling all the subsystems produced a 5-DOF manipulator, shown in Fig 19, being controlled via EtherCAT enabled BLDC motor controllers.~~ Based on the simulation results, integrated drive joints were built using the recommended commercially available components. To comply with modern industrial standards, Nanotec® BLDC motor controllers with EtherCAT communication capabilities were chosen to drive the BLDC motors. Five such joints were manufactured alongside the manipulator's structure. The complete assembly resulted in a 5-DOF manipulator, pictured in Fig 19, controlled via EtherCAT-enabled BLDC motor controllers.

Fig 19: Manufactured 5DOF Robot with Integrated Drive Joints performing a pick and place task.

We used Plug & Drive software, provided by Nanotec®, for ~~to~~ tuning~~e~~ the PI control loops of the motor controllers. This~~e~~ motor controller, similar to ~~just like our~~ ~~simulated motor controller,~~ implements the PI control loops on three levels, that is, the inner most on torque, then on velocity, and the outer most on position. The position and velocity following response of one of the motor controllers before and after tuning is shown in Figs 20 and 21.

Figs 20 and 21: Before and After tuning response of position (left) and Velocity (right) of one of the joints. The x-axis shows time in milliseconds, whereas y-axis shows the position and velocity in motor controller units.

The physical~~ly~~ prototype ~~developed~~ robot was ~~executed~~ programmed to follow ~~on~~ the ~~same~~ trajectory used in the simulation, and the corresponding torque, velocity, and position data from the integrated drive joints were collected. The collected data was ~~then~~ compared with the simulated data for the pick and place joint space trajectory, as shown in Figs 22 to 26. It is important to note that the time constant of a geared joint prevents any noticeable movement of the output shaft due to current spikes. However, for experimentation purposes, the torque data was obtained from the current sensor in the motor controller, at a sampling rate of 1000 Hz, and passed through a moving average filter with a window size of 15 samples, approximately equal to 0.015 seconds in time. This moving average filter emulates the damping effect of the geared joint.

Fig 22: Simulated versus Experimental data of Joint 1's (a) Position, (b) Velocity, and (c) Torque.

Fig 23: Simulated versus Experimental data of Joint 2's (a) Position, (b) Velocity, and (c) Torque.

Fig 24: Simulated versus Experimental data of Joint 3's (a) Position, (b) Velocity, and (c) Torque.

Fig 25: Simulated versus Experimental data of Joint 4's (a) Position, (b) Velocity, and (c) Torque.

Fig 26: Simulated versus Experimental data of Joint 5's (a) Position, (b) Velocity, and (c) Torque.

Once again, visual and Pearson's correlation coefficient were used to establish the similarity of the trajectories coming from the simulated model and the actual robot. Table ~~4~~ 5 lists the Pearson's Correlation Coefficient for torque profiles of all joints.

Table ~~4~~5: Pearson's Correlation Coefficient between Simulated and Actual Torque Profiles of all joints for Pick and Place Trajectory.

Parameter	Joint 1	Joint 2	Joint 3	Joint 4	Joint 5
Correlation Coefficient	0.9966	0.9872	0.9882	0.9888	0.9895

Although the correlation coefficients have slightly decreased, they remain within an acceptable range as the position and velocity trajectories are still being accurately followed. However, the mismatches in the torque trajectories require further investigation. The torque impulses observed in the physical prototype ~~ly manufactured robot are natural and result~~ come from the curre nt sensing on ~~nt readings taken from~~ the motor controller. These impulses are within the current limits of the BLDC motors and do not cause any ~~damage or~~ deviation in the required position trajectory. Additionally, the average torque deviation from the simulated model remains below 5 Nm for most of the time, which is a small percentage of the nominal torque capability of the integrated drive joints. This additional torque compensates for unmodeled dynamics, friction, and manufacturing variations. Simulation tools capable of ~~- are useful for~~ predicting the robot behavior in an ideal world ~~but~~ may fail to account for the performance under ~~cannot fully account for~~ real-world imperfections ~~complexities~~ such as friction and manufacturing variations. However, the position and velocity following performance of the robot is still below ~~under~~ acceptable level due to the disturbance rejection and noise cancellation properties of the PI controllers used. These results emphasize the importance of considering real-world complexities and the need for ongoing calibration and optimization. We aim to f~~F~~urther investigate~~ion~~ this problem ~~will be conducted~~ to minimize th~~e~~ simulation-experiment performance disparity. ~~impact of these mismatches in future work.~~

Apart from visual comparison and correlation coefficient between the simulated and the actual trajectories, trajectory tracking error was also calculated to further establish the trajectory following capability of the physically developed manipulator. We calculated Root Mean Squared Error (RMSE) for all five joints using equation 6, given below.

$$RMSE = \sqrt{\frac{1}{N}\sum_{i=1}^{N}\left(x_{ref,i} - x_{measured,i}\right)^2} \qquad (7)$$

Table 6 gives the RMSE of joint space position, velocity, and torque trajectories. Position and velocity RMSE signifies the position and time tracking efficiency of the developed robot, whereas torque RMSE once again suggests that the deviation in torque is a result of unmodeled dynamics. The maximum RMSE of torque is for joint 2, which is 5.4244 Nm, and if considered along with Fig 23, then this is around 10% of the total torque output for the desired trajectory. Moreover, the IDJ installed at joint 2 can produce torque up to 145 Nm, therefore, if the RMSE is seen in comparison to the maximum torque capability of the joint then it is only around 3.74%.

Table 6: Root Mean Squared Error of joint space position (rad), velocity (rad/s), and torque (Nm) trajectories calculated using the simulated and measured trajectories of physically developed 5 DOF manipulator.

Trajectory	Joint 1	Joint 2	Joint 3	Joint 4	Joint 5
Position (rad)	0.0018	0.0086	0.0042	0.0091	0.0046
Velocity (rad/s)	0.0062	0.0223	0.0134	0.0238	0.0125
Torque (Nm)	1.8585	5.4244	2.3571	3.7471	1.5893

Formatted Table

Furthermore, the repeatability of the developed 5-DOF robot prototype was calculated as per the procedure described in ISO 9283:1998 (37), defined specifically for industrial robots. ~~Dial~~A dial gauge having positional resolution of 0.01mm was used to measure the position of the end-effector of the robot. Five different positions were commanded to the robot and 100 iterations were carried out for each position to generate the position data. To calculate the positioning repeatability the following calculations were carried out.

$$l_i = \sqrt{(x_i - \bar{x})^2} \qquad (8)$$

$$\bar{l} = \frac{1}{n}\sum_{i=1}^{n} l_i \qquad (9)$$

Where 'x_i' is the measured value in i^th iteration, '$\bar{x}$' is the mean position of all iterations, 'n' is the number of iterations, and '$\bar{l}$' is then the mean positional repeatability according to ANSI/RIA R15.05 standard (38). Moreover, the standard deviation 'S' is then calculated as:

$$S = \sqrt{\sum_{i=1}^{n} \frac{(l_i - \bar{l})^2}{n-1}} \qquad (10)$$

Lastly, the positional repeatability, 'Rp', is defined and calculated as

$$Rp = \bar{l} + 3S \qquad (11)$$

Table ~~5~~ 7 shows the Standard Deviation and the Positional Repeatability for each position. The worst value of the repeatability achieved in this experimentation is claimed as the repeatability of the developed robot prototype. Comparison with commercially available lightweight robots establishes that this value of repeatability is acceptable for robotic manipulators. The ~~worst case~~worst-case value of repeatability, 0.0503 mm, of our developed robotic manipulator is equivalent to commercially available lightweight robot Techman Robot TM5 (39).

Table ~~5~~7: Standard Deviation and Repeatability of 5-DOF robot measured and calculated as suggested in ISO 9283:1998 for five different positions. All values are in mm.

Robot	Position 1		Position 2		Position 3		Position 4		Position 5	
	S	Rp	S	Rp	S	Rp	S	Rp	S	Rp
5-DOF	0.0018	0.0080	0.0039	0.0163	0.0062	0.0260	0.0107	0.0503	0.0030	0.0138

Overall, the developed simulation tool demonstrates its potential for robot design and ~~optimization~~development, particularly in the selection of integrated drive joint subcomponents. Using the proposed scheme, the authors along with their research team have also developed a 6-DOF robot manipulator, with ~~having a reach of~~ 1 m reach and 4 Kg payload capacity~~ of 4 kg~~, shown in Fig 27,~~.~~ ~~However, it's~~ is ~~still~~ in the testing phase. Fig 27 shows the configuration and hardware of the~~is~~ new~~ly developed~~ manipulator.

Fig 27: Newly developed 6 DOF Robot's FBD (Left) and its hardware (Right).

Section V

Conclusion and Future Work~~s~~

~~In conclusion, this research emphasizes the advantages of adopting a component-driven approach in the design and fabrication of lightweight robotic manipulators. By developing models of commercially available components and integrating them into the design process, the gap between simulation and physical systems can be significantly reduced, leading to elimination or reduction of various steps involved in the robotic manipulator development process.~~ This research underscores the benefits of a component-driven approach for designing and building lightweight robotic manipulators. By leveraging models of commercially available components within the design process, the gap between simulations and real-world systems narrows significantly. This can lead to eliminating or streamlining various steps in the development process, resulting in faster and more efficient creation of lightweight robotic manipulators.

The presented framework demonstrates the effectiveness of this approach through the modeling and analysis ~~of integrated drive joints in~~of a physical ~~virtual~~ prototype of a 5-DOF lightweight robotic manipulator. Notably, the experimental evaluation of the developed robotic system yielded impressive results. The positional repeatability, assessed in accordance with ISO 9283:1998, demonstrated a repeatability of 0.05 mm, a performance level comparable to the Techman Robot TM5, a commercially available lightweight robot.

~~Subsequent hardware experimentation, where the same integrated drive joints and robot structure were manufactured and driven on predetermined trajectories, revealed that while position and velocity trajectories closely matched the simulated model, a discernible difference in torque requirements was observed. This disparity can be attributed to unmodeled dynamics, such as friction arising from strain wave gears and misalignments in the assembly process. Nevertheless, the magnitude of the torque difference remained small compared to the nominal torque capability of the joints~~ Following hardware experiments, where the same integrated drive joints and robot structure were manufactured and tested along predetermined trajectories, results showed close alignment between the simulated model and the actual position and velocity readings. However, a noticeable difference was observed in the required torque compared to the simulation. This discrepancy can be attributed to factors not included in the model, such as friction from the strain wave gears and potential misalignments introduced during assembly. Encouragingly, the magnitude of this torque difference remained minor compared to the joints' nominal torque capacity.~~.~~

~~The proposed simulation tool and its framework hold significant potential in revolutionizing the robot manufacturing industry. By enabling developers working in educational~~

research institutes, robotics startups, and other similar positions, to simulate robotic manipulators using models of actual subcomponents, it offers substantial time, effort, and investment savings in today's fast-paced and resource-intensive environment Our study presents a simulation tool and framework with the potential to significantly transform the robot manufacturing industry. By allowing developers in educational research, robotics startups, and related fields to simulate manipulators using real-world component models, it offers substantial savings in time, effort, and investment. This is particularly valuable in today's fast-paced and resource-constrained environment..

Future research endeavors will focus on further refining the simulation tool, expanding the library of simulated models to incorporate components from various manufacturers, and addressing the challenges posed by unmodeled dynamics. This ongoing development will continue to enhance the accuracy, reliability, and practicality of the proposed framework, further solidifying its potential as a game-changer in the field of robot manufacturing Future work will focus on several key areas: refining the simulation tool for even greater accuracy, expanding the library of simulated models to encompass a wider range of components from diverse manufacturers, and tackling the complexities of unmodeled dynamics. By continuously enhancing the framework's accuracy, reliability, and user-friendliness, we aim to solidify its position as a transformative tool within the robot manufacturing industry.

In conclusion, the component-driven approach presented in this research has demonstrated its effectiveness in facilitating the rapid development of lightweight robotic manipulators. By leveraging commercially available components and optimizing their integration through simulation and hardware experimentation, this framework offers valuable insights and practical solutions for the advancement of robotic systems.

Acknowledgements

This research is supported by the Higher Education Commission (HEC) Pakistan under the project 'The establishment of National Center for Robotics and Automation (NCRA). We would also like to thank Dr. Ali Raza, Principal Investigator (PI) of Human Centered Robotics Lab UET Lahore for his unwavering support and guidance.

References

1. Akundi A, Euresti D, Luna S, Ankobiah W, Lopes A, Edinbarough I. State of Industry 5.0—Analysis and identification of current research trends. Applied System Innovation. 2022;5(1):27.
2. Hirzinger G, Albu-Schaffer A, Hahnle M, Schaefer I, Sporer N, editors. On a new generation of torque controlled light-weight robots. Proceedings 2001 ICRA IEEE International Conference on Robotics and Automation (Cat No 01CH37164); 2001: IEEE.
3. Popić S, Miloradović B. Light weight robot arms-an overview. INFOTEH-JAHORINA. 2015;14.
4. Balding S, Gning A, Cheng Y, Iqbal J. Information Rich Voxel Grid for Use in Heterogeneous Multi-Agent Robotics. Applied Sciences. 2023;13(8):5065.

5. Bilal M, Rizwan M, Maqbool HF, Ahsan M, Raza A. Design optimization of powered ankle prosthesis to reduce peak power requirement. Science Progress. 2022;105(3):00368504221117895.
6. Naeem A, Rizwan M, Maqbool HF, Ahsan M, Raza A, Abouhossein A, et al. Virtual constraint control of Knee-Ankle prosthesis using an improved estimate of the thigh phase-variable. Biomedical Signal Processing and Control. 2022;73:103366.
7. Calvo R, Gil P. Evaluation of collaborative robot sustainable integration in manufacturing assembly by using process time savings. Materials. 2022;15(2):611.
8. Gheorghe G, editor Concepts and mechatronics and cyber-mixmechatronics constructions, integrated in COBOT type technology platform for intelligent industry (4.0). Proceedings of the International Conference of Mechatronics and Cyber-MixMechatronics–2019 3; 2020: Springer.
9. Timms MJ. Letting artificial intelligence in education out of the box: educational cobots and smart classrooms. International Journal of Artificial Intelligence in Education. 2016;26:701-12.
10. Djuric AM, Urbanic R, Rickli J. A framework for collaborative robot (CoBot) integration in advanced manufacturing systems. SAE International Journal of Materials and Manufacturing. 2016;9(2):457-64.
11. Chung JC, Hwang T-S, Wu C-T, Jiang Y, Wang J-Y, Bai Y, et al. Framework for integrated mechanical design automation. Computer-aided design. 2000;32(5-6):355-65.
12. SANCHEZ S. Career Karma2022. Available from: https://careerkarma.com/blog/how-to-build-your-own-robot/#:~:text=How%20Long%20Does%20It%20Take,mechanical%20and%20electrical%20assembly%20expertise.
13. Ruishu Z, Chang Z, Weigang Z, editors. The status and development of industrial robots. IOP Conference Series: Materials Science and Engineering; 2018: IOP Publishing.
14. BRACJUNOVÁ VL. Robots take the robot out of the human: Soitron Group; 2019 [Available from: https://www.soitron.com/robots-take-the-robot-out-of-the-human/#:~:text=Creating%20a%20new%20robot%20from,part%20of%20the%20development%20process.
15. Hirose S, Arikawa K. Coupled and decoupled actuation of robotic mechanisms. Advanced Robotics. 2001;15(2):125-38.
16. Tarkian M, O¨ lvander J, Feng X, Petterson M, editors. Design automation of modular industrial robots. International Design Engineering Technical Conferences and Computers and Information in Engineering Conference; 2009.
17. Covell DM. Top-Down & Bottom-Up Approaches to Robot Design: West Virginia University; 2022.
18. Zimmermann M, Königs S, Niemeyer C, Fender J, Zeherbauer C, Vitale R, et al. On the design of large systems subject to uncertainty. Journal of Engineering Design. 2017;28(4):233-54.
19. Sathuluri A, Sureshbabu AV, Frank J, Amm M, Zimmermann M. Computational Systems Design of Low-Cost Lightweight Robots. Robotics. 2023;12(4):91.
20. Walden DD. Systems engineering handbook: A guide for system life cycle processes and activities. (No Title). 2015.
21. Albu-Schäffer A, Haddadin S, Ott C, Stemmer A, Wimböck T, Hirzinger G. The DLR lightweight robot: design and control concepts for robots in human environments. Industrial Robot: an international journal. 2007;34(5):376-85.
22. Bolignari M, Fontana M. Design and experimental characterization of a high performance hydrostatic transmission for robot actuation. Meccanica. 2020;55(5):1169-79.
23. Mo S, Li X, Yang Z-n, Zhou C-p, Gao H-j, Cen G-j, et al. Integrated design of brushless motor drive and control system for robot joints. Journal of Central South University. 2021;28(12):3818-28.

24. Bischoff R, Kurth J, Schreiber G, Koeppe R, Albu-Schäffer A, Beyer A, et al., editors. The KUKA-DLR Lightweight Robot arm-a new reference platform for robotics research and manufacturing. ISR 2010 (41st international symposium on robotics) and ROBOTIK 2010 (6th German conference on robotics); 2010: VDE.
25. Mohanraj D, Aruldavid R, Verma R, Sathyasekar K, Barnawi AB, Chokkalingam B, et al. A Review of BLDC Motor: State of Art, Advanced Control Techniques, and Applications. IEEE Access. 2022.
26. Routh B, editor Design aspects of harmonic drive gear and performance improvement of its by problems identification: A review. AIP conference proceedings; 2018: AIP Publishing LLC.
27. Padilla-Garcia EA, Rodriguez-Angeles A, Resendiz JR, Cruz-Villar CA. Concurrent optimization for selection and control of AC servomotors on the powertrain of industrial robots. IEEE Access. 2018;6:27923-38.
28. Padilla-García EA, Cervantes-Culebro H, Rodriguez-Angeles A, Cruz-Villar CA. Selection/control concurrent optimization of BLDC motors for industrial robots. Plos one. 2023;18(8):e0289717.
29. Cusimano G, Casolo F. An almost comprehensive approach for the choice of motor and transmission in mechatronic applications: Torque peak of the motor. Machines. 2021;9(8):159.
30. Albu‐Schäffer A, Haddadin S, Ott C, Stemmer A, Wimböck T, Hirzinger G. The DLR lightweight robot: design and control concepts for robots in human environments. Industrial Robot: an international journal. 2007.
31. Machado TDN, Sousa M, Monteiro S, Bicho E. CoopDynSim: a 3D robotics simulator. 2012.
32. Baizid K, Ćuković S, Iqbal J, Yousnadj A, Chellali R, Meddahi A, et al. IRoSim: Industrial Robotics Simulation Design Planning and Optimization platform based on CAD and knowledgeware technologies. Robotics and Computer-Integrated Manufacturing. 2016;42:121-34.
33. Bose BK. Modern power electronics and AC drives: Prentice hall Upper Saddle River, NJ; 2002.
34. Gutiérrez S, Zotovic R, Navarro M, Meseguer M. Design and manufacturing of a prototype of a lightweight robot arm. Procedia Manufacturing. 2017;13:283-90.
35. ISO. ISO/TS 15066:2016(en)

Robots and robotic devices — Collaborative robots 2016 [Available from: https://www.iso.org/obp/ui/#iso:std:iso:ts:15066:ed-1:v1:en.
36. San-Miguel A, Puig V, Alenyà G. Disturbance observer-based LPV feedback control of a N-DoF robotic manipulator including compliance through gain shifting. Control Engineering Practice. 2021;115:104887.
37. Standardization TIOf. ISO 9283:1998(en) Manipulating industrial robots — Performance criteria and related test methods. 1998.
38. Institute ANS. American National Standard for Industrial Robots and Robot Systems - Point-to-Point and Static Performance Characteristics – Evaluation. ANSI/RIA R1505-1-1990 (R1999)1999.
39. TECHMANROBOTINC. TM5 - 900 2019 [Available from: https://www.tm-robot.com/en/tm5-900/.

RESPONSE TO REVIEWERS

REVIEWER # 3

Comment # 1: English should be revised in order to better understand the manuscript.

Response: We have improved the manuscript grammar for improved coherence and readability, using a professional digital writing assistant tool.

Comment # 2: The state of the art on design methodologies must be reviewed and increased. Moreover, the manuscript's contribution needs to be clarified; there is no discussion of how this work differs from the state of the art. For example:
a) Padilla-García, E. A., Cervantes-Culebro, H., Rodriguez-Angeles, A., & Cruz-Villar, C. A. (2023). Selection/control concurrent optimization of BLDC motors for industrial robots. Plos one, 18(8), e0289717.
b) Cencen, A., Verlinden, J. C., & Geraedts, J. M. P. (2018). Design methodology to improve human-robot coproduction in small-and medium-sized enterprises. IEEE/ASME Transactions on Mechatronics, 23(3), 1092-1102.
c) Ronzoni, M., Accorsi, R., Botti, L., & Manzini, R. (2021). A support-design framework for Cooperative Robots systems in labor-intensive manufacturing processes. Journal of Manufacturing Systems, 61, 646-657.
d) Agarwal, P., Neptune, R. R., & Deshpande, A. D. (2016). A simulation framework for virtual prototyping of robotic exoskeletons. Journal of biomechanical engineering, 138(6), 061004.
e) Havard, V., Jeanne, B., Lacomblez, M., & Baudry, D. (2019). Digital twin and virtual reality: a co-simulation environment for design and assessment of industrial workstations. Production & Manufacturing Research, 7(1), 472-489.
f) E. A. Padilla-Garcia, A. Rodriguez-Angeles, J. R. ReséNdiz and C. A. Cruz-Villar, "Concurrent Optimization for Selection and Control of AC Servomotors on the Powertrain of Industrial Robots," in IEEE Access, vol. 6, pp. 27923-27938, 2018, doi: 10.1109/ACCESS.2018.2840537
g) Cusimano G, Casolo F. An almost comprehensive approach for the choice of motor and transmission in mechatronic applications: Torque peak of the motor. Machines. 2021;9(8):159.

Response: We extend the literature review by adding synopses of the recommended research articles. A summary of changes made after reviewing the papers is presented here as well.

Our research focuses on designing a lightweight robotic manipulator from scratch, unlike studies (a) and (f) that address selecting off-the-shelf BLDC and AC servo motors. These prior works treated the selection as a multi-variable optimization problem, minimizing energy consumption, tracking error, and motor weight.

We propose a more holistic design methodology, where selecting a frameless BLDC motor is just one step. We use the standard AC7 block in Simulink/MATLAB to model the motor with manufacturer-provided data. Similarly, SolidWorks, with data sheet parameters, models the strain wave gears. These parameters are used in both the CAD model and the actuator model, which incorporates the motor, gears, and other components.

Custom-designed links complete the robot. Due to the diverse parts, our BLDC motor control analysis is less detailed. Our focus is establishing a framework for a lightweight robot using off-the-shelf components like frameless BLDC motors, strain wave gears, motor controllers, and custom links. Studies (b), (c), and (e) cover collaborative robot work cell design. Study (d) presents a virtual prototyping framework for rehabilitation exoskeletons. These are somewhat peripheral to our work.

Finally, study (g) describes a method for testing individual drive-transmission pairs, not considering them as a multi-DOF coupled system. While this method can analyze individual pairs, it lacks analysis for a complete system with interconnected joints.

Comment # 3: More specifications must be provided including torque saturations, peak and steady-state current limits, and controller gains for stability.

Response: For rapid component model configuration and simulation, we relied solely on the specifications provided in the component data sheets. This approach streamlines the process. We employed also the standard AC7 block within Simulink/MATLAB to model and drive the frameless BLDC motor. The hardware motor controller was then fine-tuned using the manufacturer's recommended method.

Comment # 4: In order to ensure clarity and accuracy, it would be greatly appreciated if a flowchart of the proposed methodology could be provided, highlighting the selected steps in yellow. This would aid in understanding the proposed methodology and facilitate the necessary review process.

Response: The requested flowchart is already provided in the manuscript. Figure 1 illustrates the traditional design process for a mechatronic system, such as a robotic manipulator. The following explanation identifies the steps (highlighted with red bounding boxes) that our proposed methodology eliminates. Figure 3 then depicts the workflow of our proposed design and development cycle, starting from robot requirements and culminating in the completed robot hardware. For further clarity, the development of the subcomponent library is now detailed in the Methodology section, subsection 'Simulation Model Flow' of the revised manuscript.

Comment # 5: At present, the stress analysis is carried out solely from a static point of view, with the maximum load applied to the final effector being the only consideration. However, the dynamic response of the system is influenced by the controller's gain tuning, and as such, the magnitude of values attained may surpass those observed in the static analysis.

Response: Static stress analysis with a high factor of safety (2.4) guarantees that the robot's structure can withstand dynamic stresses encountered during operation. In other words, this analysis validates the structural integrity of the robot links, ensuring they can endure the worst-case scenario without failure. This approach minimizes the risk of structural failure during robot operation.

Comment # 6: Can you please clarify which method was used to find the optimal solution for the built robot? Also, could you explain why some of the methods available in the Matlab

libraries were not utilized? It would be helpful if the wording could be more precise since the introduction states that the optimization stage was eliminated.

Response: We achieved an optimal solution through an iterative design refinement process. This involved continuously improving the robot design until it achieved satisfactory performance, that is correlation coefficient greater than 0.95, on trajectory following test cases for a typical lightweight robot, as shown in Figure 12 and 14. While explicit optimization was not a primary focus in this stage of our research, we recognize its potential significance and intend to explore it further in future iterations of the development process. To validate functional performance, our criterion requires the physical prototype to achieve repeatability on the order of magnitude of the commercially available lightweight robot (+/- 0.05 for the Techman Robot TM5 robot, while ours is +/- 0.0503)

It is unclear from the referee's comment what *additional* MATLAB libraries/tools could have been potentially useful for this study. We have utilized relevant MATLAB built-in models and CAD Modeling software. The objective was to create models capable of simulating performance that closely aligns with experimental outcomes. By assembling these models into a virtual prototype of the desired robotic manipulator, we aimed to demonstrate that the simulated performance would be comparable to the physical counterpart. As shown in the results section, the performance of the physically developed robot indeed matched that of the simulated model, validating our approach.

Our focus was on establishing the efficacy of using virtual prototypes built from models of commercially available components, thereby potentially bypassing costly and time-consuming physical design iterations. Additionally, the creation and maintenance of a library containing such components could further streamline the initial design phase.

Comment # 7: The proposed trajectories exhibit non-differentiability, thereby posing potential safety hazards for the cobot by introducing acceleration inconsistencies. This issue necessitates immediate attention and resolution to ensure the cobot's safe and optimal performance.

Response: The figure (Figure 1) below shows the magnified view of one of the velocity profiles. This establishes that the trajectories are continuous and *perfectly* differentiable. Perhaps a lengthy time axis spanning 17 seconds (Section III), squeezing the trajectory curve, may have given an illusion that the trajectories are discontinuous or non-differentiable, which is obviously not the case.

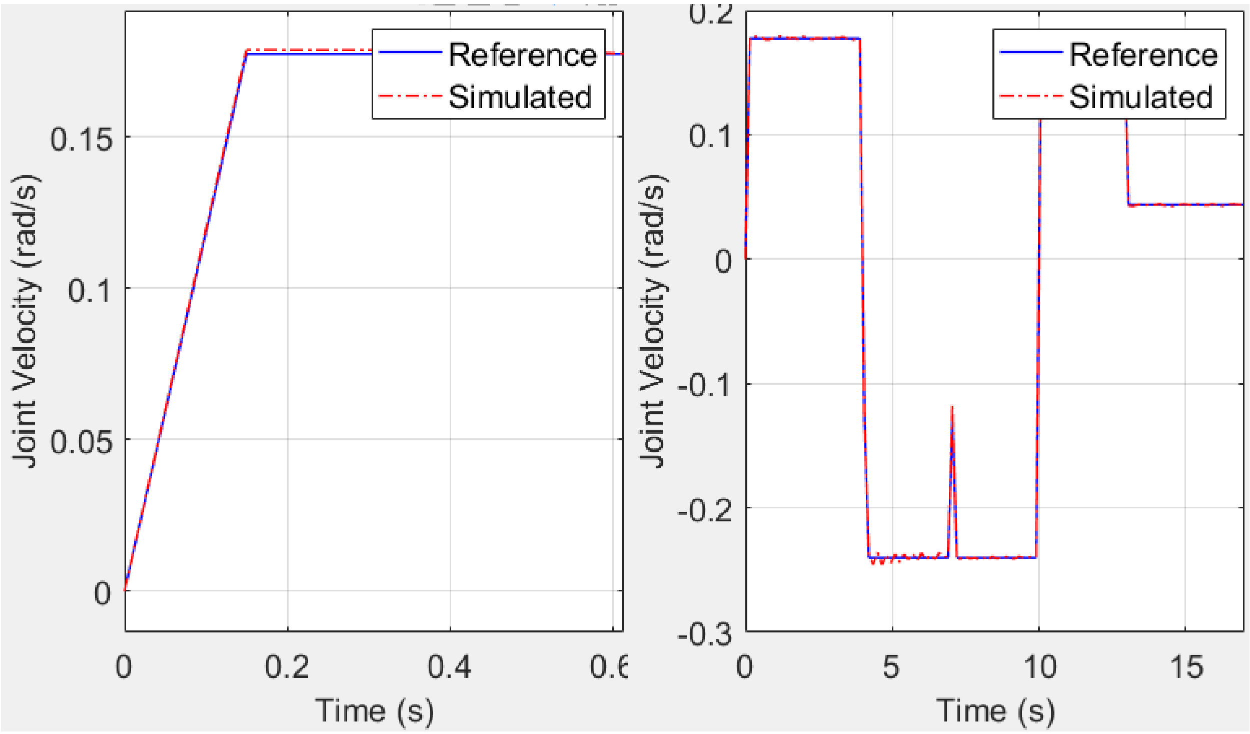

Figure 1 : (LEFT) Magnified view of the Velocity Trajectory signifying that a Trapezoidal Velocity Profile was used to drive the robot. (RIGHT) The trajectory as reported in the manuscript.

Comment # 8: The specifics of the simulation should be included, e.g., the tuning of the gains of each degree of freedom, the sampling time, and the solver used to solve the differential equations.

Response: The frameless BLDC motors were modeled and simulated using the AC7 block from the Simulink/MATLAB Specialized Power Systems Library. This block facilitates automatic tuning of the implemented PI controllers for position, velocity, and current control. The simulation was run with a fixed step size of 0.1 ms. The specific solver selection was left to Simulink, which by default utilizes the Backward Euler solver.

Comment # 9: A section should include all Solidworks parts and Matlab/Simscape files of the obtained design.

Response: All the relevant files will become part of the public repository as per the journal guidelines.

Comment # 10: It is recommended to enhance the quality of all images. Additionally, capture images where only the robot is visible.

Response: This comment is related to Fig 19. Quality/Resolution of all images is as per the journal specifications. Moreover, the images were taken in an R&D lab where the equipment visible in the image is part of the whole robotic system. Therefore, we believe it makes more sense to retain these images without modifications.

Comment # 11: Each of the figures should be described and discussed in detail in the text.

Response: The revised manuscript adequately addresses this concern. The explanation of the figures now appears after Table 4, closer to the graphs.

Comment # 12: Improve image quality. What is the maximum mass that the end effector can move? (Fig.27)

Response: The revised manuscript provides this information for both manipulators in appropriate sections.

Comment # 13: Could you kindly elaborate on what your goals are so that I can provide the most relevant insights? What are the criteria considered to design this robot?

Response: Introduction and other sections have been revised to address this comment. The criterion was reproduced in comment#6.

Comment # 14: Why use a correlation measure between data and not an error measure between the simulation and the physical prototype?

Response: Three standard metrics of trajectory error are:

1- Quantified Error Measure. It can be Mean Squared Error (MSE), Mean Absolute Error (MAE), Root Mean Squared Error (RMSE) etc.

2- Correlation Coefficient. This coefficient quantifies the linear relation of the reference trajectory with the measured trajectory.

3- Visual Comparison. Graphs of reference trajectories overlaid on the measured trajectories are used to represent the trajectory tracking error.

Originally, we used the Correlation Coefficient and the Visual Comparison for representation of the trajectory tracking error. However, to incorporate valuable reviewers' suggestions, we have added the RMSE measure as well. We have calculated, stated, and discussed the RMSE for the position, velocity, and torque trajectories in the Hardware Experimentation section in the revised manuscript.

Comment # 15: Clarify what they mean by satisfactory performance. It helps refer to equations or constraints of the system.

Response: For optimal design we quantitatively explain the criterion of satisfactory performance in the last paragraph of Section II of the revised manuscript, as well as in the **response** to comment # 6. For the functional performance validation, our criterion requires that the physical prototype attains repeatability of the order of magnitude (ours is 0.05) same as that of the commercially available lightweight robot (0.05 for Techman Robot TM5).

Comment # 16: A section should include all Solidworks parts and Matlab/Simscape files of the obtained design.

Response: Already addressed in response to comment # 9.

Comment # 17: Web pages should be omitted as references, instead sources from indexed journals should be sought.

Response: The information extracted from the weblinks is not available in any published or indexed journals to the best of our knowledge. Moreover, there are no restrictions on referencing a website in PLOS ONE article.

Comment # 18: It needs to be understood what initial design methodology needs to be improved. In addition, it is necessary to add where the changes are made concerning the proposed method.

Response: The manuscript Section III subsection Simulation Model Flow and initial paragraphs of Section IV Simulation Experimentation details the design methodology. To highlight it further, we have updated parts of the Introduction and Section II as well.

Comment # 19: Review the rest of the observations in the attached file.

Response: We have reviewed the all the observations in the attached file and have tried our best to address all of them.

REVIEWER # 4

Comment # 1: The authors should add more quantitative data/analysis. The paper presents some quantitative results from simulations and experiments, but more data could strengthen the conclusions.
For example, providing specific statistics on the time/cost savings achieved using their method compared to traditional approaches, of course, based on explicit cites about the time/cost of traditional approaches.

Response: While we couldn't locate a directly comparable robotic arm case study in the literature regarding design cycle time cost savings, we've incorporated valuable insights from online resources. These resources provide general time allocation percentages for each design stage, further explained in the revised manuscript's Introduction section.

Our research offers a distinct contribution by presenting a quantitative analysis of time and human resource savings based on the robotic arm R&D conducted within our lab. This data, presented in Table 1 of the revised manuscript showcasing internal R&D man-hour savings, highlights the practical benefits of our proposed methodology.

Comment # 2: The authors use simple PI control but, the justification for this implementation is missing. It is common to use this type of controller for BLDC motors, but it is imperative to justify, at least with standard or state-of-the-art information about the advantages of PI over PD or PID controller for BLDC motors. On the other hand, the justification of using a PI controller can be done by results in the simulation of the system and get the justification that way.

Response: Our choice to simulate only a PI controller aligns with the practical reality that most commercially available BLDC motor controllers use PI control, as explained in the 'Frameless BLDC Motor Model' subsection of the Methodology section. Additionally, the same section discusses the advantages of PI control over PD or PID control for BLDC motors.

Comment # 3: It is imperative that the authors add data analysis between the simulation and experimental results regarding the trajectory tracking errors in terms of the error. Additionally, add values of the tracking error in joint space between the reference and the measured values of the robot.

Response: The revised manuscript now includes a section on Hardware Experimentation that analyzes the trajectory tracking error in joint space between the reference and measured trajectories.

REVIEWER # 5

Comment # 1: All equations should be numbered and referenced when needed.

Response: We have numbered all the equations.

Comment # 2: The configured joints of the manipulator were configured with a damping constant of 2 Nm/deg/s, the usual SI damping units are Ns/m.

Response: Ns/m is the SI unit for Linear damping; however, Nm/rad/s is the SI unit for the rotational damping coefficient. Nm/deg/s has been changed to Nm/rad/s which is the SI unit for rotational damping coefficient.

Comment # 3: The iterated simulation using different integrated drive joints, does not yield an optimal joint as it's mentioned.

Response: Our iterative design process begins by selecting commercially available, low-powered BLDC motors for the joints. If a joint fails to meet the desired trajectory due to motor torque or power limitations, we implement a power upgrade. This involves replacing the motor with the next higher-power motor that is commercially available. We continue this iterative process until the design achieves the required performance. For a more detailed response, please refer to our response to comment#6.

Comment # 4: Fig 8 does not show the Brushless DC Motor Drive (AC7) block and the connections used.

Response: Figure 8 shows only the Simulink SimScape Multibody model of the robot. Figure 11 shows the connection of the robot with the motors. Unfortunately, it is not possible to spawn the built-in macro and reveal the requested blocks, again, due to the limitations of the simulation tool.

Comment # 5: It is mentioned in the Abstract that the design cycle time is reduced by approximately 40% but no substantial evidence of this is explained during the results. Modifying the design approach does not inherently reduce the design cycle time since time saved in some areas may increase development time in other areas.

Response: The evidence is provided in Table 1 of the revised manuscript.

While it's true that changes can introduce unforeseen complexities, a well-chosen design approach can significantly reduce overall design cycle time, as demonstrated in this study. The history of the Mechatronics industry is a testament to this concept. Continuous exploration and adoption of new design methodologies have been a key driver of efficiency and innovation throughout the field's evolution.
