# Supplementary material for: Experimental development of lightweight manipulators with improved design cycle time that leverages off-the-shelf robotic arm components
Source: PLoS One. 2024 Jul 18;19(7):e0305379. doi: 10.1371/journal.pone.0305379 (PMC11257280; doi:10.1371/journal.pone.0305379)
Supplement: Attachment — Submitted filename: Response to Reviewers.docx [file pone.0305379.s001.docx]

Dear Editor,

We appreciate insightful comments and suggestions on our submitted manuscript. We take this opportunity to address the comments/concerns raised by you or by the reviewers in the revised version of our manuscript. Point-by-point responses are given below.

**Comments of the Academic Editor and Responses**

Comment # 1. When submitting your revision, we need you to address these additional requirements.

Please ensure that your manuscript meets PLOS ONE's style requirements, including those for file naming. The PLOS ONE style templates can be found at 
<https://journals.plos.org/plosone/s/file?id=wjVg/PLOSOne_formatting_sample_main_body.pdf> and 
<https://journals.plos.org/plosone/s/file?id=ba62/PLOSOne_formatting_sample_title_authors_affiliations.pdf>.

**Response: We have updated our manuscript as per the formatting guidelines provided and hope that now the manuscript meets the formatting requirements of the PLOS ONE journal.**

Comment # 2. Please note that PLOS ONE has specific guidelines on code sharing for submissions in which author-generated code underpins the findings in the manuscript. In these cases, all author-generated code must be made available without restrictions upon publication of the work. Please review our guidelines at <https://journals.plos.org/plosone/s/materials-and-software-sharing#loc-sharing-code> and ensure that your code is shared in a way that follows best practice and facilitates reproducibility and reuse.

**Response: We fully understand the guidelines on code sharing and the code files shall be made available when required.**

Comment # 3. We note that Figure(s) 2, 5, 6, 7, 8, 9, 10, 18 and 26 in your submission contain copyrighted images. All PLOS content is published under the Creative Commons Attribution License (CC BY 4.0), which means that the manuscript, images, and Supporting Information files will be freely available online, and any third party is permitted to access, download, copy, distribute, and use these materials in any way, even commercially, with proper attribution. For more information, see our copyright guidelines: <http://journals.plos.org/plosone/s/licenses-and-copyright>.
 
We require you to either (1) present written permission from the copyright holder to publish these figures specifically under the CC BY 4.0 license, or (2) remove the figures from your submission:

a. You may seek permission from the original copyright holder of Figure(s) 2, 5, 6, 7, 8, 9, 10, 18 and 26 to publish the content specifically under the CC BY 4.0 license.

We recommend that you contact the original copyright holder with the Content Permission Form (<http://journals.plos.org/plosone/s/file?id=7c09/content-permission-form.pdf>) and the following text:
“I request permission for the open-access journal PLOS ONE to publish XXX under the Creative Commons Attribution License (CCAL) CC BY 4.0 (<http://creativecommons.org/licenses/by/4.0/>). Please be aware that this license allows unrestricted use and distribution, even commercially, by third parties. Please reply and provide explicit written permission to publish XXX under a CC BY license and complete the attached form.”
 
Please upload the completed Content Permission Form or other proof of granted permissions as an ""Other"" file with your submission.

In the figure caption of the copyrighted figure, please include the following text: “Reprinted from [ref] under a CC BY license, with permission from [name of publisher], original copyright [original copyright year].”
 
b. If you are unable to obtain permission from the original copyright holder to publish these figures under the CC BY 4.0 license or if the copyright holder’s requirements are incompatible with the CC BY 4.0 license, please either i) remove the figure or ii) supply a replacement figure that complies with the CC BY 4.0 license. Please check copyright information on all replacement figures and update the figure caption with source information. If applicable, please specify in the figure caption text when a figure is similar but not identical to the original image and is therefore for illustrative purposes only.

**Response: We have obtained the permission of the copyright holder, who is the Principal Investigator (PI) of the Human-Centered Robotics Lab, under which the whole research is being carried out. Moreover, Muhammad Ahsan (2^nd^ author) is also the Co-PI of the same lab. The completed Content Permission Form is attached in the relevant section. Furthermore, it is stated that figures created using MATLAB software does not contain any MATLAB code files supplied from MATLAB’s system.**

Comment # 4. Please amend the manuscript submission data (via Edit Submission) to include author Muhammad Ahsan.

**Response: We have updated the author data.**

Comment # 5. Please amend your authorship list in your manuscript file to include author Ahsan Ali.

**Response: There was a typing error in the name. The name ‘Ahsan Ali’ is in fact ‘Muhammad Ahsan’. We have addressed this error.**

**Comments of Reviewer # 1 and Responses**

Comment # 1. The authors should delve into existing literature and cite papers that address the reduction of the conventional design cycle in robotics.

**Response: We have reinforced the literature review at the start of Section II by including multiple latest references related to the optimization of design cycle in robotics. The newly added discussion may be found at the start of Section II.**

Comment # 2. Although a PI controller was utilized for the motor, the authors did not explore alternative controllers like PID or PD. It remains unclear why other controller types were not considered. Further investigation into the choice of the PI controller and the omission of PID or PD controllers could provide valuable insights into the motor control strategy adopted in this study. The authors can provide justification through simulation results as well.

**Response: In the second paragraph of the subsection titled "Frameless BLDC Motor Model," we explicitly mention that commercial motor drives may utilize PI, PD, or PID controllers for motor control. However, in the subsequent paragraph (para 3 of the same subsection), we clarify that the specific hardware motor controllers employed in our study exclusively incorporate a PI control loop. Consequently, our decision to simulate only the PI controller is reflective of the actual control mechanism embedded in the hardware utilized for this research.**

**Moreover, it is noteworthy that commercial BLDC motor controllers commonly employ PI controllers due to their effectiveness in various applications. This aligns with the prevalent industry practice, and it was this real-world consideration that guided our simulation approach.**

**To further elucidate this point, we have refined the discussion in the relevant section of the manuscript to ensure clarity on our rationale for simulating the PI controller exclusively.**

Comment # 3. The authors presented simulation and experimental results, yet they should further validate the design approach's feasibility by exploring additional scenarios, particularly under diverse disturbance conditions. Demonstrating the system's performance in the face of various disturbances would enhance the comprehensiveness and robustness of their findings.

**Response:** **For industrial robotic manipulators, primary sources of disturbance include environmental interaction, unmodeled dynamics, and gravitational effects. However, it's important to note that the scope of our research primarily focuses on assessing the impact of commercially available components on the overall robotic manipulator design.**

**Interaction with the environment involves torque control schemes implemented at the system level, and its consideration is often application dependent. As such, it falls beyond the specific objectives of our study. Regarding unmodeled dynamics, our experimentation explicitly addresses the system-level robustness against such dynamics, as mentioned in the manuscript. Lastly, the SimScape dynamic simulation accounts for gravitational effects in our model. The demonstrated positional accuracy in both simulation and hardware experimentation indicates that gravitational effects are appropriately managed throughout the trajectory, leveraging additional torque well within the actuator's specified limits.**

**To further exhibit the strength of our simulated models and in alignment with your suggestion, we have conducted additional simulations introducing white Gaussian noise at the individual motor level to specifically evaluate the efficacy of the individual PI controller in rejecting such disturbances. We have incorporated corresponding discussions and graphical data, Fig 18, in the relevant section of the manuscript, para 3, 9 and 10 of Section III Simulation Experimentation. These additions aim to provide a more comprehensive understanding of the disturbance rejection capabilities at the individual motor level.**

Comment # 4. The authors didn’t follow the formatting guidelines of the journal available on the Plos One website.

**Response: We have updated the formatting as per the guidelines provided by the academic editor.**

Comment # 5. The references are not formatted according to the “International Committee of Medical Journal Editors (ICMJE)”. The reference should include all the information, such as the month of publication, and place in case of a conference.

**Response: We have changed the reference style to that of ICMJE.**

Comment # 6. Please report the values in SI units in Table 1.

**Response: We have converted all the values to SI units in Table 1.**

We trust that these updates contribute significantly to addressing your concerns and further enriching the content of our manuscript. We look forward to any additional guidance or feedback you may have.

Thank you for your time and consideration.
