# Supplementary material for: Experimental development of lightweight manipulators with improved design cycle time that leverages off-the-shelf robotic arm components
Source: PLoS One. 2024 Jul 18;19(7):e0305379. doi: 10.1371/journal.pone.0305379 (PMC11257280; doi:10.1371/journal.pone.0305379)
Supplement: Attachment — Submitted filename: Response_to_Reviewers.docx [file pone.0305379.s003.docx]

**RESPONSE TO REVIEWERS**

**REVIEWER # 3**

**Comment # 1:** English should be revised in order to better understand the manuscript.

**Response:** We have improved the manuscript grammar for improved coherence and readability, using a professional digital writing assistant tool.

**Comment # 2:** The state of the art on design methodologies must be reviewed and increased. Moreover, the manuscript's contribution needs to be clarified; there is no discussion of how this work differs from the state of the art. For example:
a) Padilla-García, E. A., Cervantes-Culebro, H., Rodriguez-Angeles, A., & Cruz-Villar, C. A. (2023). Selection/control concurrent optimization of BLDC motors for industrial robots. Plos one, 18(8), e0289717.
b) Cencen, A., Verlinden, J. C., & Geraedts, J. M. P. (2018). Design methodology to improve human-robot coproduction in small-and medium-sized enterprises. IEEE/ASME Transactions on Mechatronics, 23(3), 1092-1102.
c) Ronzoni, M., Accorsi, R., Botti, L., & Manzini, R. (2021). A support-design framework for Cooperative Robots systems in labor-intensive manufacturing processes. Journal of Manufacturing Systems, 61, 646-657.
d) Agarwal, P., Neptune, R. R., & Deshpande, A. D. (2016). A simulation framework for virtual prototyping of robotic exoskeletons. Journal of biomechanical engineering, 138(6), 061004.
e) Havard, V., Jeanne, B., Lacomblez, M., & Baudry, D. (2019). Digital twin and virtual reality: a co-simulation environment for design and assessment of industrial workstations. Production & Manufacturing Research, 7(1), 472-489.
f) E. A. Padilla-Garcia, A. Rodriguez-Angeles, J. R. ReséNdiz and C. A. Cruz-Villar, "Concurrent Optimization for Selection and Control of AC Servomotors on the Powertrain of Industrial Robots," in IEEE Access, vol. 6, pp. 27923-27938, 2018, doi: 10.1109/ACCESS.2018.2840537
g) Cusimano G, Casolo F. An almost comprehensive approach for the choice of motor and transmission in mechatronic applications: Torque peak of the motor. Machines. 2021;9(8):159.

**Response:** We extend the literature review by adding synopses of the recommended research articles. A summary of changes made after reviewing the papers is presented here as well.

Our research focuses on designing a lightweight robotic manipulator from scratch, unlike studies (a) and (f) that address selecting off-the-shelf BLDC and AC servo motors. These prior works treated the selection as a multi-variable optimization problem, minimizing energy consumption, tracking error, and motor weight.

We propose a more holistic design methodology, where selecting a frameless BLDC motor is just one step. We use the standard AC7 block in Simulink/MATLAB to model the motor with manufacturer-provided data. Similarly, SolidWorks, with data sheet parameters, models the strain wave gears. These parameters are used in both the CAD model and the actuator model, which incorporates the motor, gears, and other components.

Custom-designed links complete the robot. Due to the diverse parts, our BLDC motor control analysis is less detailed. Our focus is establishing a framework for a lightweight robot using off-the-shelf components like frameless BLDC motors, strain wave gears, motor controllers, and custom links. Studies (b), (c), and (e) cover collaborative robot work cell design. Study (d) presents a virtual prototyping framework for rehabilitation exoskeletons. These are somewhat peripheral to our work.

Finally, study (g) describes a method for testing individual drive-transmission pairs, not considering them as a multi-DOF coupled system. While this method can analyze individual pairs, it lacks analysis for a complete system with interconnected joints.

**Comment # 3:** More specifications must be provided including torque saturations, peak and steady-state current limits, and controller gains for stability.

**Response:** For rapid component model configuration and simulation, we relied solely on the specifications provided in the component data sheets. This approach streamlines the process. We employed also the standard AC7 block within Simulink/MATLAB to model and drive the frameless BLDC motor. The hardware motor controller was then fine-tuned using the manufacturer's recommended method.

**Comment # 4:** In order to ensure clarity and accuracy, it would be greatly appreciated if a flowchart of the proposed methodology could be provided, highlighting the selected steps in yellow. This would aid in understanding the proposed methodology and facilitate the necessary review process.

**Response:** The requested flowchart is already provided in the manuscript. **Figure 1 illustrates the traditional design process for a mechatronic system, such as a robotic manipulator. The following explanation identifies the steps (highlighted with red bounding boxes) that our proposed methodology eliminates. Figure 3 then depicts the workflow of our proposed design and development cycle, starting from robot requirements and culminating in the completed robot hardware. For further clarity, the development of the subcomponent library is now detailed in the Methodology section, subsection 'Simulation Model Flow' of the revised manuscript.**

**Comment # 5:** At present, the stress analysis is carried out solely from a static point of view, with the maximum load applied to the final effector being the only consideration. However, the dynamic response of the system is influenced by the controller's gain tuning, and as such, the magnitude of values attained may surpass those observed in the static analysis.

**Response:** Static stress analysis with a high factor of safety (2.4) guarantees that the robot's structure can withstand dynamic stresses encountered during operation. In other words, this analysis validates the structural integrity of the robot links, ensuring they can endure the worst-case scenario without failure. This approach minimizes the risk of structural failure during robot operation.

**Comment # 6:** Can you please clarify which method was used to find the optimal solution for the built robot? Also, could you explain why some of the methods available in the Matlab libraries were not utilized? It would be helpful if the wording could be more precise since the introduction states that the optimization stage was eliminated.

**Response:** We achieved an optimal solution through an iterative design refinement process. This involved continuously improving the robot design until it achieved satisfactory performance, that is correlation coefficient greater than 0.95, on trajectory following test cases for a typical lightweight robot, as shown in Figure 12 and 14. While explicit optimization was not a primary focus in this stage of our research, we recognize its potential significance and intend to explore it further in future iterations of the development process. To validate functional performance, our criterion requires the physical prototype to achieve repeatability on the order of magnitude of the commercially available lightweight robot (+/- 0.05 for the Techman Robot TM5 robot, while ours is +/- 0.0503)

It is unclear from the referee’s comment what *additional* MATLAB libraries/tools could have been potentially useful for this study. We have utilized relevant MATLAB built-in models and CAD Modeling software. The objective was to create models capable of simulating performance that closely aligns with experimental outcomes. By assembling these models into a virtual prototype of the desired robotic manipulator, we aimed to demonstrate that the simulated performance would be comparable to the physical counterpart. As shown in the results section, the performance of the physically developed robot indeed matched that of the simulated model, validating our approach.

Our focus was on establishing the efficacy of using virtual prototypes built from models of commercially available components, thereby potentially bypassing costly and time-consuming physical design iterations. Additionally, the creation and maintenance of a library containing such components could further streamline the initial design phase.

**Comment # 7:** The proposed trajectories exhibit non-differentiability, thereby posing potential safety hazards for the cobot by introducing acceleration inconsistencies. This issue necessitates immediate attention and resolution to ensure the cobot's safe and optimal performance.

**Response:** The figure (Figure 1) below shows the magnified view of one of the velocity profiles. This establishes that the trajectories are continuous and *perfectly* differentiable. Perhaps a lengthy time axis spanning 17 seconds (Section III), squeezing the trajectory curve, may have given an illusion that the trajectories are discontinuous or non-differentiable, which is obviously not the case.

Figure 1 : (LEFT) Magnified view of the Velocity Trajectory signifying that a Trapezoidal Velocity Profile was used to drive the robot. (RIGHT) The trajectory as reported in the manuscript.

**Comment # 8:** The specifics of the simulation should be included, e.g., the tuning of the gains of each degree of freedom, the sampling time, and the solver used to solve the differential equations.

**Response:** The frameless BLDC motors were modeled and simulated using the AC7 block from the Simulink/MATLAB Specialized Power Systems Library. This block facilitates automatic tuning of the implemented PI controllers for position, velocity, and current control. The simulation was run with a fixed step size of 0.1 ms. The specific solver selection was left to Simulink, which by default utilizes the Backward Euler solver.

**Comment # 9:** A section should include all Solidworks parts and Matlab/Simscape files of the obtained design.

**Response:** All the relevant files will become part of the public repository as per the journal guidelines.

**Comment # 10:** It is recommended to enhance the quality of all images. Additionally, capture images where only the robot is visible.

**Response:** This comment is related to Fig 19. Quality/Resolution of all images is as per the journal specifications. Moreover, the images were taken in an R&D lab where the equipment visible in the image is part of the whole robotic system. Therefore, we believe it makes more sense to retain these images without modifications.

**Comment # 11:** Each of the figures should be described and discussed in detail in the text.

**Response:** The revised manuscript adequately addresses this concern. The explanation of the figures now appears after Table 4, closer to the graphs.

**Comment # 12:** Improve image quality. What is the maximum mass that the end effector can move? (Fig.27)

**Response:** The revised manuscript provides this information for both manipulators in appropriate sections.

**Comment # 13:** Could you kindly elaborate on what your goals are so that I can provide the most relevant insights? What are the criteria considered to design this robot?

**Response:** Introduction and other sections have been revised to address this comment. The criterion was reproduced in comment#6.

**Comment # 14:** Why use a correlation measure between data and not an error measure between the simulation and the physical prototype?

**Response:** Three standard metrics of trajectory error are:

1- Quantified Error Measure. It can be Mean Squared Error (MSE), Mean Absolute Error (MAE), Root Mean Squared Error (RMSE) etc.
2- Correlation Coefficient. This coefficient quantifies the linear relation of the reference trajectory with the measured trajectory.
3- Visual Comparison. Graphs of reference trajectories overlaid on the measured trajectories are used to represent the trajectory tracking error.

Originally, we used the Correlation Coefficient and the Visual Comparison for representation of the trajectory tracking error. However, to incorporate valuable reviewers’ suggestions, we have added the RMSE measure as well. We have calculated, stated, and discussed the RMSE for the position, velocity, and torque trajectories in the Hardware Experimentation section in the revised manuscript.

**Comment # 15:** Clarify what they mean by satisfactory performance. It helps refer to equations or constraints of the system.

**Response:** For optimal design we quantitatively explain the criterion of satisfactory performance in the last paragraph of Section II of the revised manuscript, as well as in the **response** to comment # 6. For the functional performance validation, our criterion requires that the physical prototype attains repeatability of the order of magnitude (ours is 0.05) same as that of the commercially available lightweight robot (0.05 for Techman Robot TM5).

**Comment # 16:** A section should include all Solidworks parts and Matlab/Simscape files of the obtained design.

**Response:** Already addressed in response to comment # 9.

**Comment # 17:** Web pages should be omitted as references, instead sources from indexed journals should be sought.

**Response:** The information extracted from the weblinks is not available in any published or indexed journals to the best of our knowledge. Moreover, there are no restrictions on referencing a website in PLOS ONE article.

**Comment # 18:** It needs to be understood what initial design methodology needs to be improved. In addition, it is necessary to add where the changes are made concerning the proposed method.

**Response:** The manuscript Section III subsection Simulation Model Flow and initial paragraphs of Section IV Simulation Experimentation details the design methodology. To highlight it further, we have updated parts of the Introduction and Section II as well.

**Comment # 19:** Review the rest of the observations in the attached file.

**Response:** We have reviewed the all the observations in the attached file and have tried our best to address all of them.

**REVIEWER # 4**

**Comment # 1:** The authors should add more quantitative data/analysis. The paper presents some quantitative results from simulations and experiments, but more data could strengthen the conclusions.
For example, providing specific statistics on the time/cost savings achieved using their method compared to traditional approaches, of course, based on explicit cites about the time/cost of traditional approaches.

**Response:** While we couldn't locate a directly comparable robotic arm case study in the literature regarding design cycle time cost savings, we've incorporated valuable insights from online resources. These resources provide general time allocation percentages for each design stage, further explained in the revised manuscript's Introduction section.

Our research offers a distinct contribution by presenting a quantitative analysis of time and human resource savings based on the robotic arm R&D conducted within our lab. This data, presented in Table 1 of the revised manuscript showcasing internal R&D man-hour savings, highlights the practical benefits of our proposed methodology.

**Comment # 2:** The authors use simple PI control but, the justification for this implementation is missing. It is common to use this type of controller for BLDC motors, but it is imperative to justify, at least with standard or state-of-the-art information about the advantages of PI over PD or PID controller for BLDC motors. On the other hand, the justification of using a PI controller can be done by results in the simulation of the system and get the justification that way.

**Response:** Our choice to simulate only a PI controller aligns with the practical reality that most commercially available BLDC motor controllers use PI control, as explained in the 'Frameless BLDC Motor Model' subsection of the Methodology section. Additionally, the same section discusses the advantages of PI control over PD or PID control for BLDC motors.

**Comment # 3:** It is imperative that the authors add data analysis between the simulation and experimental results regarding the trajectory tracking errors in terms of the error.
Additionally, add values of the tracking error in joint space between the reference and the measured values of the robot.

**Response:** The revised manuscript now includes a section on Hardware Experimentation that analyzes the trajectory tracking error in joint space between the reference and measured trajectories.

**REVIEWER # 5**

**Comment # 1:** All equations should be numbered and referenced when needed.

**Response:** We have numbered all the equations.

**Comment # 2:** The configured joints of the manipulator were configured with a damping constant of 2 Nm/deg/s, the usual SI damping units are Ns/m.

**Response:** Ns/m is the SI unit for Linear damping; however, Nm/rad/s is the SI unit for the rotational damping coefficient. Nm/deg/s has been changed to Nm/rad/s which is the SI unit for rotational damping coefficient.

**Comment # 3:** The iterated simulation using different integrated drive joints, does not yield an optimal joint as it’s mentioned.

**Response:** Our iterative design process begins by selecting commercially available, low-powered BLDC motors for the joints. If a joint fails to meet the desired trajectory due to motor torque or power limitations, we implement a power upgrade. This involves replacing the motor with the next higher-power motor that is commercially available. We continue this iterative process until the design achieves the required performance. For a more detailed response, please refer to our response to comment#6.

**Comment # 4:** Fig 8 does not show the Brushless DC Motor Drive (AC7) block and the connections used.

**Response:** Figure 8 shows only the Simulink SimScape Multibody model of the robot. Figure 11 shows the connection of the robot with the motors. Unfortunately, it is not possible to spawn the built-in macro and reveal the requested blocks, again, due to the limitations of the simulation tool.

**Comment # 5:** It is mentioned in the Abstract that the design cycle time is reduced by approximately 40% but no substantial evidence of this is explained during the results. Modifying the design approach does not inherently reduce the design cycle time since time saved in some areas may increase development time in other areas.

**Response:** The evidence is provided in Table 1 of the revised manuscript.

While it's true that changes can introduce unforeseen complexities, a well-chosen design approach can significantly reduce overall design cycle time, as demonstrated in this study. The history of the Mechatronics industry is a testament to this concept. Continuous exploration and adoption of new design methodologies have been a key driver of efficiency and innovation throughout the field's evolution.
