# Supplementary material for: Experimental development of lightweight manipulators with improved design cycle time that leverages off-the-shelf robotic arm components
Source: PLoS One. 2024 Jul 18;19(7):e0305379. doi: 10.1371/journal.pone.0305379 (PMC11257280; doi:10.1371/journal.pone.0305379)
Supplement: Attachment — Submitted filename: Response_to_Reviewers.docx [file pone.0305379.s005.docx]

Dear Editor,

We appreciate insightful comments and suggestions on our submitted manuscript. We take this opportunity to address the comments/concerns raised by the reviewers in the revised manuscript. Point-by-point responses are given below.

**Comments of Reviewer # 3 and Responses**

**Comment:** Concerning comment 3, what is suggested to be validated is that the manufacturer's restrictions (torque saturations, peak and steady-state current limits,) are not violated in the simulation and the physical system.

**Response:** Thank you for the valuable comment.

Under the violation of motor parameter limits, the MATLAB simulation either fails to proceed or throws a set of warnings; the real-controller stops after generating the error code. Neither occurred in our simulation and experiments generating data for the manuscript.

Because the chosen Simulink model, the standard AC7 block for BLDC motors, relies on manufacturer-provided parameters, it inherently captures the limitations of the actual motor. These parameters define the physical constraints of the real actuator. The details of this block are available at

<https://www.mathworks.com/help/sps/powersys/ref/brushlessdcmotordrive.html>.

**Comment:** Regarding comment 6, the author responds that they achieved an optimal solution through an iterative design refinement process. However, this process is subjective since there is no automatic process guiding the optimal solution; moreover, there is no guarantee of optimality. Furthermore, it would depend on the designer's expertise, so the validity of the timesaving would be questioned.

**Response:** Thank you for the comment.

Finding the absolute best design (globally optimal) can be challenging with automated optimization techniques. Our tool simplifies this challenge by generating a physical design that meets performance targets while adhering to real-world hardware limitations. This approach is demonstrated in the manuscript through a case study.

Furthermore, incorporating designer expertise through heuristics can accelerate the identification of very good (locally optimal) designs. This reduces the number of iterations required, leading to further time savings.

**Comment:** If the aim is to get a design, you may receive links larger than necessary and operate at relatively slow speeds, which may not reveal any issues. More reliable tests would involve displaying the design at ten times faster speeds, where dynamic effects can be seen and have a more significant impact. Moreover, you could suggest trajectories that work at resonance frequencies.

**Response:** Thank you for the valuable comment.

The input defines the maximum reach of the robot. Therefore, receiving links longer than necessary is not possible, otherwise it will violate the manipulator reach limit or the joint torque limit due to infeasibly large moment arm or link mass.

Lightweight robots are not designed to operate at arbitrarily high speed. Exposing dynamic effects at speeds higher than recommended speed is, therefore, not advisable. The trajectory on which the experimentation was done is of a typical industrial pick and place task and as the manipulator is a lightweight manipulator whose end-effector has speed limit (typically 1 m/s) if we must follow the ISO-TS/15066 standard for collaborative robots. This has been mentioned in the opening paragraph of the section Simulation Experimentation.

To suppress unmodeled underdamped resonance frequencies of the prototype we relied on fine-tuning the physical controller for the given trajectories. The tuning was guided by the simulation of the P-I controller that optimizes the relevant stability (gain, phase) margins. The hardware experimentations validate high trajectory tracking accuracy and low settling time, thereby showing that the impulsive jerk profile does not excite the vibrational modes of the robotic manipulator structure.

**Comment:** Until now, the only criterion to guide the validity of the design has been the robot's repeatability, which can be achieved with a well-tuned control and some possible feasible mechanical design solution.

**Response:** Thank you for the valuable comment.

The repeatability test serves a rigorous and the most widely used criterion to compare the performance of our prototype with that of commercial lightweight robots.

Yet, the desired specifications of the robot, provided at the start of the process (Fig. 3) serve as the main guideline for the process. The repeatability, trajectory tracking error, etc. are presented as criteria for establishing the operational performance of the designed robotic manipulator.

**Comment:** With respect to comment 7, it is suggested that the signals obtained in the accelerations and jerks, both from the references and from the experimental signals, be reviewed.

**Response:** Thank you for an insightful suggestion.

The real controller allows setting upper limit on the acceleration and jerk for tracing joint torque, velocity or position profile, if a jerk-limited type of motion is desired. However, for velocity and position control a non-jerk-limited mode allows a trapezoidal velocity profile to be followed. Should a joint actuator exceed the limits (e.g. due to insufficient power supply current), the controller aborts manipulator real-time trajectory following operation. These limits are given in the second para of the section Hardware Experimentation. In all our experiments, we used non-jerk-limited mode of the motor controller that implements trapezoidal velocity profile, while the joint acceleration and jerk remained within the set limits, the manipulator did not experience any operational glitches and controller did not throw any error code. Because the aim is to quantify trajectory tracking error as one of the performance measures, which holistically incorporates acceleration / jerk, we did not include separate plots of these quantities.

Alternatively, the presented torque profile (see figures 22 to 26) indirectly quantifies joint acceleration if we discount negligibly small joint damping and stiffness.

**Comment:** Regarding comment 17, reference 12 does not mention anything about 10%, and reference 14 only discusses time ranges for three tasks. This bullet source from your reference 14 also needs to be clarified (for example, 2 to 10 weeks). It says design and coding. How much of that time is dedicated to design, and how much to coding? Likewise, the percentages reported in the stage designs in this article should be indicated in the references (11-14), but this is not the case.

**Response:** We appreciate the referee for questioning the percentages reported in the manuscript.

The short answer is bootstrapping the numbers given lack of published data.

While we acknowledge the need for greater clarity regarding the mentioned percentages, it is important to note that the percentages presented were inferred from various sources, including referenced literature and typical R&D practices. As the field of robot design and development encompasses a wide array of activities, there may not be specific literature that universally quantifies the effort spent in each stage. To address this, we conducted a thorough review of multiple articles and recorded man-hours spent on each stage (Table 1) to ascertain these percentages. Our aim was to provide a comprehensive understanding of the potential impact of our proposed scheme on reducing the effort required in each stage of the design process. This approach ensures transparency and clarity regarding the origin of the percentages cited in the article.

**Comment:** It is still being determined where the percentages of the conventional design approach compared to the proposed design are obtained (Table 1). It also needs to be clarified how the authors guarantee time savings with the proposed methodology, given that no set of restrictions must be met to interrupt each of the design stages beyond having a correlation between the simulation and what is desired. For example, unlike what is proposed in reference 11, there is no talk of singularity analysis because there is no workspace analysis except doing a specific task.

**Response:** We thank the referee for querying the basis of time savings.

First, the Table-1 percentage savings come from the careful time-resource analysis of the R&D activities for the robotic arm development at Human Centered Robotics Lab at UET Lahore.

We have quantified the savings by comparing a very conservative estimate of time-resources at the beginning of the project (it was based on the conventional design approach) with the time-resource actually consumed (proposed methodology) in the R&D activities. Timely migration from the conventional to the proposed approach is an important part of our R&D journey.

Second, meeting the constraints the referee mentions, are in fact part of maximizing the correlation between simulation and what is desired, and it includes avoiding singularities. For example, simulations can reveal prohibitively large velocities as manipulator joint angles singular configurations. To prevent the robot from reaching singular configurations, which vary depending on the specific task it's performing, we implemented joint angle limitations, given in Table 5 of the revised manuscript. This restriction helps us avoid configurations requiring highly constrained joint movements (compact movements), thereby ensuring the robot's end-effector stays well within its reachable workspace and doesn't get dangerously close to the robot's own structure.

Lastly, time saving is guaranteed because of the use of simulated models of the commercialized component. Moreover, the structural components of a robotic manipulator have been designed and made part of the library. It means that the robotic manipulator design is now confined to selection of suitable components from the library instead of designing from the scratch. Restricting the choices to commercial components directly slashed design verification overhead in our case.

**Comments of Reviewer # 4 and Responses**

**Comment:** Please double-check the manuscript for any grammatical errors, typos, or spelling mistakes, and ensure there are no extra spaces between words or sentences.

**Response:** Thank you for bringing our attention to double-check the grammar. We have made best possible effort to correct the manuscript for typos and spacing errors.

**Comment:** If the design and development stages are different from the general percentage values presented in Fig. 1, which ones are the particular percentage values for this implementation?

**Response:** Thank you for the question.

The Table 1 (column 4) corresponds to effort required to complete each stage of the design and development process following the proposed scheme.

**Comment:** If Table 1 is one of the main results in this work, it should be addressed in a separate section of results and properly discussed, it should not be presented in the introduction because it demotivates the read of the article.

**Response:** Thank you for the valuable suggestion.

To establish a comparison with the percentage of design cycle time consumption at different stages of the design and development process, Table 1 has been presented in the introduction. Moreover, in the explanation of Table 1 it has been stated that these values may differ from those presented in Fig 1, because Fig 1 presents generalized values of percentage of effort spent in each stage of an engineering design cycle.

**Comment:** In Figs 13 to 17 are presented the references of each joint. I recommend adding the equations of the references proposed and also add the acceleration profile of each joint. The joint velocity profile in Figs 13 to 17 seems to be nondifferentiable. I recommend reading the book by Spong, M. W., Hutchinson, S., & Vidyasagar, M. (2020). Robot modeling and control. John Wiley & Sons. Chapter 7 is about path planning and explains why discontinuities in acceleration should be avoided.

**Response:** Thank you for sharing the recommended literature and highlighting the importance of importance of avoiding discontinuities in the state variables.

First, there are no discontinuities in joint velocities and acceleration. The figure (Figure r1) below shows the magnified view of one of the velocity profiles sampled from Fig.13 to Fig.17. The enlarged view (of a likely misunderstood discontinuity between time 0-0.6s) establishes that the trajectories are continuous and *perfectly* differentiable. Perhaps a lengthy time axis spanning 17 seconds (Section III), squeezing the trajectory curve, may give an illusion that the trajectories are discontinuous or non-differentiable, which is obviously not the case.

We were unable to generate a concise or meaningful analytical form of our differentiable trajectories. On the other hand, the graphical description adequately captures essential features of the reference joint-space trajectories.

The suggested book on Robotics explains the path planning as well as trajectory planning. Its chapter 8 discusses trajectory planning and points out that if multiple trajectories are blended to get a continuous position and velocity trajectories, then one might get discontinuous acceleration trajectories. This will lead to impulsive jerk trajectory that may excite vibrational modes in the manipulator hence reducing the trajectory tracking efficiency. The same chapter introduces Linear Segment with Parabolic Blend (LSPB) trajectory, also called Trapezoidal Velocity Profile trajectory and it is used for our simulations given in Section III. This trajectory has a constant acceleration profile with impulsive jerk profile.

The hardware experimentation results show the trajectory tracking accuracy, which proves that the impulsive jerk profile is not exciting the vibrational modes of the robotic manipulator structure. Furthermore, our experimentation which tests the manipulator on very standard types of trajectories, generate joint torque profile which is much more insightful as compared to the acceleration and jerk profiles.

*Figure r1 : (LEFT) Magnified view of the Velocity Trajectory signifying that a Trapezoidal Velocity Profile was used to drive the robot. (RIGHT) The trajectory as reported in the manuscript.*
